# Behavioral and Neuroimaging Research on Developmental Coordination Disorder (DCD): A Combined Systematic Review and Meta-Analysis of Recent Findings

**DOI:** 10.3389/fpsyg.2022.809455

**Published:** 2022-01-27

**Authors:** Emily Subara-Zukic, Michael H. Cole, Thomas B. McGuckian, Bert Steenbergen, Dido Green, Bouwien CM Smits-Engelsman, Jessica M. Lust, Reza Abdollahipour, Erik Domellöf, Frederik J. A. Deconinck, Rainer Blank, Peter H. Wilson

**Affiliations:** ^1^Healthy Brain and Mind Research Centre, School of Behavioural and Health Sciences, Faculty of Health Sciences, Australian Catholic University, Melbourne, VIC, Australia; ^2^Department of Pedagogical and Educational Sciences, Behavioural Science Institute, Radboud University, Nijmegen, Netherlands; ^3^Department of Health Sciences, Brunel University London, London, United Kingdom; ^4^Department of Rehabilitation, School of Health and Welfare, Jönköping University, Jönköping, Sweden; ^5^Division of Physiotherapy, Department of Health and Rehabilitation Sciences, Faculty of Health Sciences, University of Cape Town, Cape Town, South Africa; ^6^Department of Natural Sciences in Kinanthropology, Faculty of Physical Culture, Palacký University Olomouc, Olomouc, Czechia; ^7^Department of Psychology, Umeå University, Umeå, Sweden; ^8^Department of Movement and Sports Sciences, Ghent University, Ghent, Belgium; ^9^Heidelberg University, Heidelberg, Germany; ^10^Klinik für Kinderneurologie und Sozialpädiatrie, Kinderzentrum Maulbronn gGmbH, Maulbronn, Germany

**Keywords:** Developmental Coordination Disorder (DCD), neurodevelopmental disorders, meta-analysis, motor learning and control, executive function, cognitive control, neuroimaging

## Abstract

**Aim:**

The neurocognitive basis of Developmental Coordination Disorder (DCD; or motor clumsiness) remains an issue of continued debate. This combined systematic review and meta-analysis provides a synthesis of recent experimental studies on the motor control, cognitive, and neural underpinnings of DCD.

**Methods:**

The review included all published work conducted since September 2016 and up to April 2021. One-hundred papers with a DCD-Control comparison were included, with 1,374 effect sizes entered into a multi-level meta-analysis.

**Results:**

The most profound deficits were shown in: voluntary gaze control during movement; cognitive-motor integration; practice-/context-dependent motor learning; internal modeling; more variable movement kinematics/kinetics; larger safety margins when locomoting, and atypical neural structure and function across sensori-motor and prefrontal regions.

**Interpretation:**

Taken together, these results on DCD suggest fundamental deficits in visual-motor mapping and cognitive-motor integration, and abnormal maturation of motor networks, but also areas of pragmatic compensation for motor control deficits. Implications for current theory, future research, and evidence-based practice are discussed.

**Systematic Review Registration:**

PROSPERO, identifier: CRD42020185444.

## Introduction

### Overview

Developmental Coordination Disorder (DCD) is a chronic and pervasive neurodevelopmental disorder that is defined by an impaired ability to acquire age-appropriate levels of motor skill (Barnhart et al., 2003; American Psychiatric Association, 2013). The disorder is characterized by awkward, inefficient, and often slow performance of fine- and/or gross-motor movements (Barnhart et al., 2003), including everyday actions like tying shoelaces, doing up buttons, handwriting, and participating in leisure activities or organized sport. Developmental Coordination Disorders prominence is shown by a high 5–6% prevalence rate in school-age children and common co-occurrence with other disorders such as autism spectrum disorder (ASD) and attention deficit/hyperactivity disorder (ADHD) (Barnhart et al., 2003; American Psychiatric Association, 2013). The disorder also persists well into adolescence and early adulthood in around half of those first diagnosed in childhood (Cousins and Smyth, 2003; Kirby et al., 2008), and has associated difficulties with academic achievement, psychosocial adjustment, physical health, and wellbeing (Gillberg and Kadesjö, 2003; Green et al., 2006; Zwicker et al., 2013; Kirby et al., 2014). Therefore, understanding the underlying basis of the motor difficulties is critically important for theory and ultimately the development of effective interventions. This review is another step in that direction, synthesizing the body of experimental work conducted over the past 5 years.

The conceptual foundation for much of the current work on DCD (and for the meta-analytic review presented here) is integrative, blending constructs from (i) cognitivist, (ii) cognitive neuroscience (McNamee and Wolpert, 2019), and (iii) ecological-systems theory (Adolph, 2019) to better model and understand the various constraints on motor behavior (Gentsch et al., 2016; Wilson et al., 2017b). Indeed, over time, these approaches have coalesced around an integrated (or hybrid) approach to the study of DCD (Wilson et al., 2017a). This (multi-component) hybrid model identifies that motor performance is the consequence of interactions between constraints at an individual, task, and environmental level. Emerging motor competency will therefore be the result of the *individual constraints* (e.g., physical maturation, genetic make-up, neurocognitive mechanisms, and psychological characteristics), *environmental constraints* (e.g., opportunities for practice, physical education, and sociocultural context), and *task-related constraints* (e.g., the rules of the game, goals of the task, equipment used). Similarly, the development of basic motor control and learning processes (at the individual level) may be expressed behaviorally in different ways as a function of task and environmental factors. For example, the motor and cognitive control needed to tie shoelaces at home, seated, is very different from tying shoelaces in a busy classroom at school while trying to follow the conversation of peers.

While there appears to be no single causal agent in the etiology of DCD, a variety of factors has been implicated in earlier research and highlighted in the most recent international consensus reviews of the DCD literature. Those factors identified include deficits in predictive motor control and skill automaticity (aka *internal modeling deficit* hypothesis—IMD) (Tsai et al., 2009; Wilson et al., 2013, 2017a; Adams et al., 2014), perceptual-motor coupling, executive function, atypical neural structure and function in networks that support motor planning and imitation, including the *mirror neuron system* (MNS) (Brown-Lum and Zwicker, 2015; Biotteau et al., 2016), and atypical inter-hemispheric communication (Sigmundsson et al., 1999; Tallet et al., 2013). More specifically, the IMD account of DCD posits a core deficit in the ability to implement predictive models of action, which impacts the online adjustment of movements in response to external perturbations and impairs movement automatization (Wilson et al., 2013, 2017b; Adams et al., 2014). Deficits of this type have been observed across a number of effector systems (oculomotor, manual, and postural control) (Wilson et al., 2013, 2017b; Adams et al., 2014). The MNS account is supported by converging data showing deficits in motor imagery (MI) and observational learning, with downstream effects on the ability of children with DCD to acquire novel motor skills (Reynolds et al., 2015; Lust et al., 2019). Finally, a maturational delay in the development of inter-hemispheric communication has been suggested by work showing impaired bimanual motor coordination and altered inter-hemispheric coupling through the corpus callosum (Sigmundsson et al., 1999; Tallet et al., 2013). This possible mechanism has profound implications for motor learning in DCD (Blais et al., 2018; Tallet and Wilson, 2020). However, there are caveats on the generalisability of each of these hypotheses.

A complication for current accounts of DCD is the undeniable fact that many of these putative deficits are expressed differently. They vary as a function of specific individual, task and environmental constraints, e.g., co-occurring disorders such as ADHD or ASD, the specific effector systems involved, the complexity of motor action, the degree of endpoint precision, the type and level of cognitive involvement, and the presence (or not) of environmental distractors (Wilson et al., 2017a). As well, deficits in executive function are shown to be pervasive across its core domains, which raises the possibility that DCD should be considered a disorder of motor-cognitive function in a significant group of individuals meeting the clinical diagnosis. What remains unclear, however, is the question of how motor and cognitive processes are integrated in real-time during goal-directed action, and over the course of repeated practice (or learning) and under differing contexts.

Over the past 5 years, DCD researchers have endeavored to address these outstanding issues by use of more refined task paradigms that offer greater internal and ecological validity, and integrate behavioral and brain-based measures. For example, in the context of cognitive-motor integration, behavioral research has begun to explore the time course where cognitive control can be exerted in a movement task (Suzuki et al., 2020). A number of recent neuroimaging studies have begun to target their investigations on specific sensorimotor brain networks that underpin motor control (Reynolds et al., 2017b; Williams et al., 2017; Thornton et al., 2018; Brown-Lum et al., 2020; Rinat et al., 2020). To date, the entire body of most recent behavioral and neuroimaging work has not been critiqued and synthesized using a combined systematic and quantitative review, but rather several smaller reviews have been conducted in niche areas of function, such as gait, postural control, and aspects of neural activation during manual action. The next section summarizes the findings of these reviews, which provides a clear departure point for the current review.

### Recent Focused Meta-Analyses of Gait, Posture, and Neural Function

Since the latest consensus review (Wilson et al., 2017b), two focused meta-analyses on DCD have been conducted on behavioral measures of gait (Smith et al., 2021) and balance control (Verbecque et al., 2021). The analysis by Smith et al. (2021) included five studies that used the 6-Minute Walking Test (6MWT) to evaluate differences on gait and fitness outcomes between DCD and *typically developing children* (TDC). Results showed functional deficits in DCD on measures of endurance and cardiorespiratory fitness, but no clear signature in gait pattern for DCD was evident. The analysis did report high heterogeneity in effect size outcomes, and low study quality, overall.

The meta-analysis of Verbecque et al. (2021) included eight studies on postural control (both anticipatory and reactive), stability, and balance under varying sensory conditions. Pooled results showed a delayed onset in reactive and anticipatory control adjustments, with larger sway under more complex sensory and environmental conditions. During simple tasks, children with DCD were able to adjust for errors, however, during difficult tasks, which called for greater anticipatory control and complex sensory integration, these children were slower and less efficient in using feedback-based control. These findings were said to be broadly consistent with the IMD hypothesis of DCD. A limitation of this meta-analysis, however, was the dedicated focus on specific outcome measures [e.g., Center of Pressure (COP) excursions and muscle-onset latencies] and neglect of cognitive and neural mechanisms (e.g., cerebellum and supplementary motor area). Collectively, these two meta-analytic studies examined focused aspects of gait and balance control in DCD, included papers published before 2018, and predominantly analyzed work between 2010 and 2015 (Smith et al., 2021; Verbecque et al., 2021).

The latest neuroimaging research suggests disruptions to both neural structure and functional activation patterns in children with DCD. A recent review by Biotteau et al. (2016) evaluated 14 studies that used structural *magnetic resonance imaging* (MRI), functional MRI (fMRI), and *diffusion tensor imaging* (DTI) in order to clarify a neural signature for children with DCD. Notably, fMRI results revealed cerebellar dysfunction and reduced parietal lobe activation patterns in children with DCD. Fronto-parietal and fronto-cerebellar networks have critical roles in motor planning, motor control (esp. internal modeling), visual-motor mapping, and automatization. Furthermore, they overlap the MNS and may, therefore, explain the combined motor control and learning deficits seen in DCD. There were, however, a number of limitations with the studies reviewed: only six sMRI studies were completed prior to 2016; an absence of common tasks across fMRI studies; limited number of parametric designs, and the small/heterogenous samples tested. Biotteau et al. (2016) were, hence, unable to confirm a neural signature in DCD that stands alone from other neurodevelopmental disorders.

A related Activation Likelihood Estimation (ALE) meta-analysis, by Fuelscher et al. (2018) evaluated atypical neural (de)activation in children with DCD. This ALE meta-analysis identified reduced activation within the fronto-cerebellar axis and posterior cerebellar regions. These regions are a critical part of networks that support online motor control, motor learning, and integration of higher-order cognitive control. The authors concluded that the pattern of reduced activation may reflect the recruitment of executive control systems and noted the task paradigms considered did not include probes of internal modeling. Fuelscher et al. (2018) noted reduced activation within the right supramarginal gyrus of the parietal lobe, a core component of the MNS, and strongly implicated in the perception of somato-sensory stimuli and phonological processing. Fuelscher also identified hypo-activation of the fronto-motor regions which support the planning, execution, and control of voluntary movements. By comparison, enhanced thalamic activation (esp. pulvinar) in DCD was observed, perhaps reflecting greater reliance on visual feedback during motor sequencing tasks (Fuelscher et al., 2018). Taken together, these most recent reviews of neuroimaging work identify associations with the MNS and other networks that underpin the cognitive control of action. However, limitations in the design and scale of these studies suggests caution in drawing conclusions about neural correlates of DCD. The impetus behind the current review is the large number of neuroimaging studies published between 2016 and 2021, begging a re-appraisal of this literature.

### Objectives for the Current Review

Over the past 5 years, significant advances were observed in the methodological quality of movement research and quantity of research on mechanisms of DCD (with nearly 100 studies published since September 2016). The meta-analyses described above have been very focused, exclusive of recent work, and not designed to pinpoint distinct patterns in the performance profiles of those with DCD across task domains and paradigms, or clarify their neural basis. We have the benefit, however, of very recent studies using a more integrative approach to investigation (with an eye to both internal and ecological validity), higher quality neuroimaging tools, and operationalisation of constructs using combined behavioral and neural measures. In short, there is a strong rationale for analyzing the entire corpus of literature on DCD over the past 5 years, providing further insight into its underlying mechanisms. Our broad aim was, therefore, to conduct a combined systematic review and meta-analysis to clarify the following: (i) the profile of motor control and learning deficits in DCD across different domains of performance; (ii) the profile of deficits across different domains of cognitive control, and in cognitive-motor integration, and (iii) disruptions in brain structure, function and/or maturation, and their association to the motor behavior of individuals with DCD compared with typically developing peers.

## Methods

### Literature Search

This review was pre-registered with PROSPERO (registration number CRD42020185444) and the literature search was conducted in accordance with PRISMA guidelines (Moher et al., 2015). The protocol details can be accessed at https://www.crd.york.ac.uk/PROSPERO/display_record.php?RecordID=185444 and a PRISMA checklist is provided in Supplementary Material 1.

#### Search Strategy

A multi-database systematic literature search was conducted from September 2016 up to April 1, 2021, using the following electronic databases: Medline Complete, APA PsycINFO, APA PsycArticles, Psychology and Behavioral Sciences, CINAHL Complete, Scopus, Embase, and Web of Science. A modified PICOS framework was used as follows: (1) Population: anyone with DSM-5 Diagnosed DCD or those who meet the minimum research criteria for probable DCD (pDCD: low level of motor competence relative to peers on a standardized test, plus confirmation of motor difficulties by teacher/parent); (2) Interest: any behavioral (i.e., motor or cognitive) or neuroimaging outcome; (3) Comparison: DCD group compared with typically developing controls, in any age cohort; (4) Outcome: measures of motor control, motor learning, or cognitive function, and neuroimaging markers, and (5) Study type: experimental designs comparing DCD and TD groups. Developmental Coordination Disorder groups with co-occurring disorders were not excluded. An inclusive search string was adopted, consisting of synonymous population terms present in a Title and Abstract search: (“developmental coordination disorder” or “minimal brain dysfunction” or “minimal neurological dysfunction” or “developmental dyspraxia” or “perceptual-motor disorder” or “specific developmental disorder of motor function” or “clumsy child^*^”). Additional articles were identified by screening other journal papers that cited the included studies in Google Scholar.

### Selection Criteria

Studies were required to fulfill the following selection criteria for inclusion in the review: (a) an experimental case-control, cross-sectional or longitudinal study design; (b) publication in a peer reviewed journal; (c) reported measures of behavioral performance (including motor control, motor learning, or cognition), or neuroimaging metrics (including MRI, EEG, and related); (d) report sufficient statistical information to calculate effect sizes.

Studies were excluded if: (a) original data were not reported (i.e., if reported elsewhere, in a review, meta-analysis, or commentary paper); (b) the article was not written in English; (c) the article was included in the earlier consensus review by Wilson et al. (2017b); (d) participants were exposed to an intervention. The first author (E.S.) executed the searches through each database and the articles were double screened by E.S. and P.W.

### Evaluation of Methodological Quality

Included studies were assessed for quality utilizing a modified version of the Critical Appraisal Skills Programme for case-control studies [*Critical Appraisal Skills Programme (CASP)—Case-Control Study Checklist*., CASP, 2021] (Supplementary Material 2) by two independent authors, including the lead author (E.S.) and a co-author with proven expertise relevant to the topic of focus in the study. For each study, each of 10 CASP items was scored as low (1), some concerns (0.5), or high (0) risk of bias, providing a total score out of 10. Any differences in ratings between the authors were discussed and, if consensus could not be reached, a third author was enlisted as a moderator; a final decision was reached by consensus. The studies with a total score of eight or above were identified as high quality (i.e., low risk of bias), scores between 5.5 and 7.5 were moderate, and a score of five or less as low quality (i.e., high risk of bias) (Wilson et al., 2017b).

### Data Extraction and Management

The data were extracted independently by the allocated expert author using a standardized extraction spreadsheet in Excel^TM^ (MS Inc.) format, including a supplementary coding sheet based on that used previously by Wilson et al. (2013) (Supplementary Material 3). Information regarding sample characteristics, screening tools, task domain/category, task paradigm, experimental conditions, type of outcome measure, and results in the form of descriptive and parametric statistics were obtained from each study. Data extraction was cross-checked by either E.S. or T.M., with any discrepancies resolved through discussion.

### Study Coding

The categorization of performance tasks was reached by consensus agreement between the lead and co-authors, each of whom has proven expertise in one or more areas relevant to this research on DCD. The process of consultation between authors involved a combination of videoconference (via MS teams and zoom) and face-to-face meetings between the australian-based members of the team. working documents were shared via Email and WeTransfer.

Studies were first grouped according to the dominant theoretical framework informing their design: (1) cognitivist, (2) dynamical/ecological, and (3) cognitive neuroscience and related hybrid approaches (including neuroimaging). Under each framework, the experimental tasks were then categorized into performance domains/categories (e.g., gait; target-directed reaching under the hybrid approach) or the underlying construct of focus (e.g., executive functions under cognitivist approach; oculomotor control under hybrid). Under these categories, constructs were sub-categorized based on specific task parameters (e.g., cued and un-cued reaching) and types of outcome measure (e.g., spatial and temporal gait metrics)—see Supplementary Material 3.

### Meta-Analysis Procedures

All statistical analyses were conducted using *R version 4.0.5* (R Core Team, 2021) and *RStudio* (R Studio Team, 2020).

#### Calculation of Effect Size

Calculation of effect size (Cohen's *d)* was conducted for each DCD-TDC comparison. The following assumptions were made with respect to results that were not reported in full detail in the paper (see also Wilson et al., 2013). If the result of a parametric test was not reported or reported as not significant (NS), a *p*-value of 0.50 was assumed. When a statistically significant group difference was reported without accompanying statistics, a *p*-value of 0.05 was assumed. When a statistical result was reported imprecisely (e.g., *p* < 0.01), the *p*-value was assumed to be the value reported (i.e., *p* = 0.01). It is recognized that these assumptions are likely to result in more conservative effects.

When available, the reported means, standard deviations and sample sizes for each group were used to calculate effect sizes using the *esc* package (Lüdecke, 2019). *SE*-values were converted to *SD*-values, if necessary. In the absence of these data, estimates of effect size were calculated using the *compute.es* package (Del Re, 2013) by using sample size and *F, t*, or *p*-values.

#### Quantitative Synthesis

The *metafor* (Viechtbauer, 2010) package was used to conduct meta-analyses using multi-level random-effects models (Assink and Wibbelink, 2016; Cheung, 2019). A multi-level meta-analysis is preferred when multiple effect sizes are nested within (i.e., extracted from) individual studies, such as in the present review (Konstantopoulos, 2011; López-López et al., 2018; Fernández-Castilla et al., 2020). This multi-level approach allows each effect size to be included in the model while accounting for the non-independence of effect sizes (Konstantopoulos, 2011; López-López et al., 2018; Cheung, 2019; Gucciardi et al., 2021). Further, this approach allows the interpretation of heterogeneity at both level-2 (i.e., effect size) and level-3 (i.e., study) with *I*^2^ statistics (Higgins and Thompson, 2002; Cohen, 2013), completed using the *dmetar* package (Harrer et al., 2019). The Hartung-Knapp-Sidik-Jonkman method was used to estimate the variance of the pooled effect as it outperforms other methods when there are few studies or substantial heterogeneity (Hartung, 1999; Hartung and Knapp, 2001; IntHout et al., 2014). Substantial heterogeneity is generally indicated when *I*^2^ exceeds 75% (Higgins and Thompson, 2002; Huedo-Medina et al., 2006). However, use of a random-effects model does attenuate the impact of heterogeneity, i.e., those combined ESs that are impacted can still be interpreted with reasonable confidence under caveats. Notwithstanding this, for those performance categories that showed substantial heterogeneity in ESs (*I*^2^ > 75%), we considered the feasibility of moderator analysis based on the following: first, whether there was a sufficient number of ES entries for such analysis and, second, whether there was a sound conceptual reason to split such categories and re-compute combined ESs. In most cases there were too few ESs to conduct a meaningful moderator analysis, or no evident moderator that would resolve heterogeneity. In two instances, after consultation between lead and co-authors, categories were split, which resolved to the final coding scheme presented in our paper. In sum, we accepted the final meta-analytic solution under the caveat that heterogeneity (for some categories) does reflect an element of unresolved error in the combined ES estimate. The *anova* function was used to compare the overall three-level model with the overall two-level model. Significant AIC and BIC outcomes showed that the multi-level analysis was better able to capture variability of the nested data than two-level random-effects models.

Combined ESs were calculated at the main performance category and sub-category level, with positive values indicating a more favorable outcome for the typically developing control group. Significant combined (or mean) ESs were indicated by 95% confidence intervals that did not cross zero. The magnitude of each combined estimate was interpreted according to conventions of Cohen (2013): 0.3 (small), 0.5 (moderate), 0.8 (large), >1.0 (very large).

## Results

**Figure 1:**
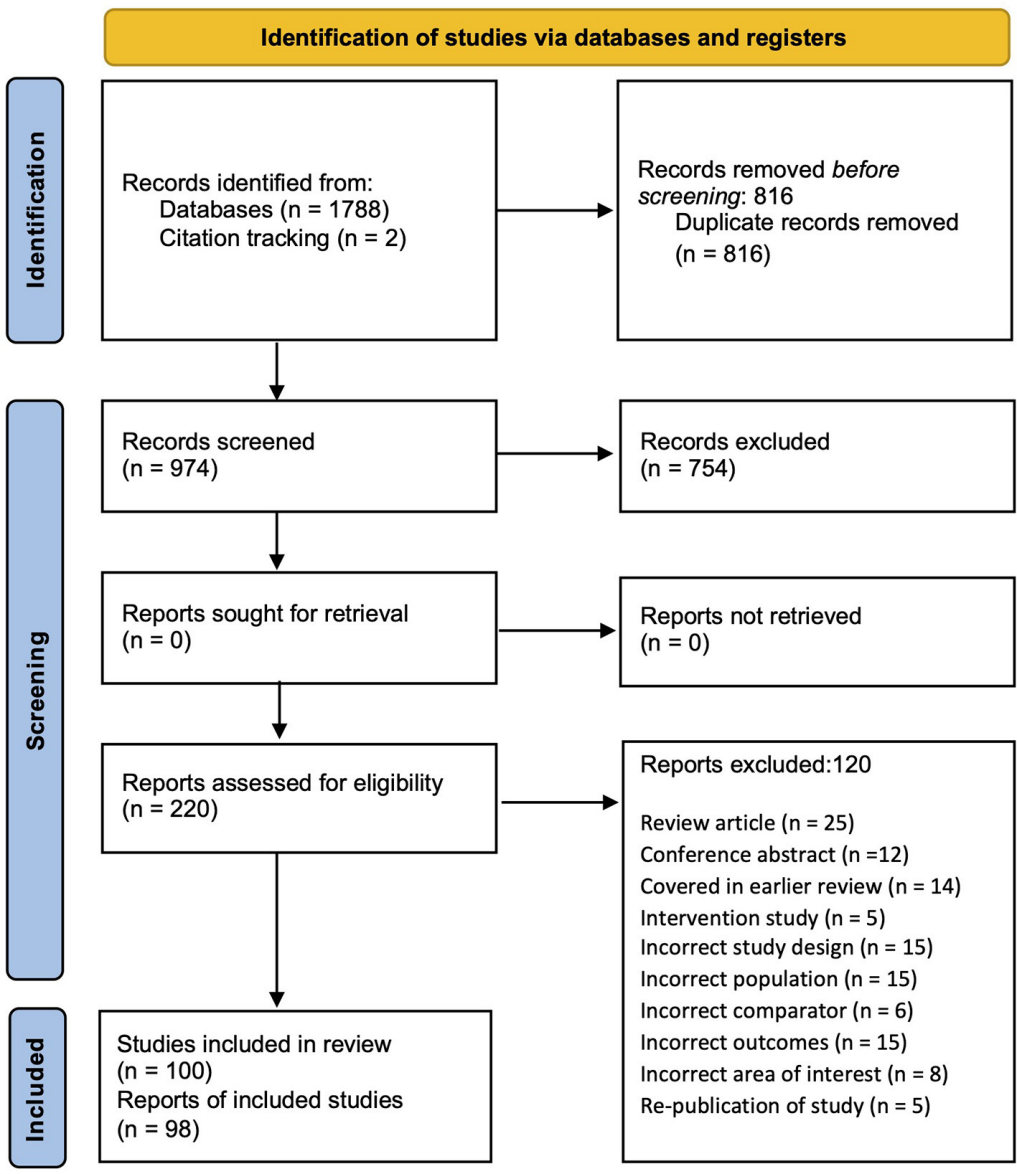
PRISMA flow diagram for the systematic review process.

**Figure 2:**
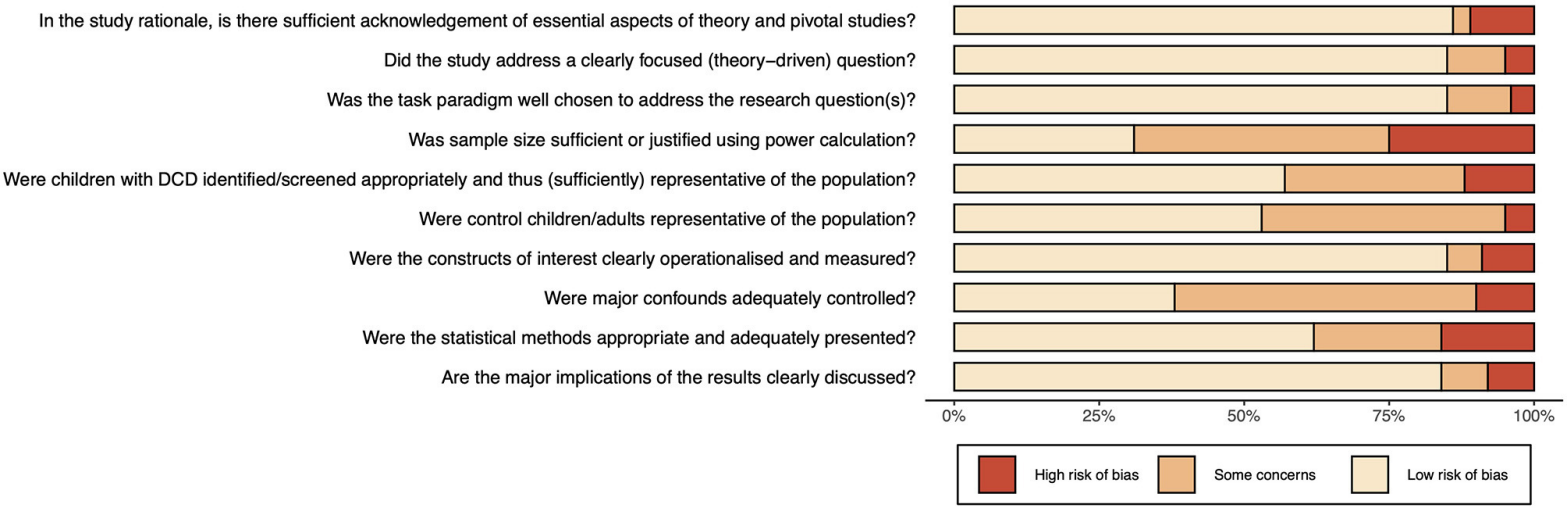
Risk of bias plot using the modified CASP criteria.

**Figure 3:**
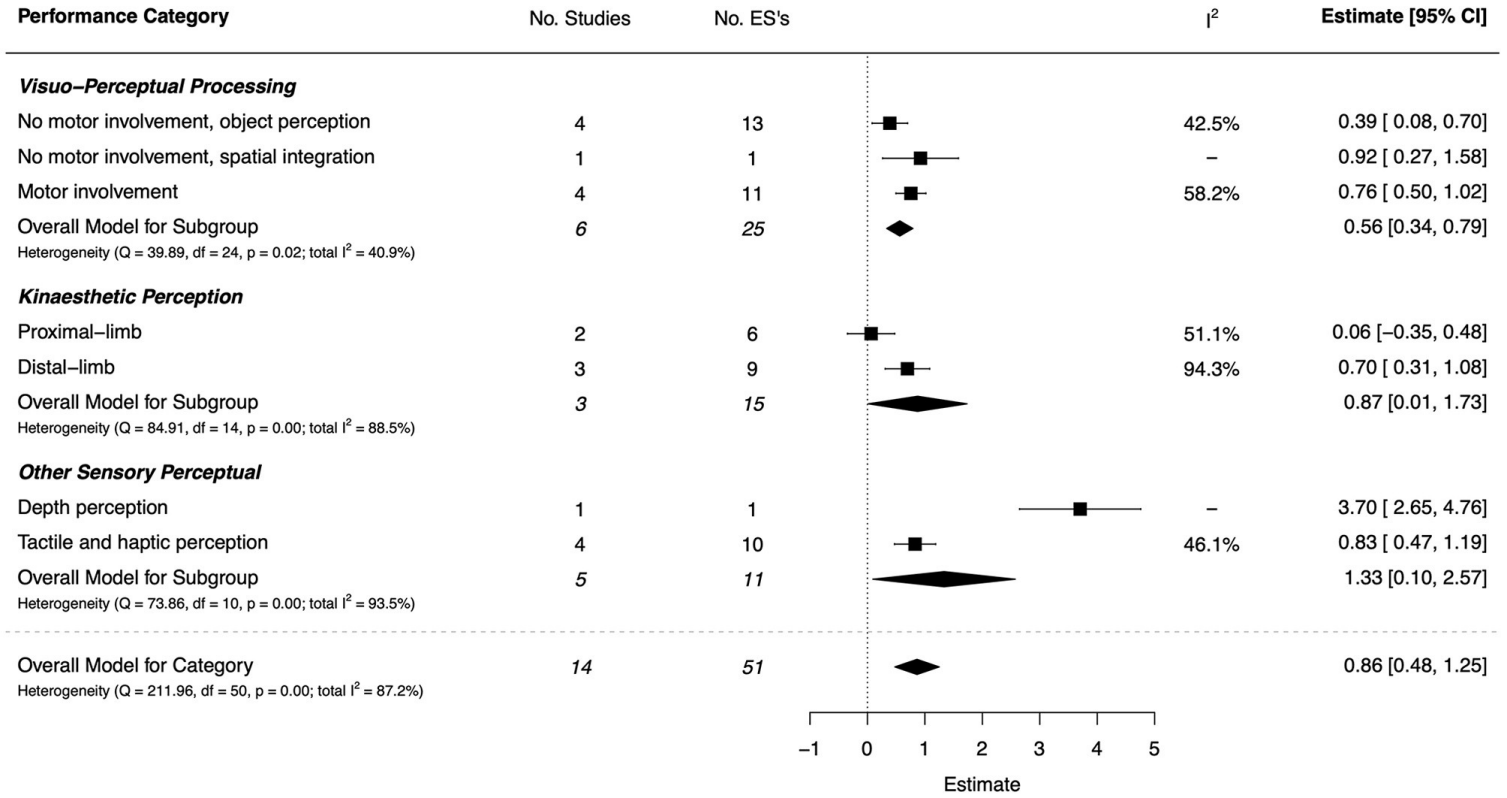
Forest plot showing the meta-analysis results for the sensori-perceptual function performance category.

**Figure 4:**
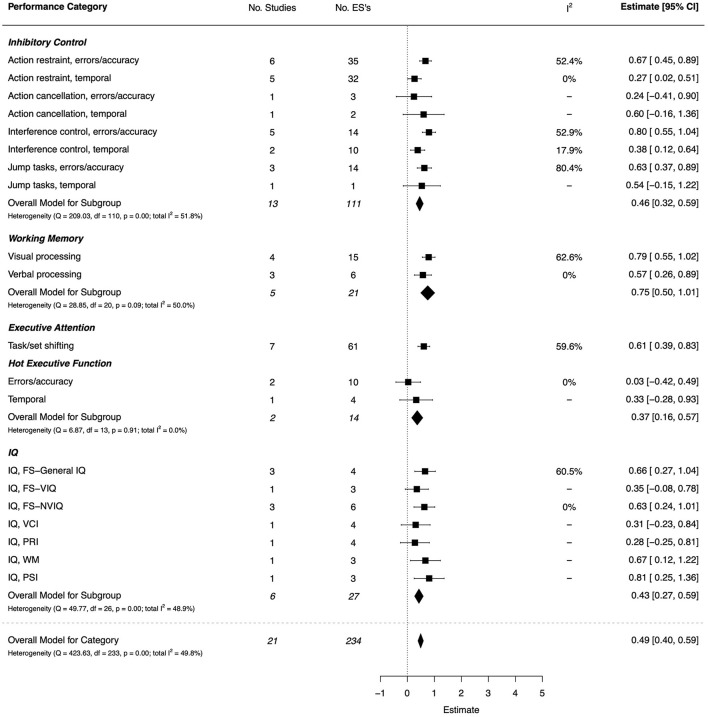
Forest plot showing the meta-analysis results for the executive functions and intelligence factors performance category. IQ, Intelligence Quotient; FS, Full-Scale; VIQ, Verbal IQ; NVIQ, Non-Verbal IQ; VCI, Verbal Comprehension Index; PRI, Perceptual Reasoning Index; WMI, Working Memory Index; PSI, Processing Speed Index.

**Figure 5:**
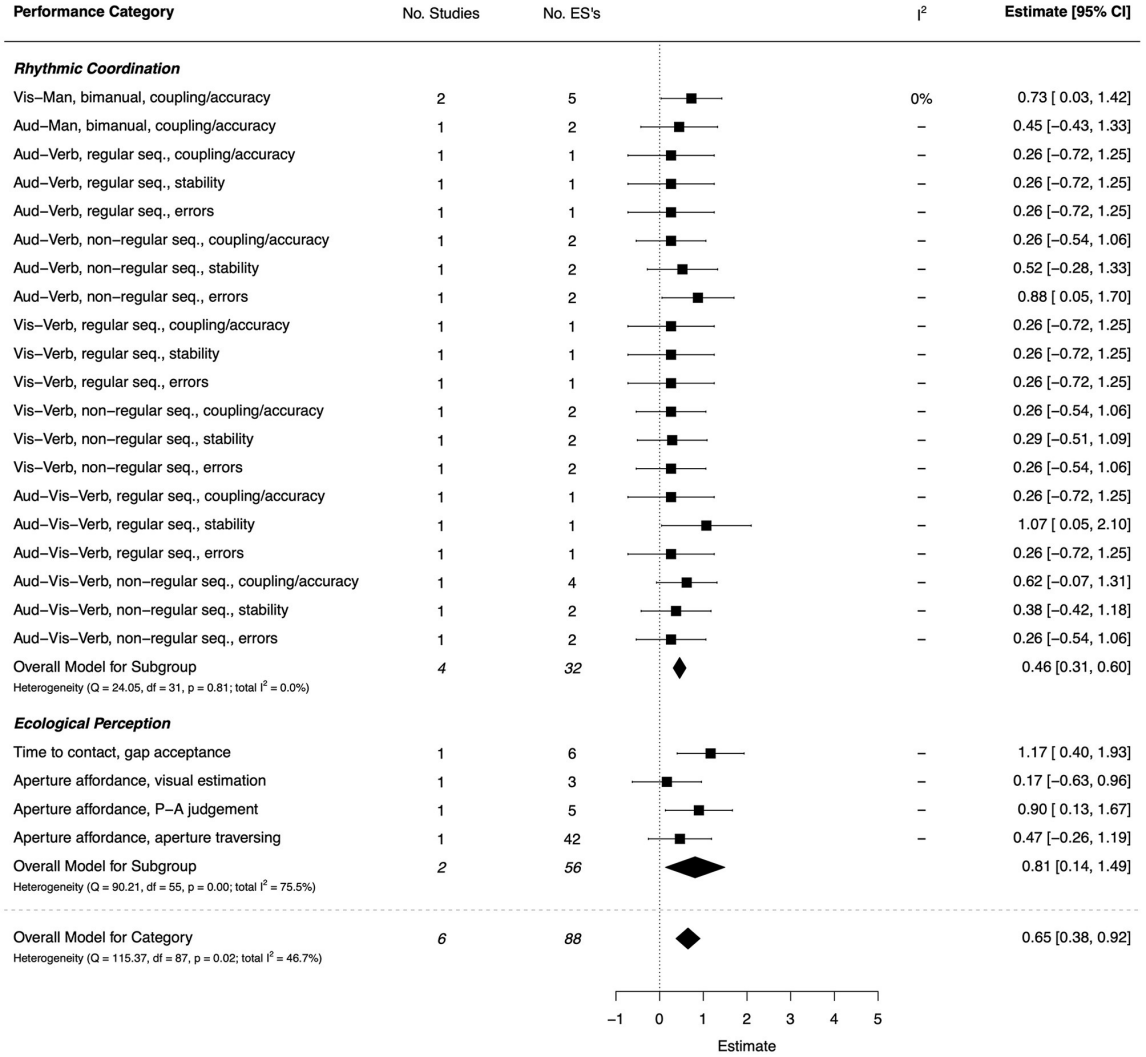
Forest plot showing the meta-analysis results for the dynamical/ecological paradigms performance category. Vis-Man, Visual-Manual; Aud-Man, Auditory-Manual; Aud-Verb, Auditory-Verbal; Vis-Verb, Visual-Verbal; Aud-Vis-Verb, Auditory-Visual-Verbal; Seq., Sequence; P-A, Perception-Action.

**Figure 6:**
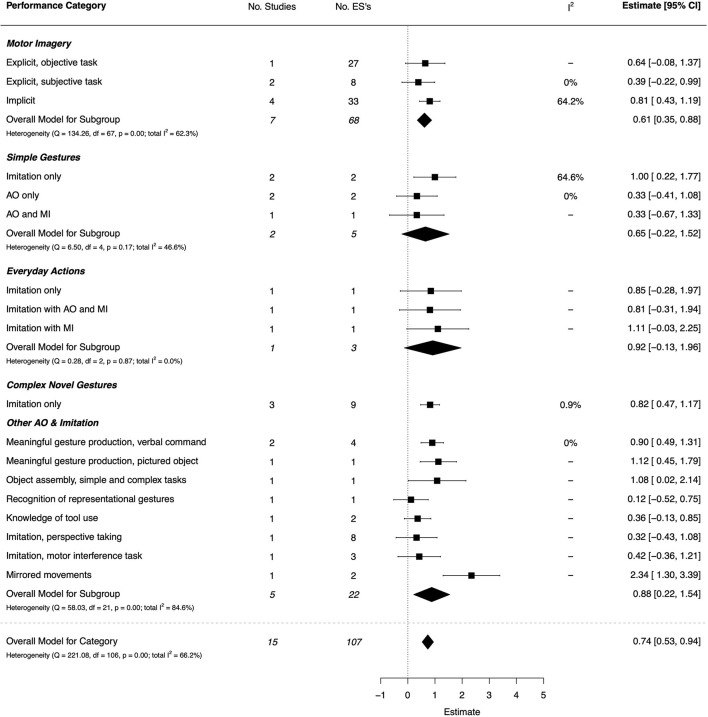
Forest plot showing the meta-analysis results for the motor imagery, action observation, imitation, and gesture production performance category. AO, Action Observation; MI, Motor Imagery.

**Figure 7:**
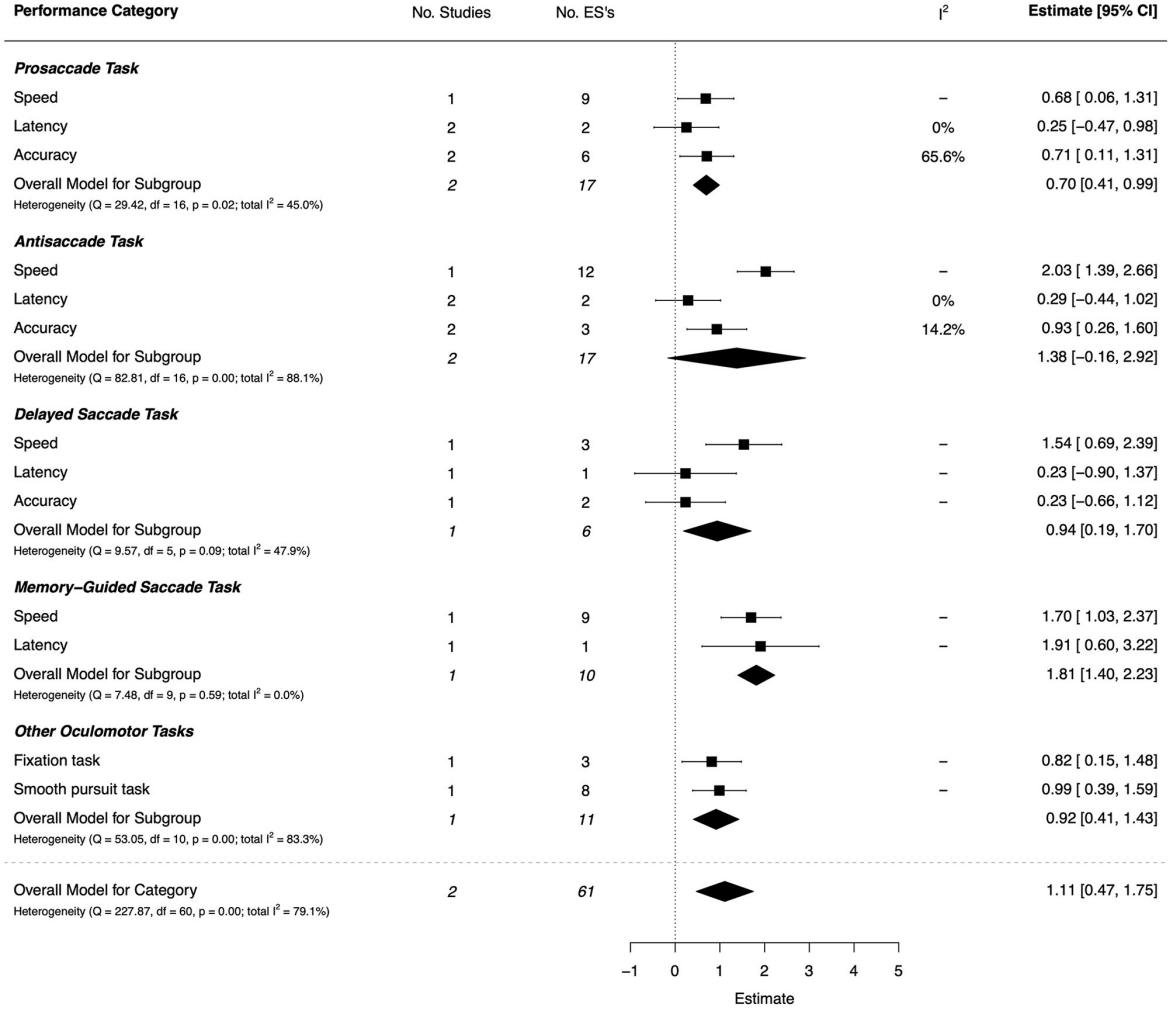
Forest plot showing the meta-analysis results for the oculomotor control performance category.

**Figure 8:**
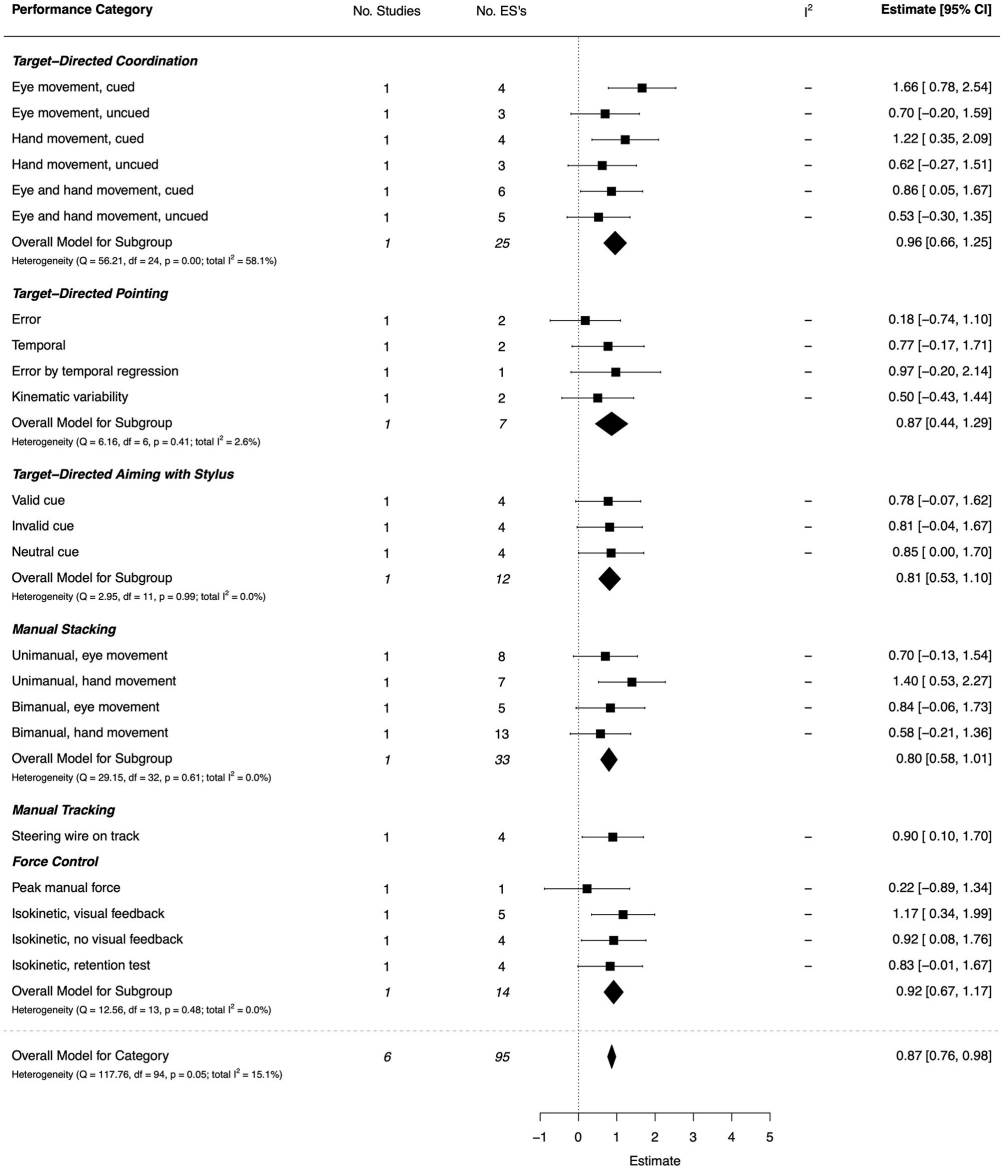
Forest plot showing the meta-analysis results for the reaching and manual control performance category.

**Figure 9:**
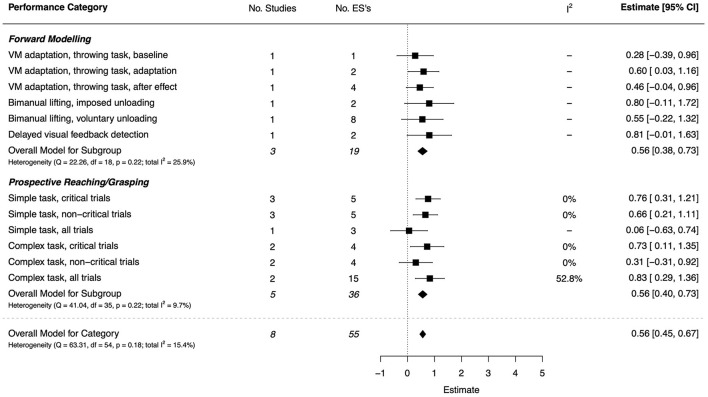
Forest plot showing the meta-analysis results for the internal modeling (incl. prospective reaching/grasping) performance category. VM, Visuomotor.

**Figure 10:**
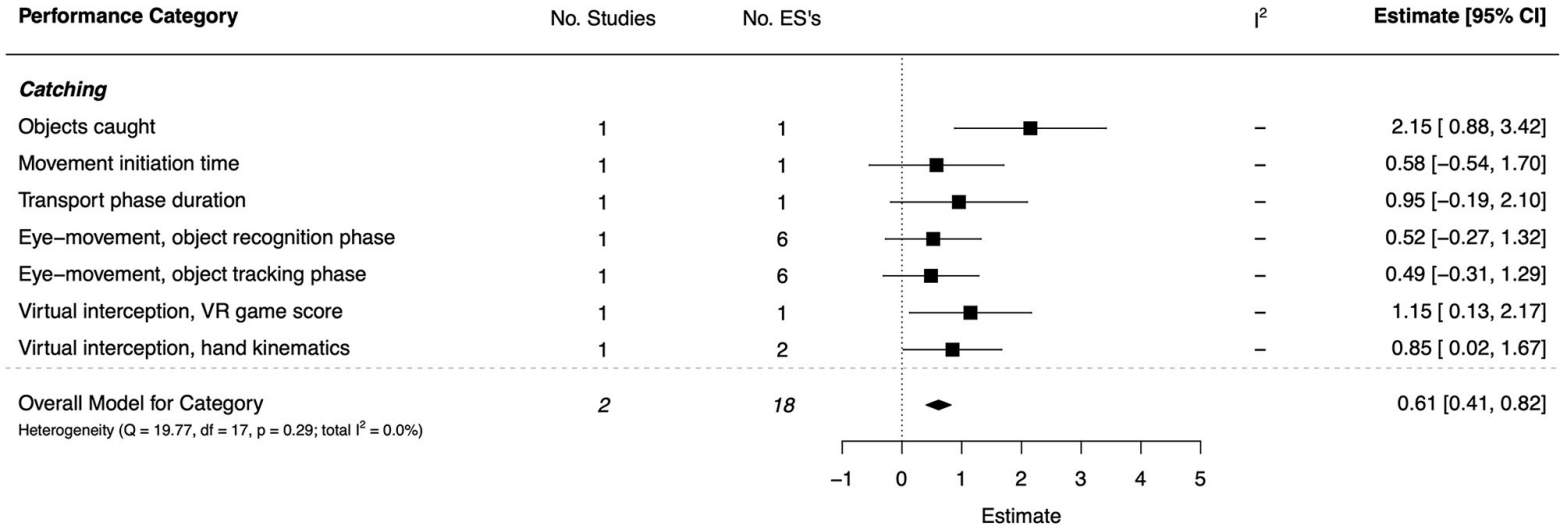
Forest plot showing the meta-analysis results for the catching performance category. VR, Virtual Reality.

**Figure 11:**
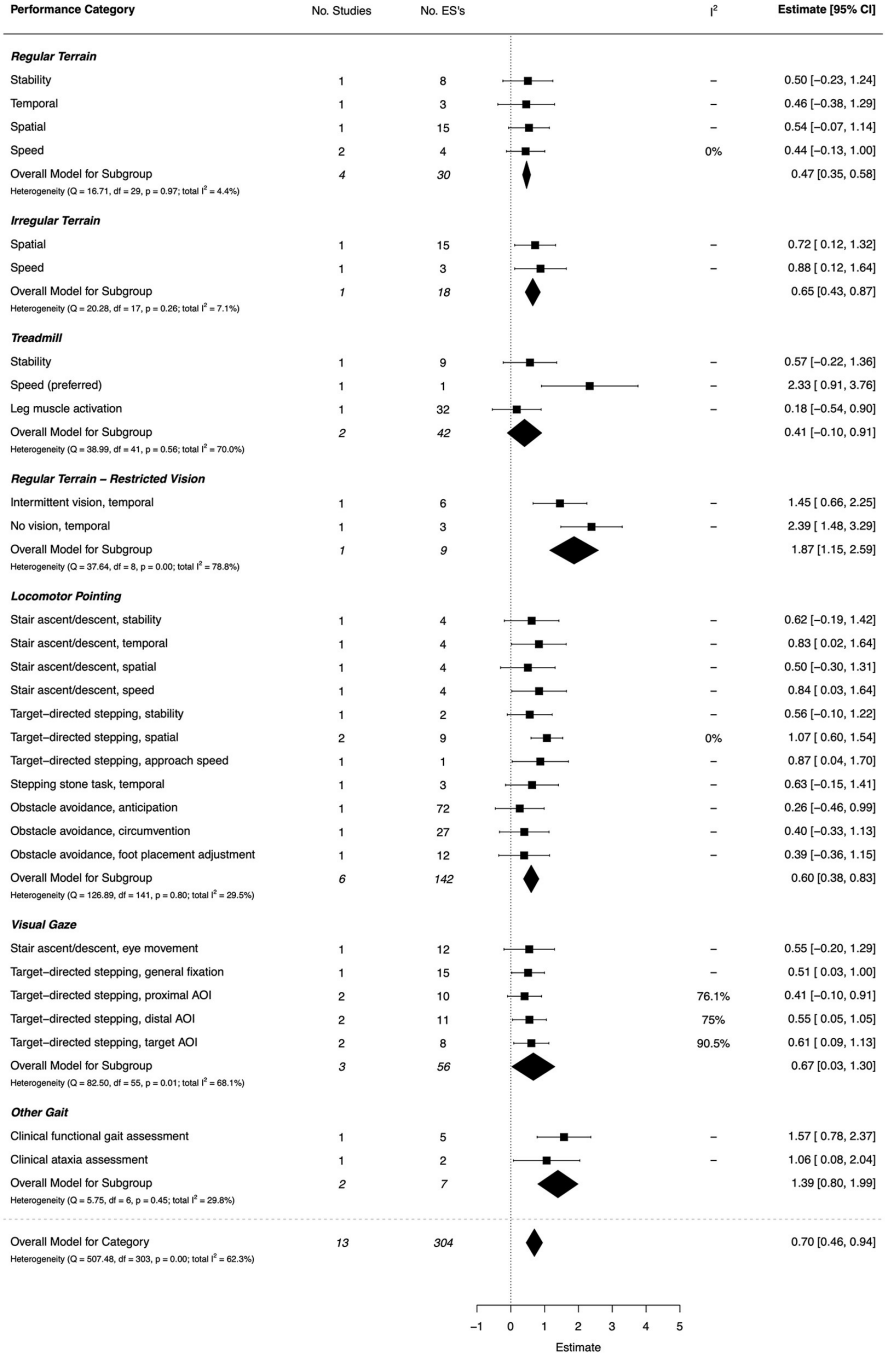
Forest plot showing the meta-analysis results for the gait (incl. visual control of gait and clinical assessment) performance category. AOI, Area-of-Interest.

**Figure 12:**
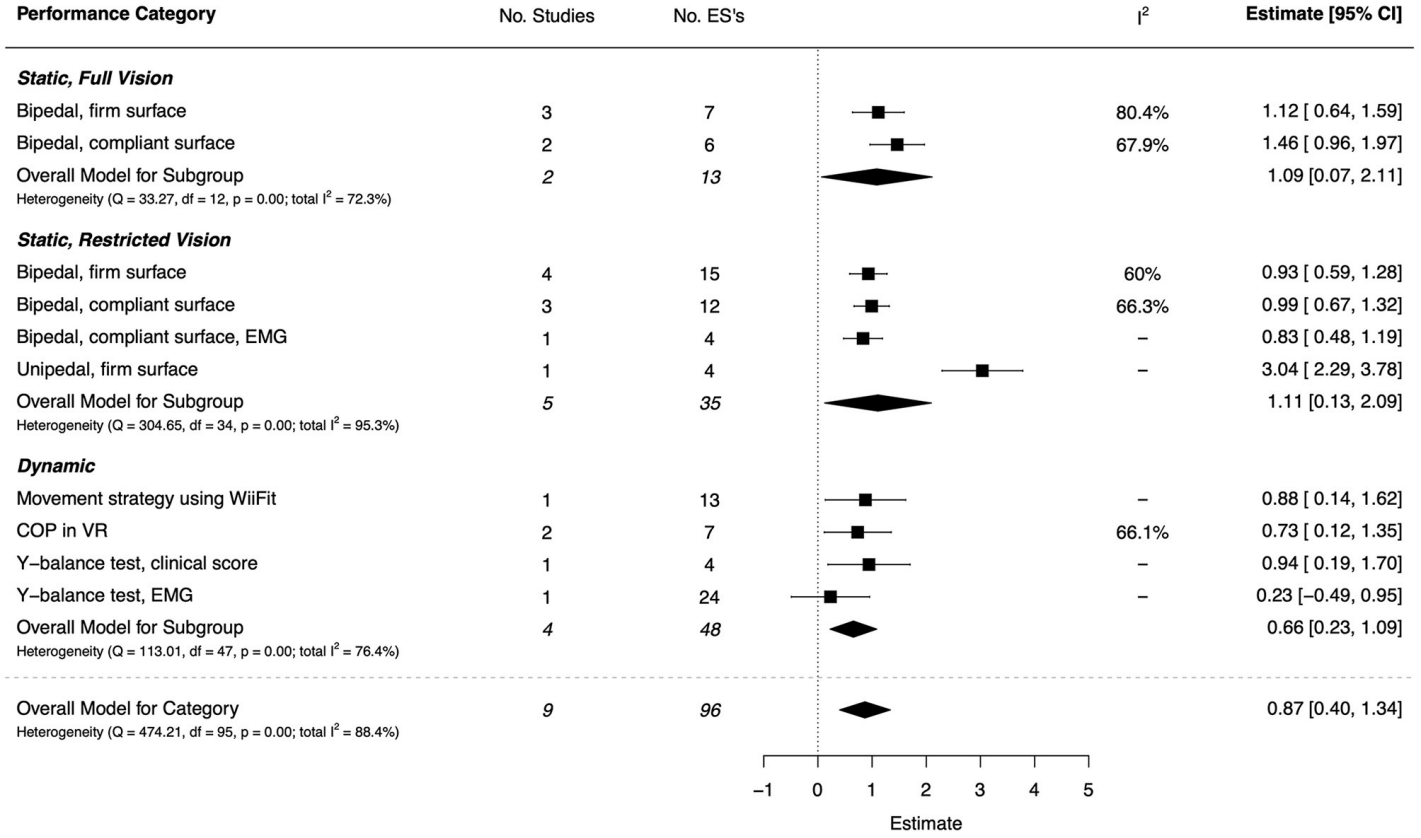
Forest plot showing the meta-analysis results for the postural control performance category. EMG, Electromyography; COP, Center-of-Pressure; VR, Virtual Reality.

**Figure 13:**
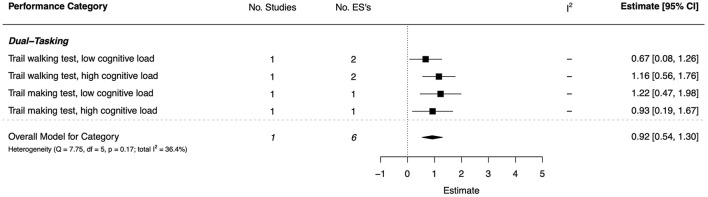
Forest plot showing the meta-analysis results for the dual-tasking performance category.

**Figure 14:**
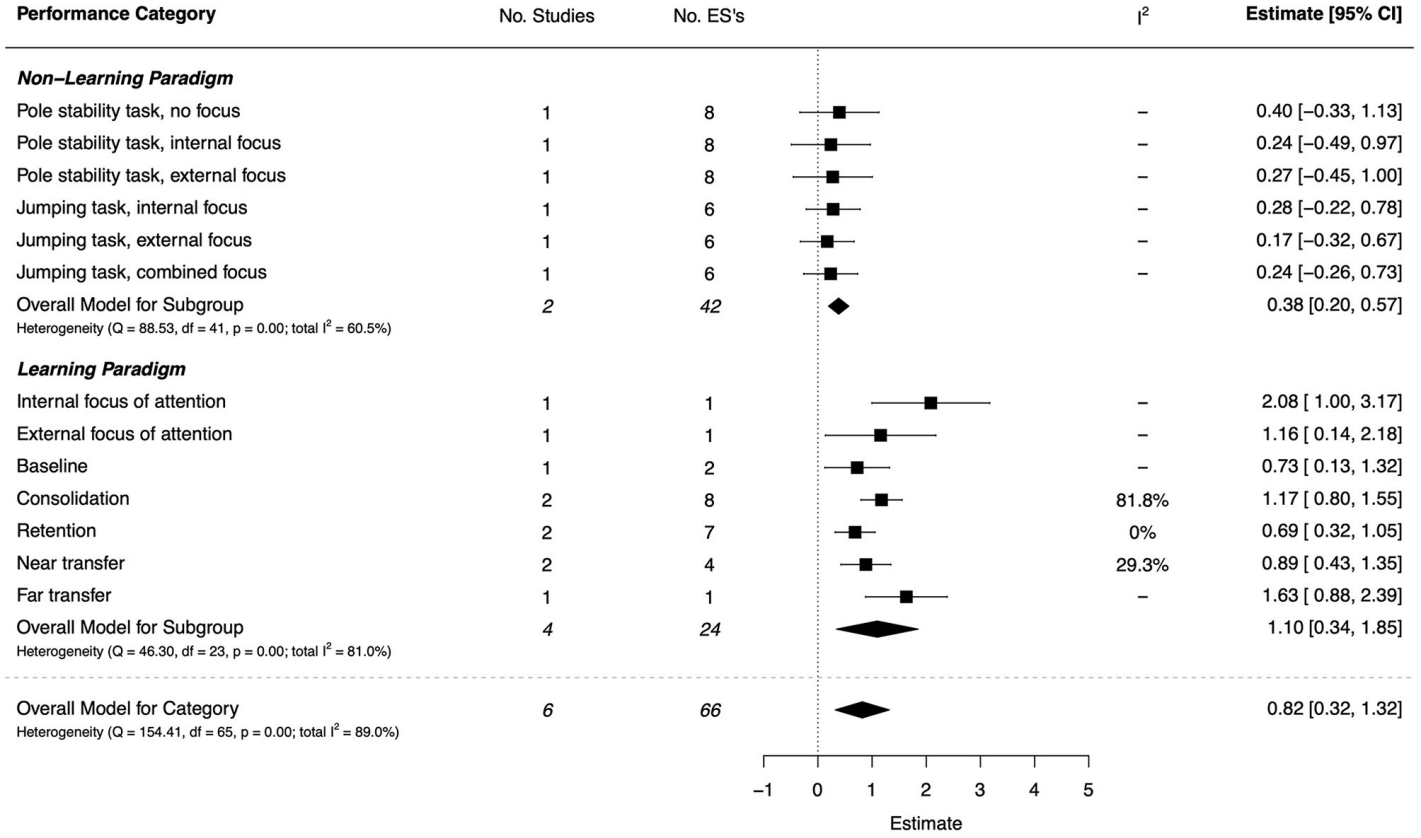
Forest plot showing the meta-analysis results for the attentional focus and motor learning performance category.

**Figure 15:**
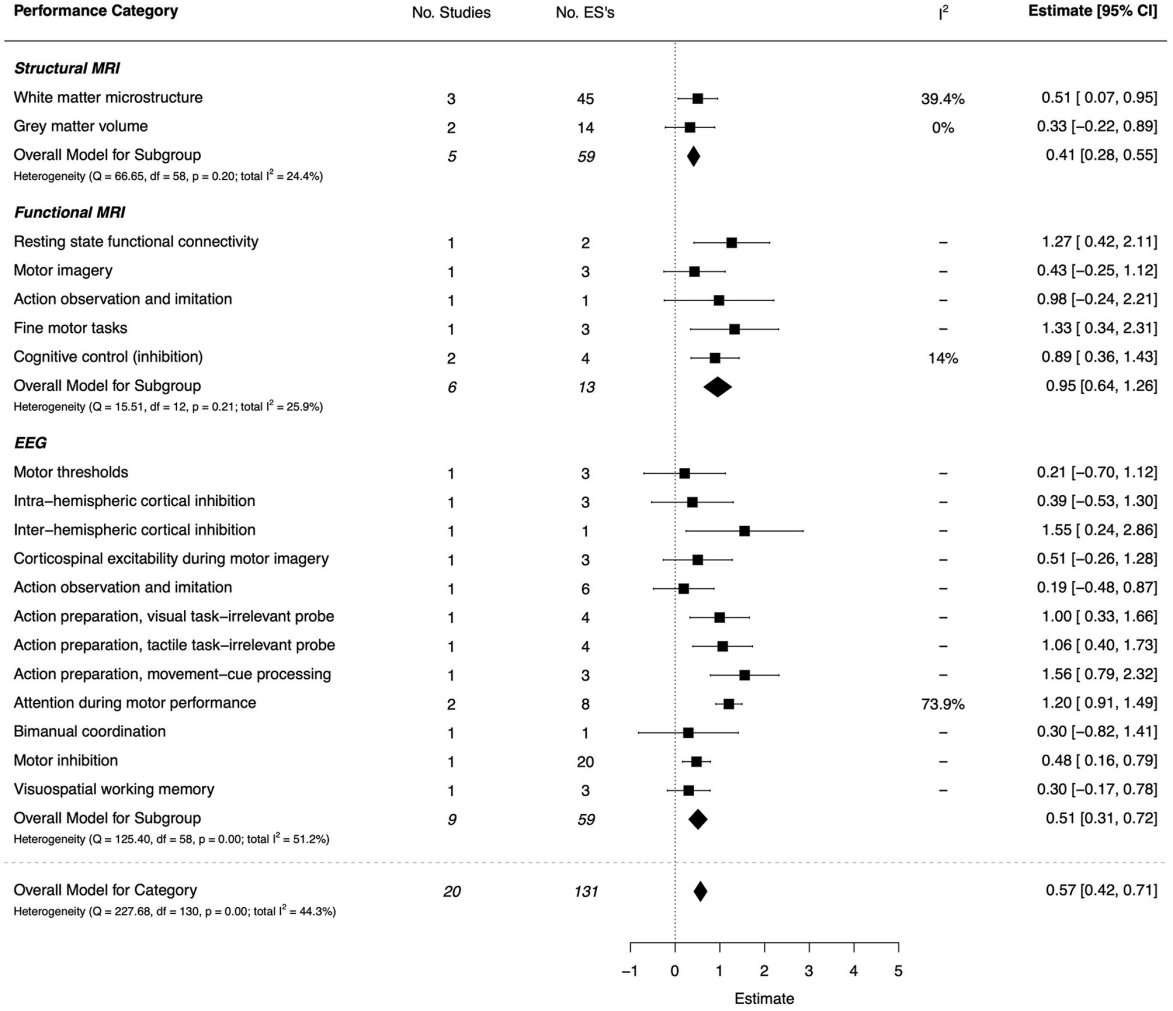
Forest plot showing the meta-analysis results for the neuroimaging performance category. MRI, magnetic resonance imaging; EEG, electroencephalography. EEG outcomes for a Serial Prediction task are not shown in the Forest Plot. The estimate [95% CI] for these tasks are: Visuomotor Task = 22.39 [17.76, 27.02], Perceptual Task = 2.79 [1.81, 3.77], Control Task = 15.04 [11.85, 18.23].

The PRISMA flow diagram in Figure 1 summarizes the number of available peer-reviewed articles at each phase of the systematic literature search. One hundred articles that reported an analysis of the underlying mechanisms of DCD (DCD-TD group comparison) were identified through the systematic search and then categorized by task domain for synthesis. The characteristics of these studies, including total study quality scores, are summarized in Supplementary Tables 1–13 of Supplementary Material 4. A risk of bias plot is presented in Figure 2, and a detailed breakdown for each CASP criteria is provided in Supplementary Material 5. The details of the combined ESs for each task performance and neuroimaging category are shown graphically in forest plots in Figures 3–15.

### Study Characteristics

The characteristics of the participants in DCD and TDC groups are reported in Supplementary Material 6. The median age of DCD and TD control samples was 9.8 and 10 years, respectively. The methods of participant sampling were described in varying levels of detail across studies; however, coding revealed a mixture of convenience sampling (75%), clinical referral (17%), and parent/teacher referral (1%), with 7% not clearly reported. With respect to motor screening tools, the Movement Assessment Battery for Children was used in 84% of studies, (MABC-2: 75% of studies; MABC 1^st^ edition: 9%). The next most used was the Bruininks-Oseretsky Test of Motor Proficiency (BOT-2) in 7% of studies, the McCarron Assessment of Neuromuscular Dysfunction (MAND) in 6%, and the Adult Dyspraxia Checklist (ADC) in 5%. Alongside the motor screening tools, parent report, ADHD screening, and intelligence scales were also used, but results not fully reported. A total of 76% of studies reported that DSM criteria for DCD were met, while 24% of studies did not meet DSM criteria. The severity of DCD could be estimated from 84 of the 100 studies based on the motor screening test cut-point criterion. Of these studies, 22% used a criterion that suggested *significant motor impairment* (≤5^th^ percentile), while 5% used the 10^th^ percentile, and 57% the 15^th^ percentile. *Borderline DCD* (or *at-risk*) is suggested for scores between the 6^th^ and 15^th^ percentile. Sixteen studies did not report specific cut points for motor screening tests. Regarding co-occurring disorders, 50% of studies reported that they excluded participants with intellectual disabilities, 41% reported excluding participants with co-occurring ADHD, and 18% excluded co-occurring ASD. A total of 27% of studies provided no information on the exclusion or otherwise of common co-occurring disorders.

### Methodological Quality Analysis

Based on CASP criteria, 64 studies (or 64%) were of high quality (8.9 ± 0.7), 23 moderate quality (7.0 ± 0.7), and 13 low quality (3.8 ± 0.9). Study attributes that were most commonly addressed in the sample of studies were: acknowledgment of theory (86%), clear identification of theory-driven question(s) (85%), relevance of task paradigm (85%), clear operationalisation and measurement (85%), appropriate use of statistical methods (62%), and valid implications in the discussion of results (84%). Areas of quality that were commonly “unclear” were: sufficient or justified sample sizes (44%), appropriate screening of DCD (31%) and control (42%) participants, and control of major confounds (52%).

### Meta-Analysis Showing the Magnitude of Group Differences in Each Performance Category

A total of 1,374 effect sizes were entered into the multi-level meta-analysis. The overall effect was moderate-to-large (*d* = 0.789, 95% CI = [0.688, 0.890]), indicating a generalized level of deficit in DCD groups relative TDC. A total of 212 moderators (at the level of performance sub-category) were entered into the model. For the model including all performance sub-categories, level-2 *I*^2^ = 8.54% and level-3 *I*^2^ = 55.23%, indicating that 63.77% of the total variance in the model was due to heterogeneity. More specifically, this shows that within-study variance was low, whereas there was moderate between-study variability in effect estimates (Higgins and Thompson, 2002). A summary of results according to each performance category are given below.

#### Sensori-Perceptual Function

There were 14 studies (13 high quality and 1 low) that contributed effect sizes in the sensori-perceptual task category, the magnitude of which was large overall (*d* = 0.86, 95% CI [0.48, 1.25]), but with substantial heterogeneity (*I*^2^ = 87.2%) (Ghotbi et al., 2016; Prunty et al., 2016; Johnston et al., 2017; Wang et al., 2017; Costini et al., 2018; de Waal et al., 2018; Tseng et al., 2018, 2019a,b; Alesi et al., 2019a; Chen et al., 2019, 2020; Adi-Japha and Brestel, 2020; Nobusako et al., 2021). The combined ESs are presented in Figure 3. A very large effect was found for visual perception of depth (*d* = 3.70, 95% CI = [2.65, 4.76])—the third largest of all task categories in the current analyses. Large effects were also found for visuo-spatial integration (*d* = 0.92, 95% CI = [0.27, 1.58]) and visuo-perceptual processing with motor involvement (*d* = 0.76, 95% CI = [0.50, 1.02]); the effect for object perception without motor involvement was small (*d* = 0.39, 95% CI = [0.08, 0.70]). A large effect was found for tactile and haptic perception (*d* = 0.83, 95% CI = [0.47, 1.19]), and moderate-to-large for kinaesthetic perception of distal-limb (*d* = 0.70, 95% CI = [0.31, 1.08]). For kinaesthetic perception of proximal-limb, however, the effect was negligible and non-significant (*d* = 0.06, 95% CI = [−0.35, 0.48]).

#### Executive Functions and Intelligence Factors

There were 21 studies (15 high quality, 4 moderate, and 2 low) that assessed aspects of executive function and intelligence, with a moderate overall effect (*d* = 0.49, 95% CI [0.40, 0.59]) (Rahimi-Golkhandan et al., 2016; Schott et al., 2016; Sumner et al., 2016; Adams et al., 2017b; Johnston et al., 2017; Mirabella et al., 2017; Wang et al., 2017; Bernardi et al., 2018; Costini et al., 2018; He et al., 2018a; Koch et al., 2018; Michel et al., 2018; Thornton et al., 2018; Alesi et al., 2019b; Barbacena et al., 2019; Job et al., 2019; Adi-Japha and Brestel, 2020; Gomez-Moya et al., 2020; Sartori et al., 2020; Suzuki et al., 2020; Wilson et al., 2020). The combined ESs are presented in Figure 4. Overall, significant, moderate-to-large effects were found for the main facets of executive function—inhibitory control, working memory, and executive attention—while effects were not significant for hot executive function. For inhibitory control, the strongest effects were observed for interference control (*d* = 0.80, 95% CI = [0.55, 1.04]) and action restraint (*d* = 0.67, 95% CI = [0.45, 0.89]), both on error/accuracy outcomes, as well as anti-jump performance (*d* = 0.63, 95% CI = [0.37, 0.89]). By comparison, temporal outcomes showed small-to-moderate effects, significant only for action restraint and interference control. For working memory, significant moderate-to-large effects were observed for visual (*d* = 0.79, 95% CI = [0.55, 1.02]) and verbal (*d* = 0.57, 95% CI = [0.26, 0.89]) processing. The same was also true of set shifting (*d* = 0.61, 95% CI = [0.39, 0.83]). For intelligence, moderate-to-large effects were shown for Full Scale IQ (*d* = 0.66, 95% CI = [0.27, 1.04]), Non-verbal IQ (*d* = 0.63, 95% CI = [0.24, 1.01]), and indices of Working Memory (*d* = 0.67, 95% CI = [0.12, 1.22]) and Processing Speed (*d* = 0.81, 95% CI = [0.25, 1.36]). For Verbal Comprehension and Perceptual Reasoning, the effects were small and non-significant.

#### Dynamical/Ecological Paradigms—Rhythmic Coordination and Ecological Perception

A total of six studies (four high quality and two moderate) were included in the dynamical/ecological domain, based on rhythmic coordination and ecological perception tasks, with a moderate overall effect (*d* = 0.65, 95% CI [0.38, 0.92]) (Roche et al., 2016; Blais et al., 2017, 2018; Purcell et al., 2017; Wilmut et al., 2017a; Lê et al., 2021). The combined ESs are presented in Figure 5. For rhythmic coordination, 17 of the 20 sub-categories showed small, non-significant effects across coupling, stability, and error outcomes. However, the exceptions were on visual-bimanual coupling (*d* = 0.73, 95% CI = [0.03, 1.42]), auditory-verbal sequencing errors (*d* = 0.88, 95% CI = [0.05, 1.70]), and auditory-visual-verbal sequencing stability (*d* = 1.07, 95% CI = [0.05, 2.10]) outcomes. For ecological perception tasks, the TD group were superior in the perception of temporal gaps (*d* = 1.17, 95% CI = [0.40, 1.93]) in a virtual road crossing task (Purcell et al., 2017). As well, a large, significant effect was found for perception-action judgement of aperture affordances (*d* = 0.90, 95% CI = [0.13, 1.67]), but effects were small and non-significant for visual estimation of affordances and the perception of action affordances for aperture traversing (Wilmut, 2017). A large number of comparisons (42) from a single paper contributed to the latter result.

#### Motor Imagery, Action Observation, Imitation, and Gesture Production

Both implicit and explicit MI were investigated in seven studies (six of high quality, and one moderate), with a moderate overall effect (*d* = 0.61, 95% CI [0.35, 0.88]) (Adams et al., 2017a,b, 2018; Kashuk et al., 2017; Fuchs and Cacola, 2018; Hyde et al., 2018; Scott et al., 2019). The combined ESs are presented in Figure 6. A large, significant effect was found for implicit MI (*d* = 0.81, 95% CI = [0.43, 1.19]). For explicit MI, the effect for objective tasks was moderate (*d* = 0.64, 95% CI = [−0.08, 1.37]) but non-significant, while for subjective tasks (involving self-report scales), the effect was small and non-significant (*d* = 0.39, 95% CI = [−0.22, 0.99]).

For the action observation (AO), imitation and gesture production category, there were nine papers (seven high quality, one moderate, and one low) (Reynolds et al., 2017a; Blais et al., 2018; Costini et al., 2018; Gauthier et al., 2018; Nobusako et al., 2018; Lust et al., 2019; Scott et al., 2019, 2020; Bieber et al., 2021). The effects of MI andAO(in isolation or combined) on motor imitation was investigated in six of these studies. Categories involving the production of meaningful gestures to verbal command or pictured object, imitation of complex novel gestures and object assembly had the largest, significant effects (*d* = 0.82–1.12). Observation and imitation of everyday actions showed large but non-significant effects, with a single entry per sub-category and wide CIs. Categories with the lowest and non-significant effects (ranging from *d* = 0.12–0.42) involved AO of simple gestures, and basic forms of imitation in the context of perspective taking and motor interference.

#### Oculomotor Control

The two included studies on oculomotor control (one high quality and one moderate) (Gaymard et al., 2017; Sumner et al., 2018) examined saccadic control, smooth pursuit, and fixation using conventional eye-movement paradigms. There was a very large overall effect (*d* = 1.11, 95% CI [0.47, 1.75]), but with substantial heterogeneity (*I*^2^ = 79.1%). The combined ESs are presented in Figure 7. For memory-guided saccades, there were very large effects on saccade latency (*d* = 1.91, 95% CI = [0.60, 3.22]) and speed of eye movement (*d* = 1.70, 95% CI = [1.03, 2.37]). Group effects were also very large for antisaccades on speed (*d* = 2.03, 95% CI = [1.39, 2.66]) and accuracy (*d* = 0.93, 95% CI = [0.26, 1.60]), and for delayed-saccade speed (*d* = 1.54, 95% CI = [0.69, 2.39]). For prosaccades, speed and accuracy were also reduced in DCD, but with moderate ESs. Other large ESs were observed on fixation time (*d* = 0.82, 95% CI = [0.15, 1.48]) and smooth pursuit performance (*d* = 0.99, 95% CI = [0.39, 1.59]). Finally, other aspects of latency showed small and non-significant effects.

#### Reaching and Manual Control

Goal-directed reaching was assessed in four studies (two high quality, two moderate) (Gama et al., 2016; Gonzalez et al., 2016; Golenia et al., 2018; Warlop et al., 2020a), manual tracking in one (Hsu et al., 2018), and force control in another (da Rocha Diz et al., 2018). There was a large overall effect (*d* = 0.87, 95% CI [0.76, 0.98]). The combined ESs are presented in Figure 8. Gonzalez et al.'s (2016) investigation of target-directed hand-eye coordination showed larger effects under cued conditions for eye (*d* = 1.66, 95% CI = [0.78, 2.54]) and hand movement (*d* = 1.22, 95% CI = [0.35, 2.09]) compared with uncued conditions (*d* = 0.70, 95% CI = [−0.20, 1.59] and *d* = 0.62, 95% CI = [−0.27,1.51], respectively). All effects of target-directed pointing were non-significant but primarily of moderate magnitude; these results were all reported from one study (Gama et al., 2016). The only significant effect for manual *stacking* was reported under unimanual, hand movement conditions (*d* = 1.40, 95% CI = [0.53, 2.27]), whereas bimanual conditions revealed moderate, but non-significant effects (Warlop et al., 2020a). A manual *tracking* (wire maze) assessment showed a large, significant effect (*d* = 0.90, 95% CI = [0.10, 1.70]). On force control tasks, large effects were shown under conditions of visual feedback (*d* = 1.17, 95% CI = [0.34, 1.99]) and when visual feedback was withdrawn (*d* = 0.92, 95% CI = [0.08, 1.76]) (da Rocha Diz et al., 2018).

#### Internal Modeling (Including Prospective Reaching/Grasping)

Aspects of internal modeling were examined in eight studies (seven high quality and one moderate) using a bimanual unloading paradigm (Cignetti et al., 2018), visuomotor adaptation paradigm involving target-directed ball throwing (Gomez-Moya et al., 2020), delayed visual-feedback detection in a hand-movement task (Nobusako et al., 2018), and end-state-comfort tasks (Adams et al., 2017a,b; Bhoyroo et al., 2018, 2019; Krajenbrink et al., 2021). There was a moderate overall effect (*d* = 0.56, 95% CI [0.45, 0.67]). The combined ESs are presented in Figure 9. The visuomotor task adaptation phase showed a significant, moderate ES (*d* = 0.60, 95% CI = [0.03, 1.16]), while the baseline phase and after-effect of adaptation revealed small, non-significant effects. Error-feedback detection during bimanual lifting showed moderate-to-large but non-significant effects (due to wide CIs).

For the prospective reaching and grasping category, most effects were moderate-to-large, with somewhat higher values for critical trials that required prediction of end state comfort (*d* = 0.76, 95% CI = [0.31, 1.21] and *d* = 0.73, 95% CI = [0.11, 1.35]). The two non-significant results were for non-critical trials (complex task) (*d* = 0.31, 95% CI = [−0.31, 0.92]) and a comparison on performance averaged over all simple trial types (*d* = 0.06, 95% CI = [−0.63, 0.74]).

#### Catching

For the catching category, two studies (one high quality and one moderate) focused on visual behavior during catching (Licari et al., 2018) and the interception of virtual objects (Wattad et al., 2020). There was a moderate overall effect (*d* = 0.61, 95% CI [0.41, 0.82]). The combined ESs are presented in Figure 10. Very large effects were revealed for task success, measured through the number of objects caught (*d* = 2.15, 95% CI = [0.88, 3.42]) and intercepted (*d* = 1.15, 95% CI = [0.13, 2.17]). Moderate-to-large effects were shown for other categories, but with wide CIs. On temporal aspects of performance, a moderate (but non-significant) effect was found for movement initiation time (*d* = 0.58, 95% CI = [−0.54, 1.70]) and a large (non-significant) effect for the duration of the transport phase (*d* = 0.95, 95% CI = [−0.19, 2.10]); only one group comparison contributed to each effect. Likewise, for gaze-related behavior, moderate effects were seen for the object recognition phase (e.g., number of fixations; *d* = 0.52, 95% CI = [−0.27, 1.32]) and tracking phase (e.g., time to smooth pursuit, number of blinks; *d* = 0.49, 95% CI = [−0.31, 1.29]). Finally, the effect for hand kinematics when intercepting virtual objects was large and significant (*d* = 0.85, 95% CI = [0.02, 1.67]).

#### Gait (Including Visual Control of Gait and Clinical Assessment)

Group differences on gait parameters were examined under different terrain and task conditions in 11 papers (all high quality): (i) overground walking on regular and irregular terrain (Gentle et al., 2016; Wilmut et al., 2017b; Nieto et al., 2018); (ii) treadmill walking (Speedtsberg et al., 2018; Yam and Fong, 2018); (iii) overground walking with intermittent and occluded vision (Nieto et al., 2018); (iv) stair negotiation (Parr et al., 2020b); and (v) locomotor pointing and obstacle avoidance tasks (Schott et al., 2016; Wilmut and Barnett, 2017a,b; Parr et al., 2020a; Warlop et al., 2020b). The overall effect for all gait outcomes was moderate-to-large (*d* = 0.70, 95% CI [0.46, 0.94]). The combined ESs are presented in Figure 11. A moderate effect was found for regular terrain gait outcomes (*d* = 0.47, 95% CI [0.35, 0.58]), however, each individual gait measure did not reach significance despite moderate effects. By comparison, on irregular surfaces, significant moderate-to-large effects were observed showing slower walking speed (*d* = 0.88, 95% CI = [0.12, 1.64]) and atypical spatial parameters (*d* = 0.72, 95% CI = [0.12, 1.32]) for DCD groups. Preferred walking speed on a treadmill was also significantly slower in DCD, with a very large effect (*d* = 2.33, 95% CI = [0.91, 3.76]). Altered temporal gait patterns under intermittent visual control (*d* = 1.45, 95% CI = [0.66, 2.25]) or occluded vision (*d* = 2.39, 95% CI = [1.48, 3.29]) was also shown.

During gait tasks that place an emphasis on accurate/targeted foot placements, such as stair negotiation and locomotor pointing tasks, children with DCD exhibited significantly slower approach speeds (each *d* ≥ 0.84) and larger and more variable spatial errors in foot placement (*d* = 1.07, 95% CI = [0.60, 1.54]). Furthermore, with respect to gaze behavior, there were moderate ESs showing different gaze patterns in DCD when surveying their surroundings (*d* = 0.51, 95% CI = [0.03, 1.00]); a greater proportion of time fixating the intended target during locomotor pointing (*d* = 0.61, 95% CI = [0.09, 1.13]), and less time fixating more distal areas of interest (*d* = 0.55, 95% CI = [0.05, 1.05]).

On clinical scales that assess the coordination of gait under different sensory conditions, studies showed that those with DCD have significantly poorer gait coordination than TDC peers (*d* = 1.57, 95% CI = [0.78, 2.37] on a functional index, and *d* = 1.06, 95% CI = [0.08, 2.04] on an ataxia index) (Mannini et al., 2017; Hsu et al., 2018). Across measures of gait pattern stability, independent studies showed a moderately reduced (but NS) level of stability in DCD for unconstrained walking (*d* = 0.50, 95% CI = [−0.30, 1.31]), treadmill walking (*d* = 0.57, 95% CI = [−0.22, 1.36]), stair negotiation (*d* = 0.62, 95% CI = [−0.19, 1.42]), and locomotor pointing tasks (*d* = 0.56, 95% CI = [−0.10, 1.22]).

#### Postural Control

Five studies (three of high quality, one moderate and one low) assessed differences in static postural control during unipedal and bipedal stance on both firm and compliant surfaces (Speedtsberg et al., 2017; Cheng et al., 2018; Nunzi et al., 2018; Chen et al., 2019; Ganapathy Sankar and Monisha, 2019). Very large effects were found for both full (*d* = 1.09, 95% CI [0.07, 2.11]) and restricted vision (*d* = 1.11, 95% CI [0.13, 2.09]), however there was substantial heterogeneity for both (*I*^2^ = 72.3 and 95.3%, respectively). The combined ESs are presented in Figure 12. Under all testing conditions, children with DCD exhibited significantly poorer outcomes than TDC for static postural control, regardless of whether vision was fully available, partially available, or completely obstructed (*d* ≥ 0.93). These group differences were most pronounced during unipedal stance when visual feedback was reduced or completely removed (*d* = 3.04, 95% CI = [2.29, 3.78]).

Four papers (two high quality and two low) assessed dynamic postural stability using traditional clinical assessments, interactive gaming methods, and immersive virtual reality environments (Miller et al., 2019; Yam and Fong, 2019; Jelsma et al., 2020; Wattad et al., 2020). For most measures of dynamic postural control, large and significant effects were observed in favor of TDC (each *d* ≥ 0.73); when trying to maintain equilibrium on tasks that required smooth and sequential movements of the center of gravity, children with DCD had larger excursions of COP and reduced stability, overall.

#### Dual-Tasking

For dual-tasking, there was one, high quality study that showed significant, and consistently moderate-to-large effects across locomotor-cognitive and manual-cognitive dual-tasks (*d* = 0.67–1.22) (Schott et al., 2016). The overall effect was large to very large (*d* = 0.92, 95% CI [0.54, 1.30]). The combined ESs are presented in Figure 13. The pattern of ES did not vary as a function of the type of motor task or cognitive load (low or high load).

#### Attentional Focus and Motor Learning

Two studies (both high quality) investigated the impact of attentional focus instructions on motor performance (Li et al., 2019; Psotta et al., 2020). The combined ESs are presented in Figure 14. Using experimental non-learning paradigms, motor performance was assessed on a pole stability task (Li et al., 2019) and a jumping task (Psotta et al., 2020). These tasks were completed under conditions of (i) internal focus, (ii) external focus, or (iii) no focus/combined focus instructions. Small, non-significant effects were found for all conditions, indicating no appreciable differences in performance between DCD and TD groups, regardless of the attentional focus used.

Motor learning under internal and external attentional focus conditions was assessed on a dart throwing task (Khatab et al., 2018). This study was low quality, however, the magnitude of the group effect for the internal focus condition (*d* = 2.08, 95% CI = [1.00, 3.17]) was almost double that of the external focus condition (*d* = 1.16, 95% CI = [0.14, 2.18]), suggesting that the DCD group benefited less from internal focus instructions.

The process of motor learning was also evaluated by three studies (two high quality, one low) using a (graphomotor) *invented letter task* (Adi-Japha and Brestel, 2020), Wii tennis and archery (de Carvalho et al., 2020), and Wii ski-slalom (Smits-Engelsman et al., 2020). All effects were significant and of moderate-to-large magnitude. The magnitude of the difference between DCD and TDC groups varied according to the phase of learning: large for consolidation (*d* = 1.17, 95% CI = [0.80, 1.55]), and moderate for retention of learning (*d* = 0.69, 95% CI = [0.32, 1.05]). In terms of skill transfer, larger group effects were seen for far transfer of learning (*d* = 1.63, 95% CI = [0.88, 2.39]) compared with near transfer (*d* = 0.89, 95% CI = [0.43, 1.35]).

#### Neuroimaging

##### Structural and Functional MRI

There were 11 included papers (two of high quality, four moderate, and five low) that used neuroimaging methods to investigate mechanisms of DCD (Kashuk et al., 2017; Reynolds et al., 2017b, 2019; Williams et al., 2017; Cacola et al., 2018; Koch et al., 2018; Thornton et al., 2018; Hyde et al., 2019; Brown-Lum et al., 2020; Rinat et al., 2020; Lê et al., 2021). Of these, MRI was used to explore structural differences in the gray matter volume (Reynolds et al., 2017b; Lê et al., 2021), white matter microstructure (Reynolds et al., 2017b; Williams et al., 2017; Hyde et al., 2018; Brown-Lum et al., 2020), and resting state functional connectivity (McLeod et al., 2016; Rinat et al., 2020). Two studies used fNIRS to examine differences in cortical activity between DCD and TDC groups (Cacola et al., 2018; Koch et al., 2018). The overall effect for structural MRI studies was small to moderate (*d* = 0.41, 95% CI [0.28, 0.55]), while the effect for functional MRI studies was very large (*d* = 0.95, 95% CI [0.64, 1.26]). The combined ESs are presented in Figure 15.

Differences in white matter structural connectivity (*d* = 0.51, 95% CI = [0.07, 0.95]) were evident at discrete sites of interest that included corticospinal projections (Hyde et al., 2018) and trans-callosal pathway. Conversely, for gray matter volume, no significant effect was shown between groups (*d* = 0.33, 95% CI = [−0.22, 0.89]). Functional neuroimaging revealed large, significant effects for resting state functional connectivity between the left motor cortex and structures of the basal ganglia (*d* = 1.27, 95% CI = [0.42, 2.11]), and decreased activation patterns within primary motor and sensory cortices during performance of fine-motor (*d* = 1.33, 95% CI = [0.34, 2.31]), and cognitive inhibition (*d* = 0.89, 95% CI = [0.36, 1.43]) tasks. For tasks involving motor simulation, results showed a large (but NS) effect on select neural sites that subserve AO and imitation (*d* = 0.98, 95% CI = [−0.24, 2.21]), and a moderate effect on sites associated with MI (*d* = 0.43, 95% CI = [−0.25, 1.12]).

##### Neurophysiological Investigation

Nine papers were included (four high quality, and five moderate) that used either EEG or transcranial magnetic stimulation (TMS) to investigate differences in brain function between DCD and TDC (Fong et al., 2016; Blais et al., 2017; Wang et al., 2017; Cheng et al., 2018; He et al., 2018b; Hyde et al., 2018; Job et al., 2019; Lust et al., 2019; Suzuki et al., 2020). The overall effect was moderate (*d* = 0.51, 95% CI [0.31, 0.72]). The combined ESs are presented in Figure 15. Mean effects ranged from small to very large across task categories (*d* = 0.19–1.56). Significant, large effects were shown for inter-hemispheric cortical inhibition (*d* = 1.55, 95% CI = [0.24, 2.86]), different aspects of action preparation (*d* = 1.00–1.56), and attention during motor performance (*d* = 1.20, 95% CI = [0.91, 1.49]); for motor inhibition the effect approached moderate size (*d* = 0.48, 95% CI = [0.16, 0.79]). Finally, very large and significant effects were shown for the EEG-Pz site under different conditions in a serial prediction task: perceptual (*d* = 2.79, 95% CI = [1.81, 3.77]), visuomotor (*d* = 22.39, 95% CI = [17.76, 27.02]), and control (*d* = 15.04, 95% CI = [11.85, 18.23]). The latter two (outlying) values are based on a single ES estimate, each (Opitz et al., 2020).

## Discussion

The aim of our combined systematic review and meta-analysis was to provide a thorough and rigorous synthesis of the recent body of experimental work on mechanisms of DCD, clarifying the profile of deficits across motor control, learning and cognition, and disruptions in brain structure and function. This was prompted by the proliferation of high-quality studies over the past 5 years. A total of 100 studies met inclusion criteria for the review, showing the continued growth in experimental work (Wilson et al., 2017b). Some 64% of included studies were rated as high quality, compared with 49% in the previous review that formed part of the international consensus statement on DCD (Wilson et al., 2017b). Aspects of study quality that were consistently evident was the development of theory-driven questions, paradigm validity, and logic of inferences drawn from results. Poorly addressed aspects were participant matching, sample size justification, use of strict DSM-5 criteria for DCD, and control of co-morbid conditions. The overall mean effect size from the current analysis (*d* = 0.79) was similar to that reported in the 1998 meta-analysis (*d* = 0.80) (Wilson and McKenzie, 1998) but somewhat smaller than that reported in 2013 (*d* = 0.97) (Wilson et al., 2013). In part, this trend may reflect the growing emphasis in recent years on publication of non-significant findings in the field of human movement and psychological science. Notwithstanding this, deficits of moderate-to-large magnitude were found in DCD across a wide range of performance categories suggesting the complex and interactive nature of motor control, learning, and cognition. The pattern of findings across categories suggests several emergent themes for an integrated theory of DCD, and for our understanding of mechanisms of motor control and cognitive-motor integration. The discussion that follows will focus on those themes.

### Emergent Themes for a Mechanistic Account of DCD

Returning to those hypotheses mentioned in the overview, we see evidence from a variety of paradigms that continue to provide (qualified) support to the IMD and associated MNS accounts, as well as converging behavioral and neuroimaging data that suggests atypical inter-hemispheric connectivity. However, we contend that several important themes emerge from the overall pattern of deficits we observe across performance categories, and their links to neuroimaging findings, both structural and functional. These themes cover visual-motor integration, cognitive-motor integration, learning, and motor variability (as well as potential neural underpinnings), discussed in turn below.

#### Visual-Motor Integration: A Fundamental Disruption in DCD

Well-designed studies in the current review (e.g., Gaymard et al., 2017; Sumner et al., 2018) and earlier work (Robert et al., 2014) indicate intact functioning in the control of basic (and more reflexive) eye movements, but also some defined deficits in more voluntary or controlled aspects of oculomotor control and atypical patterns of gaze behavior. Notably, a distinct difficulty was evident under non-visually guided conditions (e.g., antisaccade and memory-guided saccade tasks, *d* = 1.70–2.03) which require a higher degree of planning, inhibition, or shifting (i.e., cognitive control). An unusual saccade velocity profile—slower mean and maximum speeds, along with a relatively long deceleration phase (Gaymard et al., 2017), suggests a breakdown in visual-motor integration on these more cognitively demanding tasks (Koziol et al., 2012). This difficulty with visual-motor integration was also revealed during hand (*d* = 1.22) and eye (*d* = 1.66) movement in cued goal-directed reaching (Gonzalez et al., 2016), shown by gaze strategy variations during cup stacking (*d* = 0.70–0.84) (Warlop et al., 2020a), and in the visual control of gait whilst walking with an emphasis on accurate foot placement (*d* = 0.51–0.61) (Warlop et al., 2020b). Together, these various aspects of voluntary eye-movement control and gaze behavior highlight the important role of cognitive function in visual-motor integration, particularly in terms of motor planning and the predictive control of movement (i.e., internal forward modeling) (Deconinck et al., 2006; Wilson et al., 2013, 2017b; Adams et al., 2014). This integration problem is likely to contribute to the motor coordination difficulties observed in children with DCD, especially for time-constrained tasks that require precise coupling between oculomotor and limb kinematics.

These deficits in eye-limb coupling may also be constrained by difficulties in cognitive control and sustained attention in DCD (Bernardi et al., 2018; Michel et al., 2018). Vision is an active process, which involves anticipatory prediction and spatial attention to interpret visual stimuli (Gilbert and Li, 2013). Therefore, motor planning is conceptualized to arise through a top-down controlled process, which is underpinned by attention and visual-motor integration (Gilbert and Li, 2013). Taken together, varied gaze and visual behavior in DCD may reflect an area in which a breakdown occurs throughout the sensorimotor process. This notion is supported by current behavioral gaze research during locomotion (Warlop et al., 2020b) and cup stacking (Warlop et al., 2020a). During the unimanual cup stacking task, for example, those with DCD exhibited a preference for focusing their gaze within their immediate environment (or peri-personal space), displaying a higher number of fixations to guide their movement throughout the pick-up and stack process. Similarly, during locomotion on complex terrain that requires targeted foot placements, those with DCD shortened their gaze to the most imminent/proximal target, while their TD peers continued to look further along the path (Warlop et al., 2020b). We conclude that those with DCD are extracting visual information from a much higher number of fixations and shortened gaze targets to guide each aspect and phase of the movement. This pattern of control is likely designed to reduce uncertainty around task-relevant information, and to inform action choices that are not unduly difficult to implement by these children (Tong et al., 2017).

Neuroimaging data summarized in our review indicate that neural networks supporting “vision-for-action” (i.e., dorsal stream) are most affected in those with DCD, with evidence also of atypical structure and function in associated pathways that integrate “vision-for-perception” (i.e., ventral stream). Recent DTI research has revealed some evidence in adults with DCD of reduced white matter integrity within the superior longitudinal fasciculus (forming part of the dorsal stream) but relatively enhanced integrity within the inferior longitudinal fasciculus (ventral stream) (Williams et al., 2017). These findings may reveal a compensatory adaptation for DCD whereby the ventral stream assumes some of the visuomotor mapping functions of the dorsal stream (Williams et al., 2017). Abnormalities in the neural control of the early stages of motor planning have also been revealed using EEG. When using cues to direct the upper limb to unseen locations, recruitment of sensorimotor brain regions was reduced (*d* = 1.56), as evidenced by attenuated beta rhythms, which is understood to reflect motor preparation (Job et al., 2019). This finding in DCD is intriguing and suggests some difficulty representing movement to spaces that cannot be accessed immediately within the visual field. This hypothesis warrants further investigation.

#### Cognitive Deficits and Their Implications for Motor Planning and Control

Quite pervasive deficits of *executive function* were observed in DCD across inhibitory control, visual and verbal working memory, and executive attention (or set shifting) (*d* = 0.24–0.80). Moreover, task conditions that required efficient response inhibition and shifting in the context of a goal-directed movement were particularly problematic. This was evident, for example, in dual-tasking where large dual-task costs were reported across manual and locomotor tasks (*d* = 0.67–1.22) (Schott et al., 2016). Difficulty in dual-tasking is often linked to poor automatization of motor skills (Wilson et al., 2013, 2017b; Adams et al., 2014), which of course is almost synonymous with DCD. Interestingly, with aging, we also see issues in dual-task control as aspects of automatic and predictive control begin to wane (Zukowski et al., 2021). In effect, older adults tend to rely more heavily on slower, controlled aspects of motor planning, such that they enlist prefrontal structures to a greater extent under dual-tasks conditions (Li et al., 2018). Given what we know about poor predictive control in DCD, this raises the hypothesis that children with DCD are faced with the dual challenge of relying more heavily on slower, voluntary, and feedback-based aspects of movement control while also having some limitations in the capacity of EF systems that support this mode of control. Put another way, reduced automatization of motor skills (and poor predictive control) in DCD engenders a more energy-intensive approach to motor planning and control, thereby reducing the capacity to share cognitive networks when a secondary task is imposed.

From a neural perspective, fMRI data on resting state and tasks that require fine-motor and cognitive control (Kashuk et al., 2017) has revealed a reduced pattern of activation across parietal-frontal and parietal-cerebellar networks in adults with DCD (Kashuk et al., 2017). As well, DTI has shown reduced white-matter connectivity within the parietofrontal network, evidenced by reduced fractional anisotropy (FA) (Williams et al., 2017). These neural correlates are consistent with the conclusions of Adams et al. (2014) about a possible parietal-cerebellar disconnection in DCD (Tallet and Wilson, 2020). This network is involved in the generation of predictive (forward) models and comparison of predictive estimates with external sensory feedback. As well, maturation of the “super-highway” that bridges processing between anterior and posterior structures—the superior longitudinal fasciculus—may also be disrupted. In summary, there is enough converging behavioral and neuroimaging data to suggest that motor-cognitive integration is impaired in DCD, and that further testing of this hypothesis is required.

#### Learning Under Specific Conditions of Practice

A key finding from the learning research (see Section *Attentional Focus and Motor Learning)* was that those with DCD have a capacity to learn motor skills under relatively simple task conditions. Indeed, the rate of learning in some studies was comparable to TDC, while the absolute level of performance remained worse (*d* = 0.69–1.63). However, to approximate the level of performance of TD peers, DCD groups benefitted from adapted learning parameters including clear instructions for effective focus of attention (Khatab et al., 2018), double the learning time (de Carvalho et al., 2020), and augmented visual feedback (Smits-Engelsman et al., 2020) throughout the learning process.

From a neural perspective, impaired functioning of the MNS may explain some of the differences in motor learning capacity between DCD and TD. The most fundamental means by which children learn goal-directed motor skills is through observation (Latash and Lestienne, 2006), seen in varying degrees in the learning paradigms reviewed here. Recent EEG-based analysis of AO in children with DCD has revealed reduced mu suppression and lower mu coherence between frontal and parietal regions; by comparison, coherence increased during pause (or non-action) intervals (Lust et al., 2019). These results suggest that those with DCD are less able to integrate information about action goals, conveyed through observation which is a critical part of MNS function. Rather, these children may need to use pause intervals in order to fully process action goals and the means to achieve them. Therefore, altered MNS function in DCD may dictate that learning conditions that we consider normal may not, in fact, be sufficiently rich in information to support their skill development.

It is important to note that not all neuroimaging studies reveal aberrations in MNS activation. For example, using a simple finger tapping task, the fMRI study of Reynolds et al. (2019) showed no group effects in relation to the MNS. Rather, differential activation patterns were seen in networks associated with motor planning and attention including the caudate body, thalamus, and posterior cingulate. The degree of complexity in the topography (and kinematics) of movement may explain these inconsistencies across studies. Tasks that require mapping of more complex goals to movement kinematics are more likely to enlist the complete architecture of the MNS. Under such conditions, atypical neural and behavioral function is likely in those with DCD.

The upshot for learning is that children with DCD may require adapted learning conditions to acquire and refine their motor skills to a level that approaches their TD peers. Our understanding of the learning process in DCD will benefit greatly from studies that combine behavioral and neuroimaging techniques (like EEG and fNIRS) to track changes in neuromotor function over time—i.e., changes over different phases of learning, and as a function of training intensity and different informational constraints.

#### Motor Variability—The Good, the Bad, and the Ugly

An important recurring issue in DCD research, amplified in the current review, concerns motor variability—viz the “*good vs. bad variability”* debate. Over multiple studies of gait and reaching, the motor performance of children with DCD was much more variable, expressed in its topography, kinematics, and kinetics. This was shown by slower and more cautious gait patterns, evidenced by smaller step length, reduced velocity, and increased sway (see Section Gait) on irregular surfaces (Gentle et al., 2016; Nieto et al., 2018; Speedtsberg et al., 2018; Parr et al., 2020b). These characteristics of movement may be seen as adaptations to minimize the destabilizing momentum of locomotion and to allow additional time to pick up the environmental information necessary to reduce uncertainty (and risks) about the path ahead (Tong et al., 2017).

Further, those with DCD were characterized by slower and more variable reaching and pointing movements, yet similar reaching errors to TD (Gama et al., 2016; Golenia et al., 2018) (see Section Reaching and Manual Control). More specifically, for reaching there was evidence of greater variability in DCD for those joint angles that do *not* affect finger/endpoint position—explored by Golenia et al. (2018) using the *uncontrolled manifold* (UCM) method. As such, not all aspects of variability are negative in performance terms. Rather than being considered strictly as a deficit (i.e., “bad variability”), this increased variability could be conceptualized as an adaptive method (i.e., “good variability”) (Latash et al., 2007), whereby those with DCD increase degrees of freedom to explore more of the *action space* in order to (a) derive a reasonable action solution under increasingly complex environmental and motor demands, and (b) maintain their safety throughout the process.

One intriguing hypothesis is that children with DCD may actually *learn* to walk (and reach) slower and more cautiously than is typical, using this as a strategy to compensate for more fundamental deficits in motor control (including cognitive-motor integration). In effect, this strategy would help preserve safety margins when tackling new environments and skills, and reduce injury risk, consistent with data that we reviewed on walking through apertures (Wilmut and Barnett, 2017a; Wilmut et al., 2017a) and stair climbing (Parr et al., 2020b). Similarly, in the literature on aging, compensatory behavior is seen to occur as a response to functional deterioration. Older adults tend to enlist a more controlled mode of response when dealing with complex tasks and environments, involving hyperactivation of prefrontal and other cortical regions, and heavy reliance on feedback-based motor corrections. This mode of response can be understood as a method to compensate for age-related decline in the physical integrity of the motor plan, thereby reducing the risk of injury (Poirier et al., 2021).

The slow-controlled mode of adaptation is not always optimal, however. For tasks that need to be performed rapidly and under high visuospatial uncertainty, slower and more variable responses may come with a cost. For example, when negotiating moving cars while crossing a road, slower and more variable movements are unlikely to be functional but rather unsafe. Indeed, Purcell et al. (2017) showed that the crossing judgements of children with DCD increased collision rates when (virtual) cars approached at slower speeds, unlike TD peers.

Finally, it is important to understand the learning history of children with DCD and the types of opportunity afforded them to practice and learn motor skills. That is, movement compensations in these children will also reflect the way their parents and other caregivers have scaffolded their learning environments. We know that skill learning can be a frustrating, difficult, and sometimes painful process for many children with DCD. Avoidant or overly cautious patterns of behavior are not uncommon and can be reinforced by adults (Bringolf-Isler et al., 2018). In some cases, it may simply be easier to take most of the “hard work” out of new task that the child is struggling to learn, e.g., carrying an infant over a potholed pavement, rather than letting them find a way through it, leaving out important practice time. An ecological approach to the issue of both movement variability and, more specifically, learned compensations is recommended to unpack the interactive effects of individual maturation, task constraints, and environmental conditions (Wilson et al., 2017a).

### Comorbidities

Clinical comorbidities (ADHD, visual problems like strabismus, behavioral problems, pre-term birth, and executive dysfunction) continue to be an important consideration when interpreting the results of experimental work on DCD (esp. learning and training studies) and divining their implications for theory and practice (Visser, 2003; Biotteau et al., 2016; Dewey and Bernier, 2016). In some cases, comorbid groups were compared with DCD alone which enabled hypotheses about the specificity of mechanisms in neurodevelopmental disorders to be tested (e.g., Cignetti et al., 2018). For example, the fMRI study of Thornton et al. (2018) showed reduced activation across motor and sensory cortices in children with co-occurring DCD+ADHD compared with DCD or ADHD alone. In other studies (fortunately a minority), no explicit inclusion/exclusion criteria were provided about comorbid conditions like ADHD, ASD, Developmental Dyslexia and visual problems. Clearly, unrecognized comorbidity introduces a level of heterogeneity in the expression of “DCD,” error in the measurement of performance, and perhaps greater variability on our key metrics—this then reduces our level of confidence in the inferences drawn. We recommend that to progress our understanding of the core mechanisms of DCD, comorbidities need to be clearly reconciled, either by exclusion from samples, or by their planned inclusion as part of a theory-driven hypothesis.

### Future Directions

Fertile areas for future research concern the expansion of longitudinal methods (esp. those using microgenetic approaches), and methods to unpack mechanisms of cognitive-motor integration. The former involves repeated and frequent sampling of behaviors of interest across time periods that are known or believed to capture significant change in developmental processes. However, investigation of control parameters as drivers of developmental change in DCD remains a challenge when, (a) longitudinal research is rare, (b) there are few age-related comparisons in experimental studies of DCD, and (c) adult samples make up almost half of the recent neuroimaging studies. Indeed, only one study in the current review tracked longitudinal changes in children with DCD (Adams et al., 2017a), revealing a developmental delay in MI ability, and *catch-up phase* over a 2-year year period. Only one behavioral study compared the performance of (younger and older) children with DCD with age-matched peers and adults (Wilmut and Barnett, 2017b). Studies of this type provide a powerful method for testing hypotheses about *developmental delay* in DCD, the moderating effects of activity and participation (Imms et al., 2017), and patterns of performance into adolescence and early adulthood.

A discrepancy in the age distribution of DCD samples was noted between the behavioral and neuroimaging research. Unsurprisingly, children were the prime focus in behavioral research, with the exception of three studies using adults only (Job et al., 2019; Warlop et al., 2020a,b) and two comparing children with adults (Wilmut and Barnett, 2017b; Khatab et al., 2018). Of the neuroimaging work, six studies involved adults only (Hodgson and Hudson, 2017; Kashuk et al., 2017; Williams et al., 2017; He et al., 2018a,b; Hyde et al., 2018). Among other things, adults with DCD often report more difficulty with EF and cognitive self-regulation relative to motor coordination (Purcell et al., 2015). As well, there are probable distinctions in the performance of adults (who have persisting DCD) compared with those sampled in childhood, some of whom will go on to develop persistent DCD and others remitting DCD (Wilson et al., 2020). Distinctions between these different developmental pathways is a question of great interest to research.

We argue that understanding the mechanisms of cognitive-motor integration in children with DCD is critical to the advancement of theory and potentially also diagnostic and intervention frameworks. Among areas of potential focus is the performance of dual-tasks that, by definition, require integration of motor and cognitive control, and that become more important to functional behavior with age. The current review included only one such paper, showing significantly greater cognitive-motor interference in DCD across locomotor and manual tasks (Schott et al., 2016). Giving added impetus to this line of work are the pervasive deficits in executive function that we observed in DCD (see also Wilson et al., 2020).

### Limitations

While capturing a large sample of studies (98) conducted since the most recent consensus review (Wilson et al., 2017b), our meta-analysis obviously did not include the body of work published prior to September 2016. To temper this limitation, we have discussed the pattern of findings presented here in relation to the body of work that formed part of earlier systematic reviews (Wilson et al., 2017b) and meta-analyses (Wilson and McKenzie, 1998; Wilson et al., 2013).

A number of performance categories showed significant heterogeneity in ESs and, in most cases, this could not be resolved for statistical or conceptual reasons. Again, we accept the final results of the meta-analysis under the caveat that heterogeneity (for some categories) does reflect an element of unresolved error in the combined ES estimate.

### Clinical Implications

There are several important clinical implications for practitioners that emanate from our review. First, continued progression in evidence has been provided for DCD and its comorbidity with other neurodevelopmental disorders and, perhaps, “executive dysfunction.” It remains important for practitioners to evaluate co-occurrence throughout the diagnostic and intervention process.

Second, difficulties in EF and cognitive-motor integration have been highlighted profoundly in our review. This cluster of deficits emphasize the relatively poor integration of cognition and movement in the performance of children with DCD, and stresses the importance of addressing this within assessment, rehabilitation, and educational settings. In short, the presentation and experience of DCD goes well-beyond the motor system—practitioners and researchers alike need to consider assessment of, and support for, associated cognitive and attentional challenges. Earlier work on children with *Deficits in Attention, Motor control. and Perceptual abilities* (DAMP) (Gillberg, 2003) has shown the common co-occurrence of motor and attentional issues in DCD, but some of the lessons of this longitudinal work has been forgotten or ignored. The implications of DCD combined with executive dysfunction are profound for academic learning and achievement, psychosocial adjustment, and wellbeing (Gillberg and Kadesjö, 2003; Zwicker et al., 2013; Kirby et al., 2014). As such, well-designed (and early) adjustments in the classroom and at home are recommended; even simple adjustments can foster development, safety, and well-being (Sylvestre et al., 2013; Wilmut and Purcell, 2020; Zwicker and Lee, 2021).

Third, the learning and attentional focus literature, in particular, has highlighted the potential of principled methods of skill instruction and practice. Use of clear instructions, combination of AO+MI, guided learning through cuing to help direct attentional focus, augmented visual feedback, and extended periods of learning are among the most important techniques available for those with DCD (Smits-Engelsman and Wilson, 2013). Further, although there are important questions that still require detailed investigation, a combination of activity-oriented approaches focussing on task-specific skills, active computer games, and group-based interventions all show promise for the improvement of motor performance in DCD (Smits-Engelsman et al., 2018). The attendant gains in skill, however small, have a reinforcing effect on the motivation of children and their willingness to persist in training. Interestingly, recent evidence shows that gains in motor performance following a Cognitive Orientation to Occupational Performance (CO-OP) intervention are accompanied by a sustained increase in white matter microstructure and functional connectivity between brain networks associated with emotion regulation, action inhibition, and attention for those with DCD (but not DCD+ADHD) (Izadi-Najafabadi and Zwicker, 2021; Izadi-Najafabadi et al., 2021). Together, the available evidence provides encouraging support for science-led intervention.

## Conclusion

Children (and adults) with DCD show a generalized pattern of deficit across outcome measures, of at least moderate magnitude in effect-size terms. However, areas of more profound deficit were noted in the following: voluntary control of gaze when reaching or walking, cognitive-motor integration and its neural underpinnings, motor learning that is more contingent on practice type and intensity, predictive motor control (or internal modeling), more variable movement kinematics/kinetics, and higher safety margins when locomoting, especially when negotiating obstacles or gaps; the latter is likely to be a pragmatic compensation for more basic motor control deficits that impact traversing and reaching. Importantly, the review identified several unifying themes across these areas of deficit: first, a fundamental breakdown in visual-motor integration that impacts performance of eye-hand coordination and locomotor navigation (particularly over irregular terrain); second, difficulties in cognitive control and its integration with motor planning; third, potential decomposition of the heightened motor variability in DCD into “good” and “bad” components. These themes are an excellent departure point for continued growth in our theoretical understanding of DCD and those mechanisms that deserve particular focus in future research.

## Data Availability Statement

The original contributions presented in the study are included in the article/Supplementary Material, further inquiries can be directed to the corresponding author/s.

## Author Contributions

ES-Z, MC, TM, and PW: conceptualization, methodology, and project administration. ES-Z, TM, and PW: formal analysis. MC, TM, and PW: supervision. TM: visualization. All authors: data curation, investigation, drafting, review and editing.

## Funding

While completing this research, ES-Z was supported by an Australian Government Research Training Program Scholarship. Completion of this study and manuscript was supported by funding awarded to PW under the Research Centre scheme, Australian Catholic University, and the Czech Science Foundation (GACR EXPRO scheme: 21-15728X). ED is supported by a grant from the Knut and Alice Wallenberg Foundation (KAW 2020.0200). The funding bodies had no influence in the study design, in the collection, analysis, and interpretation of data, in the writing of the report, or in the decision to submit the article for publication.

## Conflict of Interest

The authors declare that the research was conducted in the absence of any commercial or financial relationships that could be construed as a potential conflict of interest.

## Publisher's Note

All claims expressed in this article are solely those of the authors and do not necessarily represent those of their affiliated organizations, or those of the publisher, the editors and the reviewers. Any product that may be evaluated in this article, or claim that may be made by its manufacturer, is not guaranteed or endorsed by the publisher.
