# Supplementary material for: Behavioral and Neuroimaging Research on Developmental Coordination Disorder (DCD): A Combined Systematic Review and Meta-Analysis of Recent Findings
Source: Front Psychol. 2022 Jan 27;13:809455. doi: 10.3389/fpsyg.2022.809455 (PMC8829815; doi:10.3389/fpsyg.2022.809455)
Supplement: Supplementary file 1 [file Data_Sheet_1.zip › Supplementary Material - Data Sheet 1/Supplementary Material 4.docx]

**Supplementary Material 4**

Systematic Review Tables

Table of Contents

[Table 1. Sensori-perceptual function 2](#_Toc78968423)

[Table 2. Executive functions and intelligence factors 9](#_Toc78968424)

[Table 3. Dynamical / Ecological Paradigms 16](#_Toc78968425)

[Table 4. Motor Imagery, Action Observation, Imitation and Gesture Production 21](#_Toc78968426)

[Table 5. Oculomotor Control 26](#_Toc78968427)

[Table 6. Reaching and Manual Control 28](#_Toc78968428)

[Table 7. Internal modelling (incl. Prospective Reaching/Grasping) 32](#_Toc78968429)

[Table 8. Catching 36](#_Toc78968430)

[Table 9. Gait (incl. Visual Control of Gait and Clinical Assessment) 38](#_Toc78968431)

[Table 10. Postural Control 45](#_Toc78968432)

[Table 11. Dual-Tasking 50](#_Toc78968433)

[Table 12. Attentional Focus and Motor Learning 51](#_Toc78968434)

[Table 13. Neuroimaging 55](#_Toc78968435)

******Data from the papers are often relevant across multiple domains, however, for the review tables, each paper is listed in* ***one*** *domain of best fit.*

# **Table 1.** *Sensori-perceptual function*

| **Reference** | **Study Objectives** | **Participants**  *Age and/or Gender Matched* | **Referral method**  *School*  *Community*  *Clinical* | **DCD Screening Tool, Cut-Off.** | **Co-occurring disorders in sample**  *Y/N/Not Reported*  *(NR)* | **DSM**  **Met**  Y/N | **Task(s) & Outcome measures** | **Key Results** (* significant) | **Effect Sizes** (key group effects) | **Quality Score**  (/10) | **Main Findings** |
| --- | --- | --- | --- | --- | --- | --- | --- | --- | --- | --- | --- |
| (Cacola et al., 2018) | Investigate cortical activation using fNIRS in DCD. | N = 6 DCD  N = 6 TD  8-12Y | Clinic and community | 1. MABC-2 <5th %tile (DCD)  2. DCDQ – daily functioning  3. Delay in motor milestones  4. No other medical conditions  5. KBIT-2 >Average | ID: NR  ADHD: NR  ASD: NR | Y | Cortical activation during fine motor task:  Finger tapping, curve tracing and paragraph writing while fNIRS measured cortical activation  (t-test comparison to baseline) | Motor performance  DCD<TD.  Cortical activation sig different between DCD and TD. | Not available | 5 | - Neurological alterations in children with DCD  - Different activation patterns, even for FT tasks which require low fine motor control and no spatial or temporal demands |
| (Chen et al., 2019) | Compare changes in light touch sensitivity in children with DCD and the influence on body sway. | N=26 DCD  N= 26 TDC | School | **DCD**  1. MABC-2 <5th %tile  **TDC**  1. MABC-2 >50th %tile  **BOTH**  2. K-BIT >80  3. Connors TRS <70  No injuries/conditions  Complete right-handedness (+100)  Normal vision | ID: NR  ADHD: N  ASD: NR | N | Tactile perception task:  A variety of touch conditions  (3x60s trials) after immersion in surfactant-water solution.  Sensitivity measured through force exerted on the touch plate. | Initial sensitivity:  DCD > TD*  Body sway before:  DCD > TD*  Body sway after solution  DCD=TD* | Initial sensitivity:  d = 1.087  Body Sway  before:  d = 1.765  Body sway after solution:  d = 0.282 | 10 | - DCD exhibit impaired light tough (LT) sensitivity compared to TD.  - Finger soaking improves LT sensitivity in both DCD and TD and reduces body sway only for DCD. |
| (Chen et al., 2020) | Determine lower extremity joint position sense and its relationship with motor function in DCD. | N= 28 DCD  N= 28 TD  Range 9-12 years | School | **DCD**  1. MABC-2 <15^th^ %tile  2. Daily living interference: MABC-2 checklist >95^th^ %tile  TD  1. MABC-2 >15^th^ %tile  2. MABC-2 <85^th^ %tile  BOTH  1. CTRS <70  2. KBIT-2 >80  3. No history of injuries/conditions | ID: NR  ADHD: N  ASD: NR | Y | **Kinaesthetic perception task:**  Execution of passive ipsilateral joint position match tests while seated on a biodex isokinetic dynamometer.  Position error (PE) and position error variability (PEV) was calculated. | PE and PEV both knee and ankle joints  DCD > TD* | PE Ankle:  *d* = 2.765  PE Knee:  *d* = 0.760  PEV Ankle: *d* = 2.660  PEV Knee *d* =0.738 | 8 | DCD exhibit:  - Inferior proprioceptive acuity (PE and PEV) at knee and ankle joints.  - Poor proprioceptive acuity at ankle joint compared to knee joint  - Negative correlations between impaired joint position sense and lower extremities and balance function |
| (Costini et al., 2018) | Nature and specificity of gestural deficits in children with DCD and their relation to cognitive skills | N = 30 DCD  N = 30 TD  7-13Y | Clinical DCD  School TD | **DCD:**  1. Confirmed diagnosis of DCD based on MD ax  2. Verbal IQ >70 | ID: N  ADHD: NR  ASD: NR | Y | Praxis functioning assessment: adapted for gestural assessment in children.  Accuracy % correct. | Deficits in most gesture production tasks and impaired visuospatial skills in DCD. | Representational gesture production:  d = 1.176  Non-representational gesture production:  d = 0.827 | 10 | - No evidence for a deficit in the conceptual system, semantic knowledge, or sensorimotor knowledge.  - DCD impaired for both representational transitive and nonrepresentational gestures: indicative of a production system level deficit |
| (de Waal et al., 2018) | Examined association between DCD and academic performance | N = 174 TD  N = 47 DCD  10Y | School | **BOTH:**  1. MABC-2: Mod-Sev DCD <5-16^th^ %tile and >16^th^ %tile TD  2. VMI-4 | ID: NR  ADHD: NR  ASD: NR | Y | MABC-2, VMI-4 and academic school grading  Measure scores and academic achievement across 6 learning areas | Visual perceptual coordination skills (VMI-4)  DCD<TD* | VMI-MC: *d* = 0.400  MABC-2 total:  *d* = 2.510 | 8.5 | - Sig inferior visual-perceptual coordination skills in DCD  - Negative influence on mathematic academic performance with lower visual perception and manual dexterity skills  - Visual perception highly correlated with mathematical achievement in children with and without DCD |
| (Ghotbi et al., 2016) | Compare depth perception of children with and without DCD | N = 40 DCD  N = 40 TD  7-9years | School | **BOTH**  1. MABC-2  2. Snellen Visual Acuity Test  3. Visual health or corrected vision | ID: NR  ADHD: NR  ASD: NR |  | Depth perception D9009 model evaluated 3 times  Average error of depth perception | Average error  DCD>TD* | Average Error:  *d* = 3.703 | 5 | - Weaker motor functions and motor delay in DCD negatively impacts visual perception of depth |
| (Johnston et al., 2017) | Investigate external spatial representations of touch in children with DCD | N = 19 DCD  N = 35 TD  7-11years  Matched with either age or cog ability | Community | **DCD:**  1. DSM-5  2. MABC Checklist  Score >95th %tile  3. MABC-2 test score <16th %tile  4. Connors 3  5. >70 on abb IQ tests  **TD:**  1. MABC <95th %tile  2. Absence of conditions | ID: NR  ADHD: N  ASD: NR | Y | Simplified tactile localization task to identify which hands a vibrotactile stimulus ‘tickled’ was presented to  % of tactile localisation accuracy | Tactile localization: Overall accuracy  DCD<TD*  Cross hands effect  DCD=TD | Overall accuracy:  d = 0.776 | 9 | - Tactile localization (TL) impairments in relation to age expectations for DCD  - Immature TL ability in DCD and external frame of reference used for localizing touch  - Both groups exhibit poor TL accuracy when hands are crossed and indicates reliance on external frame of reference for coding touch |
| (Nobusako et al., 2021) | To investigate perceptual biases of children with DCD. | N = 19 DCD  N = 19 TD  Age and sex matched. | School | **BOTH:**  1. MABC-2: <16th DCD and >50th %tile TD.  2. DCDQ  3. Onset in early development  4. No medical/cog conditions  5. No prior diagnoses of ASD, ADHD, Learning disorders | ID: N  ADHD: N  ASD: N | Y | Visual-tactile temporal order judgement (TOJ) task: measure and compare perceptual biases.  Point of subjective equality (PSE): calculation of perceptual bias. Large neg PSE = strong visual bias. Large pos PSE = strong tactile bias.  PSE close to 0ms = no perceptual bias | DCD>TD*  Strength of visual bias (PSE)  Visual bias correlated* with poor manual dexterity. | PSE  d = 0.910 | 9 | - Strong visual bias in DCD which was associated with poor manual dexterity.  - High visual dependence in children with DCD.  - Enhanced visual bias may be explained by atypical brain function (decreased inferior parietal activity) in DCD. |
| (Prunty et al., 2016) | Examine the existence of an association between visual perception and visual motor integration for handwriting skills in DCD. | N = 28 DCD  N = 28 TD | Community | **DCD:**  1. MABC <10th %tile  2. MABC Checklist  3. Dev/med history  4. BPVS-2 – Lack of ID  5. SDQ for hyperactivity  **TD:**  1. MABC-2  2. BPVS-2  3. BAS-II  All SS >85 | ID: N  ADHD: N  ASD: N | Y | Developmental Test of Visual Motor Integration (VMI) and Test of Visual Perceptual Skills (TVPS)  Test scores | VMI & TVPS  DCD<TD*  Correlation between VMI and Visual perception and group  DCD=TD | VMI Score  d = 1.160  TVPS Score  d = 1.250 | 10 | - Visual perceptual skills were poorer in children with DCD  - Neither of the measures related to the handwriting product/process difficulties in DCD  - Other factors may explain handwriting difficulties in DCD |
| (Tseng et al., 2018) | Examine wrist position sense and relationship between fine and motor balance function in DCD and TD | N = 20 DCD  N = 30 TD  10-11YEARS | School | **BOTH:**  1. MABC - <5^th^ %tile DCD -25^th^ %tile TD  2. MABC Checklist  3. Medical information  4. Exclusion criterion – medical/conditions and IQ<85 | ID: N  ADHD: NR  ASD: NR |  | Wrist on a lever for the completion of ipsilateral wrist joint position matching. This required active movement and psychophysical passive rotation.  Error variability for joint position (PE) in degrees | Position sense:  Error  Error variability* Just-noticeable difference*  DCD>TD | Error: *d* = 0.102  EV: *d* = 0.710  JND: *d* = 1.160 | 9.5 | - Proprioceptive dysfunction of the wrist/hand is associated with DCD and may contribute to motor problems  - Limb position sense is affected in children with DCD  - Motor interference may play a role in perceptual deficits in DCD (seen as less precise or delayed). |
| (Tseng et al., 2019) | Examine whether haptic perception is impaired in DCD | N = 20 DCD  N = 20 TD | School | **BOTH:**  1. MABC-2 – DCD <5th %tile and TD >25th %tile  2. Medical reports for exclusion and IQ >85  3. Edinburgh handedness inventory | ID: N  ADHD: NR  ASD: NR | Y | Curvature detection task – moved index finger of dominant hand over surface to determine curved contours  Ability to perceive the contour of a curved surface – (Haptic sensitivity) and ability to discriminate between curved surfaces (acuity) | Haptic perception (acuity)  DCD<TD*  Haptic detection threshold  (sensitivity)  DCD>TD | Acuity: d = 0.850  Sensitivity: d = 0.084 | 8.5 | - DCD may be associated with impaired haptic perception which contributes to observable fine motor deficits  - Specific deficits in haptic acuity is a common feature of DCD which may contribute to fine motor abnormalities |
| (Tseng, Tsai, et al., 2019a) | Assess position sense acuity at the elbow and wrist in DCD and TD | N = 20 DCD  N = 30 TD  9-11 years | Unclear | **BOTH:**  1. MABC-2 <5^th^ %tile DCD and >25^th^ %tile TD  2. Info, medical reports and IQ >85 | ID: N  ADHD: NR  ASD: NR | N | Children sat on chair with wrist on a lever for completion of ipsilateral and contralateral matching.  Position error (PE) – position sense bias  Position sense error variability (SDPE) – position sense precision | Position sense precision at wrist and elbow  DCD<TD* | Main effect of group: Wrist: *η_p_ ^2^* = 0.19  Elbow: *η_p_ ^2^* = 0.08 | 9 | - Position sense precision lower at the wrist than the elbow in both groups  - No clear evidence that PE is larger in DCD  - PE deficits can be present at proximal and distal arm joints in DCD: which increase random error  - Generalised proprioceptive dysfunction in DCD characterised by > in sensory precision. |

# **Table 2.** *Executive functions and intelligence factors*

| **Reference** | **Study Objectives** | **Participants**  *Age and/or Gender Matched* | **Referral method**  *School*  *Community*  *Clinical* | **DCD Screening Tool, Cut-Off.** | **Co-occurring disorders in sample**  *Y/N/Not Reported*  *(NR)* | **DSM**  **Met**  Y/N | **Task(s) & Outcome measures** | **Key Results** (* significant) | **Effect Sizes** (key group effects) | **Quality Score**  (/10) | **Main Findings** |
| --- | --- | --- | --- | --- | --- | --- | --- | --- | --- | --- | --- |
| (Alesi et al., 2019) | Determine literacy readiness for kindergarten children at risk for DCD and TD. | N = 13 arDCD  N = 13 TD | School | **BOTH:**  1. MABC-2: TD >16^th^ %tile and DCD >15^th^ %tile  2. Parent self-report  3. Absence of ID and medical impairment | ID: N  ADHD: NR  ASD: NR | Y | **Executive function task:**  MABC-2 and Pre-literacy skills measure where children need to rapidly name sequence of objects  Measure total scores | Visual memory and discrimination.  **Errors**  arDCD>TD  Visual fluency.  **Time**  arDCD>TD | **V memory:**  *(Task b) d* = 2.232  **V discrim:**  *d* = 3.983  **Visual fluency:** *d* = 0.620 | 9 | - Deficits identified in pre-literacy domain for at risk of DCD  - Effects of DCD are evidenced to extend to cognitive systems |
| (Alesi et al., 2019a) | Compare EF in kindergarten children at risk for DCD to TD. | N = 18 arDCD  N = 18 TD  Age matched | School | **BOTH:**  1. MABC-2: DCD <15th %tile and TD >16TH %tile  2. Parent self-report measures  3. No ID, neuro-dev disorder | ID: N  ADHD: N  ASD: N | Y | Standardised EF task battery: 3 cold EF: Working memory, fluency, and inhibitory control  2 hot EF: gift wrap and snack delay.  4 x Trial scores combined | Cold EF task performance  arDCD<TD*  Hot EF task performance  arDCD=TD | WM:  d = 1.209  Fluency global:  d = 1.596  Inhibitory control:  d = 1.249 | 8 | - Differences in working memory for children at risk of DCD  - No differences identified in Hot EF tasks  - Extent of difficulties extend further than just motor systems and cognitive systems may also be impaired |
| (Barbacena et al., 2019) | Investigate relationship between cognitive level and motor performance of children with/without DCD | N = 35 DCD  (25 Severe DCD)  N = 367 TD  7-10y | School | **BOTH:**  1. MABC-2 <15th %tile DCD and >16th %tile TD  2. DCD-Q  3. No diagnosed medical, neurological | ID: NR  ADHD: NR  ASD: NR | Y | *Cognitive profile:*  *RAVEN progressive matrix test score*  *Motor performance:*  *MABC-2 scores* | Cognitive profiles  DCD=TD  Motor performance and cognitive levels* in DCD. | d = 0.551 | 7.5 | - No association identified between presence of DCD and cognitive level  - Reinforces heterogeneous DCD profile |
| (Bernardi et al., 2018) | Investigates the development of executive functions in children with poor motor skills. | N = 17 DCD  N = 17 TD  7-11y | Clinical | **BOTH:**  1. MABC-2 <16th %tile MD(DCD) and >25th TD  2. MABC-2 checklist | ID: NR  ADHD: NR  ASD: NR | Y | EF battery: verbal and nonverbal measures: Non-verbal Executive Loaded Working Memory (ELWM)  Measure scores | NV ELWM total correct time 0 and 1  DCD<TD* | T0  d = 1.161  T1  d = 0.949 | 8 | - DCD performed poorer than TD at all time points for nonverbal measures of EF  - DCD demonstrated EF difficulties which persisted over 2 years  - Gap in EF between DCD and TD remains stable during middle childhood |
| (He et al., 2018) | Evaluate the inhibitory deficit hypothesis of DCD. | N = 11 DCD  N = 11 TD  18-30Y  Age matched | University and social media | 1. ADC: DCD >95% CI  2. BOT-2: DCD <15th %tile and TD <20th %tile | ID: NR  ADHD: NR  ASD: NR | Y | Go/No-Go to assess prepotent action and Stop-signal task to assess ability to cancel prepared movement.  Action restraint efficiency index: Mean AREI  Action cancellation stop signal time: Length of SSRTs | AREI scores:  DCD < TD*  SSRTs:  DCD > TD | AREI scores:  d = 0.987  SSRTs:  d = 0.892 | 7.5 | - Broad difficulties identified in engaging inhibitory mechanisms during motor behaviour  - First account of action cancellation difficulties along with action restraint challenges in DCD |
| (Job et al., 2019) | Investigate movement preparation and sensory processing consequences for adults with pDCD compared to TD. | N = 12 pDCD  N = 12 TD  Adults  22-29Y  IQ Matched | Clinical and community | **pDCD:**  1. DCD diagnosis in childhood or adulthood  2. Adult DCD checklist >65  **TD:**  1. Adult DCD checklist outside ‘at risk’ range | ID: NR  ADHD: NR  ASD: NR | N | Delayed movement task:  Cued reaching towards peri personal and unseen personal space. EEG recording  Tactile processing and sensory information use | Personal Mov RT  pDCD > TD  Interaction between group and movement preparation*  pDCD<TD | Personal Mov RT  d = 0.349  Interaction  ηp 2 = 0.159 | 7.5 | - pDCD exhibit differences in neural mechanisms of spatial selection and action preparation compared to TD  - Selective enhancement of visual processing was identified by pDCD towards the goal location within peripersonal space  - DCD may be impaired at using somatosensory signals during movement preparation and underly their over-reliance on vision. |
| (Ke et al., 2019) | Evaluates specific cognitive deficits in Chinese children with DCD. | N = 75 DCD  N = 291 TD  7-10Y | School | **BOTH:**  1. MABC-2 <5 DCD, >TD | ID: NR  ADHD: NR  ASD: NR | N | *MABC and Cognitive ability assessment:*  *attention, memory, reactivity, and executive function (EF).*  *(Global test score)* | All EF scores  DCD<TD* | Global EF score:  d = 0.475 | 7 | - Cognitive deficits in DCD centre around poor EF rather than attention and memory difficulties  - Children’s movement development may have an impact on their memory, attention, EF, and other cognitive abilities |
| (Michel et al., 2018) | Cognitive and motor coordination skills of children with and without motor difficulties. | N = 48 TD  N = 48 MD  4-6 years  Gender and age matched | School | **BOTH:**  1. MABC-2 <10^th^ %tile MD group  2. Physical fitness tasks (KTK) | ID: NR  ADHD: NR  ASD: NR | N | MABC-2 scores and cognitive tasks. *Inhibition:* go-no-go discrim  Index. *Switching:* Incongruent mixed flanker trials. *WM:* corsi-blocks backwards longest sequence recalled. | All measures of EF at T0 and T1  _persistent_DCD<TD  *Correlation between EF and motor coordination in _persistent_DCD. | Inhibition:  T0 *d* = 0.876  T1 *d* = 0.521  Switching: T0 *d* = 0.428 T1 *d* = 0.221  WM: T0 *d* = 0.503 T1 *d* = 0.772 | 8 | - Highlight differences in the cognitive profile overtime and weaker visuospatial static and dynamic WM for DCD. |
| (Mirabella et al., 2017) | Compare processing of action-verbs in children with DCD | N = 18 TD  N = 18 DCD | Clinical | **BOTH:**  1. Prior DCD diagnosis  2. MABC-2 <15th %tile  3. DCQ: suspect or indicative DCD  4. FSIQ >80 WISC-V  5. PPVT >85 | ID: N  ADHD: NR  ASD: NR | Y | Online verb version of a go/no-go task.  Mean reaction times (RTs) and movement times (MTs) of correct trials | % errors on semantic tasks:  DCD > TD*  Reaction time changes when semantic context must be retrieved for TD only not DCD. | % errors on semantic tasks:  Range d = 0.684 – 1.099 | 3 | - Language processing is impeded in DCD |
| (Rahimi-Golkhandan et al., 2016) | Examine deficits in hot (emotionally rewarding) EFs in children with DCD. | N = 12 DCD  N = 24 TD  7-12Y | School | 1. > motor skill that interferes with day-to-day activity  2. MD <85 and TD >100 score on NDI or MAND 3. No ID, medical conditions, ASD, ADHD | ID: N  ADHD: N  ASD: N | Y | *Respond to computer based motional go/no-go task of facial expressions.*  *Omission errors and RT to go and commission errors to no-go target.* | Speed of RTs to sad faces  DCD<TD  Happy no-go faces commission errors  DCD>TD | Sad G:  O Errors d = 0.508 RTs  d = 0.727*  Happy NG: C Errors d = 0.807 | 9.5 | - Pattern of DCD performance confirms deficits in Hot EFs  - Reduced ability in DCD to inhibit response to emotionally rewarding cues.  - More difficult for DCD to modulate their approach tendencies to emotionally significant stimuli. |
| (Sartori et al., 2020) | Compare EFs in children with DCD, rDCD and TD. | N = 63 DCD  N = 63 TD  8-9years  Sex, age, grade, and SES matched. | School | **ALL:**  1. MABC-2: %tile <5th DCD, 5-<16th rDCD, >16th TD  2. MABC-2 checklist DCD delays  3. WASI: DCD >79 | ID: N  ADHD: NR  ASD: NR | Y | Children completed verbal working memory, inhibitory control, and cognitive flexibility tests.  M (SD) of test scores | Nonverbal  WM: Odd-one-out*.  Inhibitory control (IC) – Hayling test errors*.  Cognitive flexibility (CF) TMT  DCD<TD | NV WM d = 1.054  IC: d = 1.356  CF:  d = 0.409 | 10 | - Lower performance on several components of EFs in DCD  - Evidence of similarities in EF components regardless of severity of motor impairment (rDCD or DCD) |
| (Sumner et al., 2016) | Characterise the IQ profile of children with and without DCD. | N = 52 DCD  N = 52 TD  Age and gender matched | School | **DCD:**  1. Prior diagnosis by clinician  2. MABC-2 <10th %tile  3. Daily living deficits  4. No medical, ADHD, ASDH conditions  **TD:** 1. MABC-2 >25th %tile. | ID: NR  ADHD: N  ASD: N | Y | *Completed the WISC-IV.*  *M(SD) for test indices and FSIQ*  *- VCI, PRI, WMI, PSI* | Total index scores:  Processing speed (PSI)  Working memory (WMI)  DCD<TD*  Subtest performance: Digit span and coding  DCD<TD* | PSI: d = 0.930  WMI d = 0.480  Digit span: d = 0.567  Coding: d = 0.943 | 10 | - Weaker working memory and processing speed performance like ASD/ADHD IQ performance  - Heterogeneous cognitive profile in DCD.  - Heavy motor demands for processing speed tasks questions the validity of this index for the DCD population. |
| (Suzuki et al., 2020) | Examine influence of motor clumsiness of DCD on Go/Nogo task. | N = 25 DCD  N = 56 TD  Age, sex & handedness matcheds | Community | **DCD:**  1. MABC-2 total score <7  **TD:**  1. MABC-2 total score >7 | ID: NR  ADHD: NR  ASD: NR | N | Go/Nogo N2 and N3 Task completed under varied difficulty levels assessed by EEGs.  Commission error rate (CER)as an indication of motor clumsiness | CER for rare Nogo condition  DCD>TD  Correct RT and CER  DCD=TD | CER:  d = 0.548 | 6 | - DCD display difficulties stopping the motor response  - Impaired imitation of actual motor response and subsequent cognitive process delay in DCD  - Insufficient fMRI findings. |
| (Wilson et al., 2020) | To analyse the development of motor skill and EF in school-age children with and without DCD. | N = 52 DCD  N = 134 TD  6-11Y  Gender matched | School | **BOTH:**  1. Brief questionnaire  2. Daily living interference  3. NDI or MAND <85 DCD  4. Exclusion of major medical/neurological conditions and ADHD | ID: NR  ADHD: N  ASD: NR | Y | MAND and  Groton maze learning test (GMLT) completed at T0 and 2-year follow up.  Age band comparison: Younger (6-7Y)  Mid (8-9) and Older (10-11) | GMLT score:  DCD<TD  Mid* Older* | Young: d = 0.325  Mid: d = 0.555  Older: d = 0.508 | 10 | - Children with persistent DCD are at sig risk of EF issues, particularly those 8-11 years.  - Similar rates of EF improvements for DCD to TD, yet different absolute performance level met.  - EF assessment consideration for suspect DCD. |

# **Table 3.** *Dynamical / Ecological Paradigms*

| **Reference** | **Study Objectives** | **Participants**  *Age and/or Gender Matched* | **Referral method**  *School*  *Community*  *Clinical* | **DCD Screening Tool, Cut-Off.** | **Co-occurring disorders in sample**  *Y/N/Not Reported*  *(NR* | **DSM**  **Met**  Y/N | **Task(s) & Outcome measures** | **Key Results** (* significant) | **Effect Sizes** (key group effects) | **Quality Score**  (/10) | **Main Findings** |
| --- | --- | --- | --- | --- | --- | --- | --- | --- | --- | --- | --- |
| (Blais et al., 2017) | Examined the learning of new bimanual coordination in teens with and without DCD. | N = 10 TD  N = 10 DCD  12-16Y | Clinical and community | DCD:  1. Diagnosis of DCD  2. MABC-2 <5th %tile  3. Medical history  4. No ID  TD:  1. MABC-2 >15th %tile  2. No perceptual-motor disorder and medical history  4. No ID | ID: N  ADHD: NR  ASD: NR | N | Executive attention task:  Hold a joystick and produce coordination with thumb and fingers tapping in synchrony to visual stimulations.  Relative phase (RP) of new bimanual coordination.  Absolute error (AE) | Additional taps:  DCD > TD*  Absolute error of relative phase:  DCD > TD* | Additional taps:  d = 0.966  Absolute error of relative phase:  d = 0.69 | 7 | - DCD was less accurate than TD, however, both DCD and TD showed improvements in accuracy of coordination with practice  - No difference in stability between DCD and TD  - The number of additional taps could indicate perceptual-motor learning deficits in DCD. |
| (Blais et al., 2018) | Assess changes in brain area communication during new bimanual coordination tasks in teens with DCD. | N = 10 DCD.  N = 10 TD  Age matched  Aged 12-16 years. | Clinical and community | 1. No ADHD  2. Diagnosis of DCD by paediatrician  3. M-ABC <5^th^ %tile.  4. WISC-V: no ID | ID: N  ADHD: N  ASD: NR | Y | **Three bimanual coordination tasks:** (i) inphase, (ii) antiphase, (iii) new.  Synchronised tapping of left or right thumb based on prompt.  Temporal delay between each condition.  EEG | Relative phase (RP SD):  DCD > TD*.  No. mirror movements pre & post practice**:**  DCD > TD*.  Inter-hemispheric R FC4-FC4 TRCoh* Increase DCD=TD | RP SD *d* = 0.709  MM Pre:  *d = 1.870*  MM Post *d* = 3.015  Interaction FC3-FC4:  *η_p_ ^2^* = 0.15 | 8.5 | - DCD lower stability, fronto-central interhemispheric coherence and < mirror movements than TD.  - Less stable and accurate new bimanual coordination patterns in DCD for ANTIPHASE and NEW coordination tasks.  - In both DCD and TD groups, the accuracy and right fronto-central interhemispheric coherence increased with practice. |
| (Lê et al., 2021) | Test the role of sensory modality of stimulations (visual or auditory) on synchronization, learning and retention of temporal verbal sequences in DCD v TD. | N = 11 DCD  N = 16 TD | Unclear | **BOTH:**  1. MABC-2 <5th %tile DCD and >15th %tile TD  3. Exclusion of ADHD, SLD, Dyslexia, ID | ID: N  ADHD: N  ASD: NR | N | Synchronization task to (a) regular temporal sequence and (b) practice and recalled novel non-regular temporal sequences and MRI scanned  Behavioural: number of errors, vector angle and length.  Cerebral: cortical thickness of three ROIs | Auditory sequence:  Synchronization  Learning*  Retention*  DCD < TD  Cortical thickness  DCD = TD. | Synchronization (errors) d = 0.268  Learning (vector length) d = 1.030  Retention  (errors) d = 1.676 | 7.5 | - Generalised deficit in synchronisation of regular temporal verbal sequence irrespective of sensory modality in DCD  - Synchronisation deficits are not limited to manual tasks for DCD  - Both groups increase accuracy during practice, however sequence isn’t memorised  - DCD deficit when sequence is non-regular and in the auditory modality |
| (Purcell et al., 2017) | Examine perceptual-motor and coordination skills to safely cross a road in DCD. | N = 25 DCD  N = 25 TD  Gender and age matched  6-11Y | School | **BOTH:**  1. Motor difficulties  2. MABC-2 – TD>25th and DCD<16th %tile  4. CPM – non-ID | ID: N  ADHD: NR  ASD: NR | Y | Virtual desktop to determine crossing gaps between traffic at 20,30 or 40m/ph.  Temporal gaps: mean data | Main effect of group on gap acceptance thresholds  DCD<TD* | Group  ηp 2 = 0.16 | 9 | - Strategies utilised would result in a collision for TD at and above 40 m/hr and for DCD at and above 20 m/hr.  - Demonstrate a deficit in visually guided behaviour in DCD in the context of road crossing and this could be related to opportunities for practice. |
| (Roche et al., 2016) | Investigate ability of children with DCD to motorically adapt to perceptible/subliminal changes of auditory stimuli | N = 19 DCD  N = 17 TD  5-11y  Age and gender matched | Clinical | **DCD**  1. MABC <5th %tile  2. DCD diagnosis from paed  **BOTH**  2. PANESS  3. Clinical neurological exam  4. WJ PE Battery – no cog impairment  5. Annett handedness qn | ID: N  ADHD: NR  ASD: NR | Y | Bimanual tapping: To an antiphase beat, tap to perceptible rhythm or gradual subliminal rhythm changes**.**  SD of mean relative phase between two fingers | Perceptual thresholds  (P and S cond*)  DCD = TD.  Group X Age  Young<Older* DCD and TD | P cond:  d = 0.369  S cond: d = 0.527 | 9 | - DCD children were able to perceive changes in rhythm and show a developmental trend comparable to TD  - DCD also able to modulate finger tapping and perceive abrupt and subliminal gradual changes in stimuli  - DCD more variable on motor perf than TD |
| (Wilmut et al., 2017) | Examine perceptual judgements and motor behaviour of children with DCD while looking/walking through apertures | N = 29 DCD  N = 29 TD  7-17Y  Age and gender matched | Clinical and Community | **DCD:**  1. MABC-2: <16th %tile  2. DCDQ and MABC Checklist (BOTH)  3. Telephone interview  4. SDQ | ID: NR  ADHD: NR  ASD: NR | Y | 1. Visual estimation:  absolute, constant, and variable error. Perceptual action judgement: critical ratio (CR) of  shoulder and body width  2. Movement adaptations: walking through a doorway. CR/Speed/Movement | 1. Absolute, constant & variable error DCD=TD  1.CR  DCD<TD*  2. Shoulder CR  DCD>TD*  2. Approach speed  DCD<TD Small SA Ratios  (0.9, 1.1, 1.3) | 1. CR  d = (0.1-.02)  2. S CR d = 0.986  2. Speed d =  (0.2-0.3) | 9.5 | - DCD children are seen to under-estimate their passibility judgements.  - Perception within a static context is different from a dynamic context for children with DCD  - Clear relationship between perception and action in DCD. |

# **Table 4.** *Motor Imagery, Action Observation, Imitation and Gesture Production*

| **Reference** | **Study Objectives** | **Participants**  *Age and/or Gender Matched* | **Referral method**  *School*  *Community*  *Clinical* | **DCD Screening Tool, Cut-Off.** | **Co-occurring disorders in sample**  *Y/N/Not Reported*  *(NR* | **DSM**  **Met**  Y/N | **Task(s) & Outcome measures** | **Key Results** (* significant) | **Effect Sizes** (key group effects) | **Quality Score**  (/10) | **Main Findings** |
| --- | --- | --- | --- | --- | --- | --- | --- | --- | --- | --- | --- |
| (Adams et al., 2017) | Examine the development of motor imagery and action planning in children with DCD over developmen. | N = 30 DCD  N = 30 TD  6-11Y | Paediatric physical therapists and school | DCD  1. MABC-2 <16th %tile  2. DCDQ  4. Onset in early dev and IQ >70  5. ADHD exclusion  TD  1. MABC-2 >20th %tile | ID: N  ADHD: N  ASD: NR | Y | Action planning tasks with differing end point control: Hand rotation task (motor imagery) and sword task (action planning) to examine predictive control and end-state comfort (ESC)  Motor imagery: Mean response times lateral v medially orientated (RTs)  Anticipatory action planning: ESC % of accuracy | Errors: All conditions at all time points  DCD>TD*  ESC:  T0: DCD<TD*  T1: DCD=TD  T2: DCD=TD | Errors:  Range d = 0.554 – 0.895  ESC:  T0: d = 0.574  T1: d = 0.429  T2: d = 0.181 | 9 | - DCD slower and less accurate than TD on motor imagery ability (longer lateral RT) over time.  - Improvements seen between T0 and T1 for DCD, however, still delayed when compared to TD.  - Results support the developmental delay hypothesis for DCD as motor imagery skills were seen to improve over time via longitudinal modelling. |
| (Adams et al., 2018) | Examine development of motor imagery ability of goal-directed pointing task. | N = 30 DCD  N = 30 TD  6-11Y  Gender and age matched. | Paediatric physical therapists and school. | **DCD**  1. MABC-2 <16^th^ %tile  2. DCDQ  4. Onset in early dev and IQ >70  5. ADHD exclusion  **TD**  1. MABC-2 >20^th^ %tile  2. IQ >70 | ID: N  ADHD: N  ASD: NR | Y | Computerised visual radial Fitts’ task (C-VRFT)  Target circles were displayed on a screen which children had to touch.  Correlations between executed and imagined movements. | DCD<TD*  Correlation between executed and imagined movements.  DCD=TD  measurements over following two years. | **T0:**  *d* = 0.618  **T1:** (one year after) *d* = 0.975  **T2:** (two years after)  *d* = 1.192 | 9 | - Evidence supports a delayed developmental onset of motor imagery ability in DCD with a similar rate of development overtime between DCD and TD |
| (Bieber et al., 2021) | Develop a new protocol for assessing action observation (AO) abilities and imitation of meaningful/non gestures in DCD. | N = 12 DCD  N = 11 TD  6-10years  Age-matched | Clinical and community | DCD:  1. MABC-2 <16th %tile  2. DCDQ  3. Early dev period onset  4. WISC-IV and neurological Ax  TD:  1. MABC-2 >25th %tile | ID: N  ADHD: NR  ASD: NR | Y | Test of action observation: Execution of different strategies for simple and complex assembly tasks after observing videoclips showing strategies.  Imitation proficiency: performance of a meaningful (M) or a non-meaningful (NM) gesture.  Total score | DCD<TD  AO*  Imitation proficiency  (M* and NM) | AO d = 1.182  Meaningful d = 0.844  Non-meaningfuld = 1.276 | 8.5 | - Psychometric properties of the new tests were supported  - Confirmation of reliability properties  - Both meaningful and non-meaningful gestures were reduced in DCD  - Reduction with non-meaningful imitation and further research required to determine the underlying mechanisms. |
| (Fuchs and Cacola, 2018) | Compare motor imagery accuracy (MI) and MI vividness between TD and DCD. | N = 51 TD  N = 42 DCD  7-12Y | Unclear | 1.KBIT-2 no ID  2. No comorbidities (ADHD, ASD)  KBIT-2 testing.  3. MABC-2 DCD: Red/amber zone and TD green |  | Y | MIQ-C and FPIQ to assess MI vividness and accuracy.  Average score of questionnaires M(SD) | MI accuracy:  DCD < TD*  MI vividness:  DCD = TD | MI accuracy:  Range d = 0.147 – 0.519  MI vividness:  Range d = 0.177 – 0.285 | 9 | - MI deficit in DCD may be associated with MI accuracy not vividness.  - Significant problems identified with the conception and mastery of MI accuracy. |
| (Gauthier et al., 2018) | Compared development of motor imitation (own-body transformation: OBT) abilities across age in children with ASD and DCD. | N = 14 DCD  N = 36 TD  N = 25 ASD  Age matched, range 6-19 years. | Clinical | DCD:  1. Available historical clinical information.  M(SD)  2. M-ABC:  -1.49(1.60)  WISC-IV.  96.69(17.54) |  | Y | Seven trials of an adapted ‘funamabule’ tightrope walker (TW) paradigm.  No. and description of the tagged movements throughout tightrope trials. | No. tagged movements* (TM)  DCD<TD OBT < with age | Average no. TM d = 1.104 | 7.5 | - Strong developmental age effect: increase of tagged movements and decrease of latency response with age (OBT).  - Lower OBT performance in front-facing orientation DCD group when compared to both TD and ASD. |
| (Nobusako et al., 2018) | Investigate the differences between visuo-motor temporal integration in internal model and automatic-imitation function in MNS between PDCD and TD | N = 29 pDCD  N = 42 TD  Sex, age and handedness matched | School | BOTH:  1. MABC-2 DCD <15th %tile TD >16th %tile  2. Exclusion criteria: diagnoses, conditions, ID | ID: N  ADHD: NR  ASD: NR | Y | Delayed feedback detection and motor interference tasks. Where hand reflection was watched through a double-sided mirror.  Visuo-motor temporal integration ability: delay-detection threshold (DDT) and probability curve of DDT | DDT msec  pDCD>TD*  DD Steepness pDCD<TD*  Motor interference effect between congruent/incongruent conditions:  DCD<TD | DDT: d = 1.194   DD: d =0.478  Int: d = 0.48 | 8 | - Prolonged DDT and decreased steepness is reflective of decreased visuo-motor temporal integration ability.  - Deficits exist in visuo-motor temporal integration and automatic-imitation function for those with pDCD  - DDT was the greatest predictor of poor manual dexterity  - Supports IMD and MNS hypotheses |
| (Reynolds et al., 2017) | Examine imitation of complex novel postures and sequences of gestures in children with and without DCD | N = 29 pDCD  N = 29 TD  Age matched | Clinical and community | BOTH:  1. MABC-2 %tile: <16th DCD >20th TD  2. Exclusion of ADHD & ASD | ID: NR  ADHD: N  ASD: N | N | Two body postural imitation components of the SPIT.  No. correct performances of postural praxis and gestural sequences. | Postural imitation:  pDCD < TD*  Sequencing imitation:  pDCD < TD*  < group differences with < task complexity | Postural imitation:  d = 1.547  Sequencing imitation:  d = 0.912 | 4.5 | - Children with pDCD have difficulty imitating complex novel postures and gesture sequences  - Highlight’s possibility of dysfunction of the MSN and processes such as visual attention, processing, working memory and EF.  - High variability of imitation proficiency provides evidence for heterogeneity and possible subtyping of DCD |
| (Scott et al., 2019) | Assess effects of combined action observation and motor imagery on automatic imitation in children with and without DCD. | N = 12 DCD  N = 12 TD  7-12Y | Community | **BOTH:**  1. MABC-2: TD >20^th^ %tile and DCD <16^th^ %tile.  2. DCDQ  3. IQ>70 assumed by mainstream education  4. ADHD questionnaire and lack of neurological/visual impairments | ID: N  ADHD: N  ASD: NR | Y | Rhythmical action activity of face washing or paint brushing with a rhythmical distractor before, during or without.  Mean response cycle times (ms) and ratio (%) between slow and fast distractor trials. | No sig group differences between DCD/TD  AO and MI instruction conditions < in performance. | No group effects | 8.5 | - Combined AO + MI instructions can significantly enhance automatic imitation effects in children with DCD/TD  - Relative to AO in both TD/DCD and MI in DCD. |
| (Scott et al., 2020) | Assess effects of combined action observation and motor imagery on intentional imitation in children with and without DCD. | N = 13 DCD  N = 12 TD  7-12Y | Community | BOTH:  1. MABC-2: TD >20th %tile and DCD <16th %tile.  2. DCDQ  3. IQ>70 assumed by mainstream education  4. ADHD questionnaire and lack of neurological/visual impairments | ID: N  ADHD: N  ASD: NR | Y | Observed and/or imagined before executing familiar rhythmical pantomime action. Target actions either habitually fast or slow.  Mean response cycle times (ms) and ratio (%) between slow and fast target actions. | Overall imitation ability:  DCD < TD*  DCD>TD  Advantage for AO + MI compared to observe then imagine. | Overall imitation ability:  d = 1.297 | 8.5 | - Combined AO + MI instructions enhance imitation ability when compared to AO followed by MI in TD and DCD. |

# **Table 5.** *Oculomotor Control*

| **Reference** | **Study Objectives** | **Participants**  *Age and/or Gender Matched* | **Referral method**  *School*  *Community*  *Clinical* | **DCD Screening Tool, Cut-Off.** | **Co-occurring disorders in sample**  *Y/N/Not Reported*  *(NR* | **DSM**  **Met**  Y/N | **Task(s) & Outcome measures** | **Key Results** (* significant) | **Effect Sizes** (key group effects) | **Quality Score**  (/10) | **Main Findings** |
| --- | --- | --- | --- | --- | --- | --- | --- | --- | --- | --- | --- |
| (Gaymard et al., 2017) | Investigate oculomotor saccade tasks designed to reveal frontal and cerebellar dysfunction. | N = 11 DCD  N = 11 TD | Clinical | **DCD:**  1. M-ABC  2. WISC-V IQ >70  3. ADI-R | ID: N  ADHD: NR  ASD: N | Y | Oculomotor tasks watching a central point and targets on a screen.  Mean saccade (MS) latency, MS gain and variability of MS gain | *Memory guided saccade:*  MS latency DCD>TD*  *Antsaccade:* Error rate  DCD>TD* | MS Latency:  *d* = 2.011  Error rate:  *d* = 1.317 | 7.5 | - Dyspraxic (DCD) group showed evidence for frontal lobe disfunction  - Due to increased memory-guided saccade latencies, normal visually guided saccade latencies and a decreased ability to cancel reflective saccades  - DCD displayed unambiguous signs of cerebellar dysfunction |
| (Sumner et al., 2018a) | Determine whether oculomotor differences can distinguish between DCD and TD. | N = 23 DCD  N = 25 Age matched TD  N = 29 Motor matched TD | School | **BOTH:**  1. WISC-V FSIQ >80 and MM WPPSI-V  2. MABC-2 <16th DCD and >25th %tile TD  3. SDQ  4. Background screening | ID: N  ADHD: NR  ASD: N | Y | Children noted to watch a red circle against a black background while using eye tracking technology  Visual fixation, smooth pursuit and pro&-anti-saccade performance | *Average fixation duration*  *DCD<TD*.*  *Anti-saccade % direction errors*  *DCD>TD** | Duration: d = 0.574  Errors:  d = 0.951 | 8.5 | - DCD display deficits with saccade inhibition and maintaining attention on a visual target.  - Fundamental oculomotor processes are however intact in DCD.  - DCD display difficulties with top-down control even when compared to younger children. |

# **Table 6.** *Reaching and Manual Control*

| **Reference** | **Study Objectives** | **Participants**  *Age and/or Gender Matched* | **Referral method**  *School*  *Community*  *Clinical* | **DCD Screening Tool, Cut-Off.** | **Co-occurring disorders in sample**  *Y/N/Not Reported*  *(NR* | **DSM**  **Met**  Y/N | **Task(s) & Outcome measures** | **Key Results** (* significant) | **Effect Sizes** (key group effects) | **Quality Score**  (/10) | **Main Findings** |
| --- | --- | --- | --- | --- | --- | --- | --- | --- | --- | --- | --- |
| (da Rocha Diz et al., 2018) | Compare effect of practice on isometric finger force/torque task between at risk of DCD and TD children | N = 12 risk of DCD  N= 12 TD  Age and gender matched  9-10Y | School | **DCDr:**  1. MABC <5th %tile  2. Screened for ADHD, neurological issues  **TD:**  1. MABC >35th %tile  **BOTH**  Right-handed with no upper limb trauma history | ID: NR  ADHD: N  ASD: NR | N | Isometric finger task:  Produce finger force/torque control in a continuous and constant 25% max voluntary torque with/without visual feedback for 15s  Accuracy of force matching performance | *Accuracy of finger torque/force:*  *DCDr < TD** | Accuracy with vision:  d = 0.847  Accuracy without vision:  d = 0.847 | 7.5 | - Both groups of children were able to improve their performance after practice sessions  - DCDr were more reliant on visual feedback to control and produce force  - Precise control difficulties for DCDr could be due to neuromuscular timing (lack of coordination) challenges |
| (Gama et al., 2016) | Investigate use of valid, neutral, and invalid pre-cue information for a goal-directed task. | N = 11 DCD  N = 11 TD  Age and gender matched | School | **DCD:**  1. MABC manual dexterity <5th %tile  **TD:**  1. MABC >30th %tile | ID: NR  ADHD: NR  ASD: NR | N | Pre-cue paradigm:  Marks displayed on a table and a stylus was used to point during valid, invalid and PC conditions.  Reaction (RT), movement (MT), deceleration time in response to instruction. | *Invalid condition (IC)*  *DCD<TD**  *Valid condition*  *DCD=TD*  *Group and condition main effects** | IC RT  d = 0.996  IC MT d = 0.937  Group ηp 2 = 0.33  Condition ηp 2 = 0.48 | 3.5 | - DCD slower than TD at initiating and executing motor responses  - Increasing deficiency as a function of condition complexity from valid – invalid pre-cue condition in DCD  - TD process spatial and temporal information prior to task initiation, however, DCD start movement earlier despite less information processed. |
| (Golenia et al., 2018) | Examined variability in coordination patterns of goal-directed reaching arm movements. | N = 11 DCD  N = 11 TD  Age matched  Age 6-11years | School and clinical | **DCD:**  1. MABC-2 <16th %tile  2. DCD diagnosis through outpatient service  **TD:**  1. MABC-2 >16th %tile | ID: NR  ADHD: NR  ASD: NR | Y | Apparatus and procedure reaching tasks to targets on a television screen.  Comparing variability that does (Vucm) and doesn’t (Vort) ­affect index fingertip position (UCM measures) and movement time (MT). | *Reaching errors:*  *DCD = TD*  *Movement time:*  *DCD > TD**  *Coordination variability (Vucm):*  *DCD > TD**  *Coordination variability (Vort):*  *DCD = TD* | Reaching errors:  d = 0.516  Movement time:  d = 1.06  Coordination variability (Vucm):  d = 0.86  Coordination variability (Vort):  d = 0.316 | 9.5 | - Similar reaching errors between DCD and TD, however MT longer and more variable in DCD  - Higher motor variability seen in DCD, however, this did not affect their performance |
| (Gonzalez et al., 2016) | Explore the integrity of saccadic eye movement control in DCD and TD | N = 10 DCD  N = 12 TD  8-12years | Clinical | **DCD:**  1. External DCD diagnosis by medical practitioners  **TD:**  1. No history of motor, eye or cog deficits.  **BOTH:**  1. No ADHD and all right-handed | ID: N  ADHD: N  ASD: NR | Y | Children sat in a chair and responded to stimuli targets with their eyes, hand or both.  Eye movements: inhibitory control: mean ratio of no. saccades and no. total trials/condition  Hand responses:  Absolute error rate from target’s position | *Eye and hand movements*  *DCD=TD*  *Cued condition: Saccades away from fixation - inhibition error DCD>TD** | Inhibition errors: d = 2.541 | 7.5 | - Similar saccade kinematics for DCD compared to TD during saccade initiation.  - Deficits identified in more complex control processes involving prediction and inhibition for DCD.  - The saccadic control could play a role in acquisition of complex skills. |
| (Hsu et al., 2018) | Examine construct validity of the Motor Planning (Maze) Assessment, Functional Gait Assessment (pmFGA) and Dynamic Gait Index (DGI). | N= 20 DCD N = 20 TD  Age matched  Age 5-12years | School & clinical | **DCD:**  1. DCDQ scores indicate DCD/suspect DCD  2. Diagnosis of DCD  3. MABC-2 <15th %tile.  **TD:**  1. DCDQ no problems  **BOTH:**  2. IQ <60  3. No motor related diagnoses/conditions | ID: N  ADHD: NR  ASD: NR | N | Children completed the MAZE, DGI and FGA assessments. | *Maze:*  *Time to complete and summary score*  *DCD>TD**  *pmFGA:*  *Summary score*  *DCD<TD** | Item 1 time to complete:  d = 1.019  Maze summary score: d = 1.776  PmFGA summary score:  d = 1.637 | 6 | - Maze test and pmFGA are sensitive to differentiate between DCD and TD  - DGI and FGA were sensitive enough to differentiate between DCD and TD |
| (Warlop, Vansteenkiste, Lenoir, & Deconinck, 2020a) | Explored differences and similarities in gaze behaviour in young adults with DCD and TD. | N = 10 TD  N = 10 DCD  Age matched  20-23years |  | **DCD:**  1. Formal diagnosis by paediatrician during childhood  2. Re-assessed using MABC-2 **and were <16th %tile**  **TD:**  1.. MABC-2 >25th %tile  2. Normal or corrected-to-normal vision | ID: NR  ADHD: NR  ASD: NR | Y | Reading and cup stacking activities while a remote eye tracking device which registered gaze.  Eye movement measures during reading, cup stacking and hand and hand-eye movement time during cup stacking. | *DCD>TD*  *Reading time**  *Speed of cup stacking (UM*/BM)*  *Fixations:*  *No. unimanual (UM)* and bimanual (BM) stacking. No. reading** | Time  d = 0.895  Cup Speed (UM)  d = 1.856 (BM)  d = 0.794  Fixations:  Cup 3(UM) d = 1.851  Reading d = 0.948 | 8 | - Tendency towards slower reading times shown in DCD, however, oculomotor problems did not profoundly affect reading in DCD adults  - Cup stacking, DCD demonstrated a different gaze strategy to TD by using gaze locking to the targets and a higher number of fixations during UM and similar gaze behaviour during BM tasks. |

# **Table 7.** *Internal modelling (incl. Prospective Reaching/Grasping)*

| **Reference** | **Study Objectives** | **Participants**  *Age and/or Gender Matched* | **Referral method**  *School*  *Community*  *Clinical* | **DCD Screening Tool, Cut-Off.** | **Co-occurring disorders in sample**  *Y/N/Not Reported*  *(NR* | **DSM**  **Met**  Y/N | **Task(s) & Outcome measures** | **Key Results** (* significant) | **Effect Sizes** (key group effects) | **Quality Score**  (/10) | **Main Findings** |
| --- | --- | --- | --- | --- | --- | --- | --- | --- | --- | --- | --- |
| (Adams et al., 2017a) | Testing predictive control of movement in children with DCD. | N = 33 DCD  N = 33 TD  6-11Y | Physical therapists | DCD  1. MABC-2 <16th %tile  2. DCDQ  4. Onset in early dev and IQ >70  5. ADHD exclusion  TD  1. MABC-2 >20th %tile | ID: N  ADHD: N  ASD: NR | Y | Action planning tasks: Sword task to examine predictive control and end-state comfort (ESC)  Rapid online control (ROC):  Double-step reaching paradigm. Mean RTs and no of errors.  Anticipatory action planning: ESC % of accuracy | DCD<TD*  Critical trials of AAP  Motor imagery RTs  DCD=TD  ROC Task | **RTs main effect of group:**  ηp2 = 0.39 | 8. 5 | - Evidence supports deficits of internal modelling of movements  - Task related factors constrain the expression, particularly movement complexity |
| (Bhoyroo et al., 2018) | Clarify nature of motor planning in DCD. | N = 10 DCD  N = 17 TD  Males  8-12Y | School and Clinical | **BOTH**  1. MAND NDI <85 DCD and >90 TD.  2.DCDQ  3. Exclusions for neurological, movement or ADHD | ID: N  ADHD: N  ASD: NR | Y | Action planning tasks with differing end point control: Simple (bar grasping, sword and bar transport) and complex (octagon tasks) and measure of ESC.  Motor planning measured through ESC for simple v complex tasks. | *ESC:*  *Simple measures:*  *DCD=TD*  *ESC:*  *Complex measures:*  *DCD < TD** | ESC:  Simple measures:  d = 0.273  ESC:  Complex – 2 colours:  d = 1.263 | 7.5 | - DCD show ability to plan simple movements as efficiently as TD peers  - Difficulties are evident for DCD with multi-movement or complex sequences |
| (Bhoyroo et al., 2019) | Investigate prevalence of end-state-comfort (ESC) and minimal rotation strategy with or without instructions. | N = 14 DCD  N = 18 TD  Males  7-12Y | School and clinical | **BOTH**  1. MAND NDI <85 DCD and >90 TD.  2.DCDQ  3. Exclusions for neurological, movement or ADHD  4. IQ > 70 | ID: N  ADHD: N  ASD: NR | Y | Prospective reaching/ grasping judgement: Completion of octagon (MP) task with (MIP) or without instructions (MP).  % of ESC | *ESC and minimal rotation:*  *Motor planning only:*  *DCD < TD**  *Motor imagery and planning:*  *DCD = TD* | Motor planning only:  d = 1.299 | 8 | - DCD were less likely to end in ESC than TD peers and instead utilised a minimal rotation strategy due to less optimal planning  - When instructions were provided the ESC was more often used and improved performance in DCD. |
| (Cignetti et al., 2018) | Examined the impact of feedforward control in DCD. | N = 11 DCD  N = 18 TD  8-12Y | Clinical | **All:**  1. WISC-IV >70  2. Aloutte and ODEYS-2  3. MABC <5th %tile DCD and >20th %tile TD  4. Right-handed | ID: N  ADHD: NR  ASD: NR | Y | Bimanual unloading paradigm: load either unexpectedly or voluntary unloaded  Presence of Anticipatory Postural Adjustments (APAd) and mean maximal amplitude (MA). | Anticipation of unloading. Presence of APAs  DCD=TD  Unloading accuracy DCD<TD | Mean MA Group main effect: Condition ηp 2 = 0.82  Group:  ηp 2 = 0.08 | 8.5 | - All children relied on anticipatory postural adjustments in voluntary unloading  - DCD may have more difficulties with future state predictions (online control) rather than the inverse models of feedforward control |
| (Gomez-Moya et al., 2020) | Evaluate implicit and explicit motor learning capabilities in children with DCD. | N = 40 DCD  N = 40 TD  Age and sex matched  4-12 years | Unclear | **DCD:**  1. Diagnosis by clinical neuropsych 2. MABC <5th %tile  **TD:**  1. MABC >20th %tile | ID: NR  ADHD: NR  ASD: NR | Y | Repeatedly throw a ball towards a target and adjust for prism differences  Variable error distance and magnitude and magnitude adaptation and aftereffect. | Variable error:  DCD > TD*  Procedural visuomotor adaptation:  DCD < TD*  Strategic visuomotor adaptation:  DCD < TD* | Variable error:  *d* = 0.272  Procedural visuomotor adaptation:  *d* = 0.494  Strategic visuomotor adaptation:  *d* = 0.497 | 8 | - Identified procedural and strategic visuomotor deficits in DCD  - Smaller adaptation, aftereffect, and impairment in implementation of cognitive strategies to reduce error  - Speculate possible neural basis of sensorimotor processing deficits. |
| (Krajenbrink et al., 2021) | Determine whether children with or without DCD were more likely to plan for ESC when complex object manipulations are demanded. | N = 9 DCD  N = 9 TD  Gender and age matched.  5-11y | Unclear | **BOTH:**  1. MABC <16th %tile DCD and TD >16TH %ttile  2. DCDQ (DCD)  3. Treated for MQ difficulties (DCD)  3. No cog/neurological impairment  4. No ADHD (parent report) | ID: N  ADHD: N  ASD: NR | Y | Second-order motor planning measures:  Sword and hammer task.  Proportion of ESC on critical task trials | *Plan for ESC*  *Hammer>SwordDCD = TD*  *ESC group*  *DCD =TD* | Critical trials  Sword  d = 0.913  Hammer  d = 0.585 | 8.5 | - ESC planned for by TD and DCD on hammer rather than sword task.  - Additional force and speed demands used in the hammer task elicit more ESC planning  - General trend of TD ending more often in ESC than DCD, however, limited by no group differences. |
| (Opitz et al., 2020) | Aimed to delineate between the different aspects of internal forward modelling across several domains. | N = 24 DCD  N = 23 TD  Age 7-10y | Community | **BOTH:**  1. MABC-2: <5th %tile severe, 6-15 mod and >15th TD.  2. WISC-V no ID  3. CELF-4  4. Conners-3  5. TOWRE-2 | ID: N  ADHD: N  ASD: NR | N | Sequence detection game: where serial prediction tasks (SPTs) were used to assess visuoperceptual, spatial and temporal processing.  ERP waveform: % area latency (MS) at Pz electrode | % MS  DCD<TD  Visual*  Spatial*  Temporal  Motor*  No evidence for impairment in implicit performance on the visuomotor task in DCD. | Visual  d = 22.156  Spatial  d = 10.703  Temporal  d = -1.531  Motor  d = 22.392 | 9.5 | - Deficit suggests a domain-general delay in processing stimulus change rather than just motor impairment in DCD.  - Suggest an impairment in the updating of an internal forward model which results in a blurred representation and a reduced ability to detect regularities in the environment. |

# **Table 8.** *Catching*

| **Reference** | **Study Objectives** | **Participants**  *Age and/or Gender Matched* | **Referral method**  *School*  *Community*  *Clinical* | **DCD Screening Tool, Cut-Off.** | **Co-occurring disorders in sample**  *Y/N/Not Reported*  *(NR* | **DSM**  **Met**  Y/N | **Task(s) & Outcome measures** | **Key Results** (* significant) | **Effect Sizes** (key group effects) | **Quality Score**  (/10) | **Main Findings** |
| --- | --- | --- | --- | --- | --- | --- | --- | --- | --- | --- | --- |
| (Licari et al., 2018) | Investigate visual tracking behaviour during catching in children with DCD | N = 11 DCD  N = 13 TD | Community | **BOTH:**  1. MABC-2 – TD >16^th^ %tile & DCD <5^th^%tile  2. Snellen chart  3. Vanderbilt ADHD parent rating scale  4. Height and weight | ID: NR  ADHD: N  ASD: NR | N | Best 5 central catching trials used to evaluate visual tracking behaviour and motor responses.  Motor fixations and duration time. Object recognition and tracking phase (M no. fixations, no. blinks and blink duration) | Object recognition no. Fixations. Time to smooth pursuit  DCD> TD*  Movement initiation time  DCD<TD | No. Fixations: *d* = 0.870  Time to SP: *d =* 1.372  Time: *d* =-0.579 | 9 | - DCD fixated on more external locations rather than the ball prior to release  - Evidence ‘unstable’ gaze with a larger no. of fixations for a shorter duration for DCD  - DCD more prone to distractions and struggle with maintaining overt attention on the ball prior to release  - Inefficient gaze behaviour prior to and immediately after ball release which may impact more complex catching tasks |
| (Wattad et al., 2020) | Compare VR game performance in DCD and TD | N = 20 TD  N = 10 DCD  Age matched | Clinical | **BOTH:**  1. DCDQ: <46 DCD  2. MABC-2: %tile <5th DCD, 5th - <16th rDCD, >16th TD  3. Excluded due to ASD, ADHD, Medical conditions, visual impairment | ID: NR  ADHD: N  ASD: N | Y | Completion of non-immersive VR game on pressure pad  Game score, hand path length, CoP movement | VR Game score  DCD<TD*  CoP movement pattern and hand path length  DCD=TD. | Score:  d = 0.825 | 5.5 | - VR game provided prelim distinction between TD and DCD children.  - Evidence that hand path length may reflect visual-motor coordination impairment of children.  - Pressure sensing pad may not be appropriate to discrim between TD and DCD. |

# **Table 9.** *Gait (incl. Visual Control of Gait and Clinical Assessment)*

| **Reference** | **Study Objectives** | **Participants**  *Age and/or Gender Matched* | **Referral method**  *School*  *Community*  *Clinical* | **DCD Screening Tool, Cut-Off.** | **Co-occurring disorders in sample**  *Y/N/Not Reported*  *(NR* | **DSM**  **Met**  Y/N | **Task(s) & Outcome measures** | **Key Results** (* significant) | **Effect Sizes** (key group effects) | **Quality Score**  (/10) | **Main Findings** |
| --- | --- | --- | --- | --- | --- | --- | --- | --- | --- | --- | --- |
| (Gentle et al., 2016) | Examine step characteristics of those with and without DCD while walking on irregular terrain. | N = 35 DCD  N = 35 TD  Gender and age matched groups.  1 8-12  2 13-17y  3 18-32y | Clinical | **DCD:**  1. MABC-2 <15th %tile & BOT-2 <18th %tile  2. ADC interview for ADL and childhood onset  3. CAARS-S  4. SDQ for 4-16y  **TD:**  1. MABC-2 >16th %tile | ID: NR  ADHD: NR  ASD: N | Y | VICON 3D motion analysis with infrared cameras tracked body motion along a level and irregular walkway.  Walking velocity, proportional step length, head and trunk angles/ | Irregular terrain  Step length, velocity, head angle  DCD < TD  Step width  DCD>TD | Main group effects  Walking velocity:  ηp 2 = 0.17*  Prop step length:  ηp 2 = 0.18*  Step width ratio:  ηp 2 = 0.14* | 9 | - Both groups made gait pattern adaptations whilst walking on irregular terrain  - Irregular terrain created a larger impact for DCD  - DCD showed greater reduction in step length, velocity and head angle and greater increase in step width than TD  - These changes are thought to preserve stability when there are balance changes. |
| (Mannini et al., 2017) | Evaluates an IMU-based method to automatically and objectively assess paediatric gait in Early Onset Ataxia and DCD. | N= 7 DCD  N = 20 TD  N = 10 EOA  Age matched | Clinical | **DCD:**  1. DSM-IV-TR impaired coordination  **TD:**  1. Ability to follow mainstream education  2. Absence of neurological disorders/physical conditions | ID: N  ADHD: NR  ASD: NR | N | Completed SARA gait evaluation assessment.  SARA gait and gait sub scores.  No. Strides  (0 = no diffs, 8 = unable to walk) | SARA  Total Score Gait Sub score  DCD>TD* | TS: d = 1.224  Gait Score: d = 0.904 | 7.5 | - Quantitative gait features can provide a supportive tool for unanimous and reproducible diagnostic assessment |
| (Nieto et al., 2018) | Investigate information processing through visual information restriction during walking tasks in at risk of DCD v TD | N = 16 TD  N = 16 DCDR  Age matched | School | **At risk of DCD:**  1. Multi-d ax  2. MABC-2  3. Sound physical and psychological health | ID: NR  ADHD: NR  ASD: NR | N | Walk 10m walkway at self-selected speed under 4 visual conditions.  Gait kinematics. | Walk speed*, step length and stride  DCD<TD  Group x vision interactions*: single support, load response and pre-swing | Speed ηp 2 = .0.334  G X V  d = 0.70-1.3 | 8 | - Visual information withdrawal may impact the gait cycle differently for DCD and TD children.  - TD children able to use proprioceptive feedback under non-visual conditions in comparison to DCDr.  - Walking performance of TD children varied regardless of visual conditions  - Speed and step distance variations seen between TD and DCDr |
| (Parr et al., 2020) | Examined stepping accuracy, gaze behaviour and state-anxiety in children with and without DCD | N = 21 DCD  N = 18 TD | Community | **DCD:**  1. DSM-5 Criteria  2. DCD1  3. MABC-2 <15th %tile  **TD:**  1. MABC-2 <15th %tile  **BOTH:**  Right footed  ADHD rating scale <98th %tile | ID: NR  ADHD: N  ASD: NR | Y | Walking along pathway at self-selected pace either with or without obstacles  Accuracy of foot placement (AP) and precision (ML)  Gaze fixations and location (AOI’s) | Main effect of group on absolute AP error and constant AP error*  DCD>TD  Gaze behaviour  DCD=TD | Group on absolute AP error:  ηp 2 = .0.363  Group on constant AP error:  ηp 2 = .0.159 | 8.5 | - DCD less accurate than TD when needing to precisely place their foot within a floor-based target  - Decreases in foot placement accuracy and precision may be partially responsible for increased incidence of trips and falls in DCD  - Difficulties with stepping actions in DCD occur independent to anxiety.  - Falls risk may be better explained by neuromuscular control deficits. |
| (Parr, Foster, Wood, Thomas, et al., 2020a) | Investigate the visuomotor control strategies underpinning stair negotiation in children with and without DCD. | N = 16 TD  N = 18 DCD | Community and clinical | **DCD:**  1. DCDQ  2. No learning, sensorimotor difficulties  3. MABC <15^th^ %tile  4. ADHD rating scale  **TD**  1. DCDQ above indication zone  2. MABC >15^th^ %tile | ID: N  ADHD: N  ASD: NR | Y | Children ascended and descended a staircase with a handrail alongside.  Kinematics, gaze behaviour and state anxiety. | DCD>TD  Ascend(A)/descend (D)* (s)  Handrail use*  Fixation duration (ms)  Var in foot clearance* (cm) | **Handrail use:** (A) *d* = 1.087 (D) *d = 0.927*  **Fixation duration:**  (A) *d* = 0.280 (B) *d* = 0.232  **Foot-clearances**:(A) *d* = 0.872 (B) *d* = 1.241 | 9.5 | - Safe stair negotiation is a significant challenge for children with DCD  - Evidenced by marked differences in visuomotor control strategies and state anxiety levels  - Children with DCD experience sig more stair related falls than TD.  - Unclear whether visuomotor control strategies are mitigating or contributing to stair-related falls. |
| (Speedtsberg et al., 2018) | Local dynamic stability of trunk acceleration during treadmill walking: objective evaluation of gait stability and sensitivity in DCD. | N = 8 DCD  N = 10 TD  Age and gender matched | Clinical | **DCD:**  1. MABC2 <15th %tile  2. DSM-IV criteria  3. No comorbid  **TD:**  1. MABC2  >16th %tile  2. No ND Disorders | ID: N  ADHD: N  ASD: N | Y | 4 min treadmill walking assessing trunk accelerations via a sternum mounted accelerometer  ST local dynamic stability. | Lyapunov Exponent  (λs) on AP direction  DCD>TD*  (λs) on Vertical and ML:  DCD<TD | λs AP:  d = 1.650  λs Vertical: d = 0.970  λs ML: d = 0.940 | 8.5 | - Local dynamic stability could be responsive when quantifying gait stability impairments in DCD  - Fundamental impairment in local stability of normal gait in DCD  - Integration of proprioceptive feedback info and spinal control could play a sig role in explaining gait instability in DCD |
| (Warlop et al., 2020) | Differences and similarities in gaze behaviour during walking between TD and DCD. | N = 10 DCD  N = 10 TD  Adults  Gender and age matched | Clinical/ community | **DCD:**  1. Formal DCD diagnosis  2. MABC-2<16th %tile  3. Normal or corrected to normal vision  **TD:**  1. MABC >16th %tile | ID: NR  ADHD: NR  ASD: NR |  | Walking tasks where feet had to be placed on irregular targets while wearing eye tracking glasses.  M and SD of locomotor behaviour and eye movements. | Walking speed  DCD>TD*  Fixations 1 target ahead DCD>TD*  Fixations more targets ahead  DCD<TD* | Speed:  d = 1.211  Fixations  1 Target ahead:  d = 1.937  More targets: d = 1.918 | 9 | - Fundamental control deficits persist into adulthood for DCD  - DCD exhibit varied gaze strategy for negating a stone path: directed to each stone while heading towards rather than further forward gaze on path around stones in TD  - Young adult DCD gaze strategies reflect unstable oculomotor control and an increased dependency on on-line foveal visual feedback of the walking path |
| (Wilmut & Barnett, 2017a) | Examine how individuals with and without DCD adjust to the possibility of an obstacle appearing which requires circumvention. | N = 44 DCD  N = 44 TD  7-34Y  Age and gender matched | Clinical and community | **BOTH:**  1. MABC-2 <17Y: <16th %tile DCD  2. BOT-2 >17Y <18th %tile DCD  3. DCDQ and MABC Checklist | ID: NR  ADHD: NR  ASD: NR | Y | Walking along clear path task:  Anticipatory control: Spatial-temporal parameters of foot placement, trunk movement and path deviation. | Medio-lateral acceleration < when obstacle may appear.  DCD=TD.  Path adjustments  DCD sooner and more deviated than TD. | Interaction for step width between condition and age* ηp 2 = .11 | 10 | - Lack of anticipatory movement in DCD may have occurred due to a movement selection choice.  - Inflexibility of the motor system to adjust step width for action and may result in less controlled object circumvention. |
| (Wilmut & Barnett, 2017b) | Investigate exact nature of changes in foot placement during obstacle avoidance in DCD | N = 44 DCD  N = 44 TD  7-34Y  Age and gender matched | Clinical and Community | **BOTH:**  1. MABC-2 <17Y: <16th %tile DCD  2. BOT-2 >17Y <18th %tile DCD  3. DCDQ and MABC Checklist | ID: NR  ADHD: NR  ASD: NR | Y | (See 2017)  No. and magnitude of step length and width adjustments | No. early step adjustments:  Children > adults  DCD=TD  Young children  DCD<TD | Interaction for step number and age* ηp 2 = .24 | 10 | - Choice of foot placement in DCD is in line with TD peers, however, may not be optimal for their level of motor ability.  - Significant age changes with timing of adjustments to foot placement and choice of foot placement strategies. |
| (Wilmut, Gentle, et al., 2017c) | Evaluate whether asymmetrical gait can explain inefficient gait cycle | N = 62 TD  N = 62 DCD  Gender matched | Clinical | **DCD**  1. MABC-2 for <18y  <9th %tile  2. MABC-2 & BOT-MP for >18y  <18th %tile  **DCD & TD**  1. SDQ or CAARS  2. MABC Checklist and phone interview | ID: NR  ADHD: NR  ASD: N | Y | Continuous 1min walking along a walkway monitored by an optical tracking system.  Step length and time for R and L leg and symmetry ratios | Step length and time ratio  DCD>TD* | Length:  d = 0.497  Time: d = 0.784  Effect of group*: ηp 2 = .09 | 8.5 | - Significantly higher symmetry ratio in DCD compared to TD (step length and time)  - Some, not all DCD showed asymmetrical gait  - Greater asymmetry could explain an increase in variability of spatial-temporal gait measurements |
| (Yam and Fong, 2018) | Gait muscle activity and lean muscle mass in DCD. | N = 51 DCD  N = 52 TD | School | **DCD:**  1. Daily life fine/gross motor impact  2. MABC-2 with score <15th %tile  3. DCDQ  **TD:**  1. MABC-2 >15th %tile.  **BOTH:**  1. History of disorders, gait issues = exclusion | ID: NR  ADHD: NR  ASD: NR | Y | Treadmill walking peak muscle activation patterns in the right leg for 8 phases.  Leg lean mass measures evaluated using x-ray scans | Gastrocnemius peak EMGms: heel strike and late swing.  Lean mass: Leg and Appendicular (ALMI)  DCD<TD* | Heel strike:  d = 0.421  Late swing: d =0.576  Lean mass: L & R  (d = 0.36-0.38)  ALMI: d = 0.517 | 8.5 | - DCD displayed lower lean mass measure than TD  - DCD displayed lower leg peak muscle activation during heel strike, early and late swing phases of gait walking  - Need to incorporate lower limb phasic muscle strengthening into gait rehab for DCD |

# **Table 10.** *Postural Control*

| **Reference** | **Study Objectives** | **Participants**  *Age and/or Gender Matched* | **Referral method**  *School*  *Community*  *Clinical* | **DCD Screening Tool, Cut-Off.** | **Co-occurring disorders in sample**  *Y/N/Not Reported*  *(NR* | **DSM**  **Met**  Y/N | **Task(s) & Outcome measures** | **Key Results** (* significant) | **Effect Sizes** (key group effects) | **Quality Score**  (/10) | **Main Findings** |
| --- | --- | --- | --- | --- | --- | --- | --- | --- | --- | --- | --- |
| (Cheng et al., 2018) | Compare reactive balance performance, lower limb muscle reflex contraction latency and attention level in response to unpredictable balance perturbations. | N = 100 TD  N = 120 DCD  6-9Y | Clinical and School | 1. Prior DCD diagnosis  2. MABC <15th %tile  3. DCDQ  4. Normal range intelligence  5. CBCL <70 T score | ID: NR  ADHD: N  ASD: NR | Y | Postural control task:  Motor control test on a computerized dynamic posturagraphy machine.  MTC Latency score: indicating reactive postural response.  Attention level | MTC Latency score (backward)  DCD>TD*  (forward)  DCD<TD*  Attention levels during and after pertubations  DCD<TD | Backward: d = 0.297  Forward: d = 0.275 | 9.5 | - DCD less attentive to postural threats and balance responses were direction-specific.  - Indicates possible lower attentional capacity for DCD.  - Additional emphasis on sudden posterior-to-anterior balance perturbations as well as problems with inattention for children with DCD. |
| (Ganapathy Sankar and Monisha, 2019) | Examine and compare movement difficulties and balance in DCD. | N= 20 DCD  N= 80 TD  8-10years  Age and gender matched | School | **DCD:**  1. BCTC ‘motor difficulties’  2. WISC ‘normal IQ’  3. No physical/  neurological disorder | ID: N  ADHD: NR  ASD: NR | Unclear | Body coordination test for children (BCTC).  Motor Quotient (MQ) and peak-to-peak amplitude of centre of pressure (cop) | Postural sway  DCD>TD | COP displacement (eyes closed) d = 2.085 | 4 | - Greater postural sway in DCD when compared to TD  - Closing of eyes for DCD when balance testing may impact increased postural sway |
| (Jelsma et al., 2020) | Analyse movement control strategies using amplitude and differences in DCD compared to TD. | N= 15 DCD  N= 28 TD  Age 6-11y | School (m/s and special) | DCD:  1. MABC-2 <16th %tile  TD:  1. MABC-2 >50th %tile.  BOTH:  1. WISC or SONR IQ>70 | ID: N  ADHD: NR  ASD: NR | N | Wii Fit ski slalom test (10 repetitions).  19 gates to be passed through each run at varied lateral and forward distances and video observed. Higher Wii score = worse performance. | Pre-test  Passed gates and Mark  DCD<TD  Arm, Hip Feet, Head  Movement score  DCD<TD* | Passed gates:  d = 1.700  Mark:  d = 0.803  Movement Score:  d = (0.55-1.13) | 9 | - DCD children who used small movements were less successful at passing the gates and their marks indicated poor control  - Performance and mark quality may be explained by the less smooth movements of the children with DCD.  - DCD children showed improvements in scores after variable training, possibly due to improved anticipation and reaction rather than adaptation of movements and efficiency.  - DCD inconsistent in use of strategies, but also some DCD can adapt and explore other movement strategies. |
| (Miller et al., 2019) | Use kinematic data to assess the magnitude of differences between ASD and DCD. | N = 10 ASD  N= 10 DCD  N = 8 TD  Age and full-scale IQ matched | Clinical | DCD & ASD:  1. DSM-IV/V prior diagnosis  2. IQ scores >69  2. No neurological/physical conditions | ID: N  ADHD: NR  ASD: Y | Y | Shift centre of pressure (CoP) to match dynamic positions through a visual tracking task on a force plate.  CoP, Velocity, and acceleration.  High velocity: fast movement  High acceleration: less-fluid movement | Mediolateral  CoP Velocity  DCD<TD*  Anterior-Posterior  CoP Acceleration  DCD>TD* | CoP V: d = 0.889  CoP Acc: d = 1.675 | 6 | - Speed profile of movement during a dynamic postural control task differs between ASD & DCD  - DCD show consistently higher accelerations which reflect a lack of movement fluidity  - DCD movement patterns support poor internal modelling hypothesis: slower mechanisms are insufficient and anticipatory control of CoP is necessary to overcome response latency. |
| (Nunzi et al., 2018) | Assess postural function in DCD and Dyslexia | N= 14 DCD  N = 18 TD | Unclear | **DCD:**  1. Diagnosed according to DSM-IV criteria  2. MABC <15th %tile  **BOTH:**  No ADHD, other conditions | ID: NR  ADHD: N  ASD: NR | Y | Sensory organisation Test (SOT) and Dynamic Posturography tests were utilised under 6 different conditions to examine balance control performance. | Balance control in upright standing condition  DCD<TD*  Sway platform tasks  DCD<TD* | Standing condition: d = 0.728  Sway task scores: d = 0.728 | 7 | - DCD adopted different balance strategies than TD and lower on upright and sway platform.  - DCD are more dependent on visual information and have difficulties integrating information to maintain balance. |
| (Speedtsberg et al., 2017) | Investigate underlying mechanisms of impaired postural control in children with DCD. | N = 9 DCD  N = 10 TD  Age and gender matched | Clinical | **DCD:**  1. MABC2 <15th %tile  2. DSM-IV criteria  3. No comorbid  **TD:**  1. MABC2  >16th %tile  2. No ND Disorders | ID: N  ADHD: N  ASD: N | Y | Static bipedal standing balance tested on a force plate under several visual and support surface conditions.  ML: Mediolateral AP: Anteroposterior  Rambling: CNS exploratory  Trembling: peripheral feedback control  Sway length, CoP displacement | Sway length*  Trembling - ML*, AP  Rambling -ML, AP*  DCD>TD | Firm surface, eyes open.  Sway length: *d* = 0.969  Trembling: ML *d* = 3.446  Eyes closed.  Rambling:  AP *d* = 0.917 | 8.5 | - DCD increased trembling and rambling across a variety of sensory conditions.  - DCD impaired postural control could be explained by impaired spinal feedback and peripheral control.  - DCD more susceptible to disturbance in proprioceptive input. |
| (Yam and Fong, 2019) | Lower Quarter Y-Balance Test performance and leg muscle kinetics in DCD. | N = 48 DCD  N = 51 TD | School | DCD:  1. Teacher referring child with motor difficulties.  2. MABC-2 <15th %tile  3. Questionnaire + DCDQ  TD:  1. MABC-2 >15th %tile.  BOTH:  Exclusion for neurological and physical conditions | ID: NR  ADHD: NR  ASD: NR | Y | YBT-LQ performance and leg muscle kinetics while completing the test. | Overall YBT-LQ score*.  Peak EMGms gastrocnemius medialis activation*.  Muscle time-to-peak torque.  DCD<TD | Score: d = 1.058  Peak EMGms: d = 0.447 | 10 | - Less competent YBT-LQ performance and atypical neuromuscular control seen in DCD.  - Different peak muscle activations and muscle time-to-peak torque values in the lower limbs  - No association found between muscle activation patterns and YBT-LQ performance in DCD. |

# **Table 11.** *Dual-Tasking*

| **Reference** | **Study Objectives** | **Participants**  *Age and/or Gender Matched* | **Referral method**  *School*  *Community*  *Clinical* | **DCD Screening Tool, Cut-Off.** | **Co-occurring disorders in sample**  *Y/N/Not Reported*  *(NR* | **DSM**  **Met**  Y/N | **Task(s) & Outcome measures** | **Key Results** (* significant) | **Effect Sizes** (key group effects) | **Quality Score**  (/10) | **Main Findings** |
| --- | --- | --- | --- | --- | --- | --- | --- | --- | --- | --- | --- |
| (Schott et al., 2016) | Examine the effect of concurrent cognitive task on fine and gross motor tasks in children with DCD. | N = 20 DCD  N = 39 TD  7-10Y  Age matched | School and Clinical | **BOTH:**  1. MABC-2 Test %tile <5 for DCD & >15^th^ TD  2. MABC-2 Checklist ‘problematic’  3. No medical, intellectual conditions | ID: N  ADHD: NR  ASD: NR | Y | *Dual task:*  Trail making and walking test (TMT/TWT) while attending to numbers and letters that increased.  Error rates and Dual Task Effects (DTE’s). | DTE interaction of condition x CMI*. Lower for overall motor interference for lower load condition.  DT condition of TMT error rates  DCD<TD  < Cog task complexity = > trail walking and tracing speed. | DTE:  *η_p_ ^2^* = 0.707*  (Simple) A/TWT-2  *d* = 0.260  (Complex) B/TWT-3:  *d* = 1.015* | 10 | - Higher motor interference in both motor tasks seen for DCD, but no cog interference differences  - Concurrent task performance resulted in increased task duration for both TD and DCD and walking duration change for DCD. |

# **Table 12.** *Attentional Focus and Motor Learning*

| **Reference** | **Study Objectives** | **Participants**  *Age and/or Gender Matched* | **Referral method**  *School*  *Community*  *Clinical* | **DCD Screening Tool, Cut-Off.** | **Co-occurring disorders in sample**  *Y/N/Not Reported*  *(NR* | **DSM**  **Met**  Y/N | **Task(s) & Outcome measures** | **Key Results** (* significant) | **Effect Sizes** (key group effects) | **Quality Score**  (/10) | **Main Findings** |
| --- | --- | --- | --- | --- | --- | --- | --- | --- | --- | --- | --- |
| (Adi-Japha and Brestel, 2020) | Comparison of motor skill learning DCD compared to TD peers on a letter-writing tasks. | N = 16 DCD  N = 16 TD  5-6Y  Age and nonverbal IQ matched | Community | **DCD**  1. Screened for ADHD, DLD & DCD  2. Occupational therapist referral and DCD diagnosis  3. MABC <16th %tile  **TD:**  1. No motor deficits  2. MABC >25th %tile  **Both**  1. Raven Matrices: Nonverbal IQ score >95  2. SES mid-high | ID: N  ADHD: N  ASD: NR | Y | Procedural learning task:  Invented letter task completed to assess time-dependent course of motor skill learning.  Dot-to-dot planar movements were completed to form letter progression.  Skill acquisition, far transfer and production time.  Spatiotemporal aspects of graphic reproduction. | Production time:  DCD > TD*  Dysfluency:  DCD > TD* | Production time:  d = 0.77  Dysfluency:  d = 0.76 | 8.5 | - DCD exhibited similar rates of learning to TD peers, however, overall poorer performance.  - DCD less fluent and took more time than TD peers  - DCD may require more guidance and individualised support when changing from dotted letter shapes to letter tracing |
| (de Carvalho et al., 2020) | Examine effects of high volume of practice by children with pDCD in relation to lower practice in TD | N = 8 TD  N = 8 pDCD  Gender and age matched  9-10Y | Unclear | **pDCD:**  1. MABC-2 <5th %tile  **TD:**  1. MABC-2 >25th %tile | ID: NR  ADHD: NR  ASD: NR | N | Active Wii video tennis or archery games: administered for 7 or 11 days  Tennis: strokes from 20 attempts  Archery: 12 shooting attempts | Volume of practice required:  pDCD > TD  Performance levels  pDCD<TD | Volume of practice required:  d = 2.07 | 3 | - pDCD improved performance as a function of practice, however, did not reach TD levels  - pDCD displayed a need for a higher volume of practice to increase performance compared to TD  - pDCD consistently lower performance levels than TD  -Motor difficulties can be minimised by training and practice. |
| (Khatab et al., 2018) | Investigate effects of internal and external focus of attention on dart throwing in DCD/TD. | N = 20 DCD  N = 20 TD Children  N = 20 TD Adults | University and School | **DCD:**  1. Persian motor observation questionnaire for screening. Children <16th %tile = DCD | ID: NR  ADHD: NR  ASD: NR | N | Throw darts to target under internal and external focus of attention (FOA)  Dart throwing scores under conditions. | Internal & external efficacy (FOA)  DCD < TD | **Accuracy**  Internal:  *d = 2.084*  External: *d* = 1.158 | 3.5 | - Adult performance was better than TD children and TD better than DCD  - DCD children exhibited feelings of frustration, thought to manifest as lack of focus and effort under challenging motor tasks.  - No significant difference between internal and external FOA in DCD. |
| (Li et al., 2019) | Determine the effects of different attentional focus conditions (external v internal v no) on postural stability in DCD | N = 91 DCD  N = 95 TD | School | **BOTH:**  1. KBIT-2 >80  2. Conners <70  3. No injuries/conditions  4. Right-handed  5. Normal vision  **DCD:**  MABC <5th %tile  **TD:**  MABC >50th %tile | ID: N  ADHD: N  ASD: NR | N | Pole-holding task and stand still parallel to the floor while focusing on pole movement (EF), hands (IF), or without instructions.  Aggregate pole movement and postural sway | Focus of attention main effect between pole movements and three focus conditions*  Pole mov - EF DCD<TDC*  Postural sway  DCD>TD | Main effects:  Pole displacement:  ηp 2 = 0.059  Poll roll rotation:  ηp 2 = 0.338 | 9 | - Instructions focusing on external focus of attention enhanced pole-holding and reduced postural sway in DCD and TD. |
| (Psotta et al., 2020) | Examine effects of attentional focus instructions on whole body coordination task performance in DCD v TD. | N = 18 DCD  N = 21 TD | ­School | **DCD:**  1. MABC-2 <15th %tile DCD and >15th %tile TD  2. MABC-2 Checklist | ID: NR  ADHD: NR  ASD: NR | Y | Counter movement vertical jumps x 3 performed under internal (IFA), external (EFA) and control conditions  Aggregate jump height and angular kinematics. | Jump height and take-off velocity  EFA>IFA*.  Group (DCD): main effect*  DCD<TD. | Height d = 0.190  Velocity  d = 0.320  Group  ηp 2 = .98 | 10 | - EFA and IFA instructions could enhance neuromuscular activation of dynamic contractions of the leg muscles for both TD and DCD  - External focus is more beneficial than internal focus to optimize whole-body motor performance |
| (Smits-Engelsman et al., 2020) | Examine impact of practice on motor skill acquisition, retention, and transfer in children with/without DCD. | N = 33 DCD  N = 28 TD | School | 1. MABC-2: <16th DCD and >25th %tile TD  2. No diagnosis/significant medical condition and absence of intellectual/cog impairment | ID: N  ADHD: NR  ASD: NR | Y | Wii ski-slalom game: to teach near transfer and motor skill learning  Wii score: no. gates missed, time to finish and training effect | Gates missed*  Time  DCD>TD  Training effect*  DCD<TD, yet improvements across both | Gates  d = 0.614  Time  d = 0.100  Training  d = 0.568 | 9.5 | - TD children appear more proficient in learning new skills compared to DCD.  - Demonstrates the possibility of a near transfer deficit in DCD |

# **Table 13.** *Neuroimaging*

| **Reference** | **Study Objectives** | **Participants**  *Age and/or Gender Matched* | **Referral method**  *School*  *Community*  *Clinical* | **DCD Screening Tool, Cut-Off.** | **Co-occurring disorders in sample**  *Y/N/Not Reported*  *(NR* | **DSM**  **Met**  Y/N | **Task(s) & Outcome measures** | **Key Results** (* significant) | **Effect Sizes** (key group effects) | **Quality Score**  (/10) | **Main Findings** |
| --- | --- | --- | --- | --- | --- | --- | --- | --- | --- | --- | --- |
| (Brown-Lum et al., 2020) | Characterise white matter differences using diffusion tensor imaging in children with and without DCD. | N = 31 TD  N = 31 DCD  8-12Y  Age and sex matched | Clinical and community | **DCD:**  1. MABC-2 <!6^th^ %tile  2. DCDQ <55 or <57 score  3. Parent reported MD and no other medical, intellectual conditions  3. Connors 3 <70 | ID: N  ADHD: N  ASD: NR | Y | **Diffusion tensor imaging:**  Diffusion parameters as an indirect measure of white matter microstructure  Fractional anistrophy, M and SD | Various white matter regions fractional anisotropy and axial diffusivity mean:  DCD < TD* | FA corticospinal tract:  *d* = 1.00  FA external capsule:  *d* = 1.181 | 3.5 | - Significant brain differences in motor and sensorimotor white matter pathways (CST, PTRs, cerebellar pathways, and splenium of the corpus callosum) in DCD compared to TD.  - Lower fractional anisotropy appears to be associated with lower axial diffusivity which suggests altered axonial development in DCD |
| (Cacola et al., 2018) | Investigate cortical activation using fNIRS in DCD. | N = 6 DCD  N = 6 TD  8-12Y | Clinic and community | 1. MABC-2 <5^th^ %tile (DCD)  2. DCDQ – daily functioning  3. Delay in motor milestones  4. No other medical conditions  5. KBIT-2 >Average | ID: NR  ADHD: NR  ASD: NR | Y | **Cortical activation during fine motor task:**  Finger tapping, curve tracing and paragraph writing while fNIRS measured cortical activation  (t-test comparison to baseline) | Motor performance  DCD<TD.  Cortical activation sig different between DCD and TD. | Not reported | 5 | - Neurological alterations in children with DCD  - Different activation patterns, even for FT tasks which require low fine motor control and no spatial or temporal demands |
| (Fong et al., 2016) | Comparison of motor performance and EEG attention levels between DCD and TD. | N = 57 DCD, Age 7.8±1.8  N = 99 TD, Age 7.4 ±1.6 | School &  clinical | **DCD:**  1. Diagnosis based on DSM-IV-TR  2. B-OTMP Test score <42 or <5% MABC.  **TD:**  Score >15% on MABC. | ID: NR  ADHD: NR  ASD: NR | Y | Prefrontal cortex EEG recording (Mind wave mobile headset) during MABC.  Item attention index during each MABC task (per/sec).  Index 0-100 = overall attention level during task. | Motor proficiency (TIS) DCD < TD  Attention levels  DCD < TD  TIS* correlated with total attention index | MP: *d* = 1.672  TAI:  *d* = 0.964  TIS and TAI:  *r =* -0.424 | 7 | - Children with DCD, regardless of ADHD comorbidity displayed lower attention during the MABC.  - Greater mental focus is associated with better motor performances on the MABC.  - Improvement in attention is particularly important to improve motor performance for children with DCD/ADHD |
| (He et al  ., 2018a) | Examine the integrity of interhemispheric cortical inhibition in motor cortices in DCD using transcranial magnetic stimulation (TMS). | N = 8 DCD  N= 10 TD  Age matched, range 18-30years | University | **DCD:**  1. Adult Dyspraxia/DCD checklist  2. BOT-2 total motor composite score (TMC) below 15th%tile  3. DSM-5 Criteria for DCD with Adults  ADC questionnaire to determine Criterion C of DSM-5.  **TD:**  Same screening criteria and BOT-2 TMC above 20th%tile. | ID: NR  ADHD: NR  ASD: NR | Y | Single-pulse TMS administered to the M1 of the brain.  Short-interval cortical inhibition (SICI)  Long-interval cortical inhibition (LICI)  Resting motor threshold (RMT)  Motor-evoked potential (MEP) | Baseline MEPs  Comparison of SICI, LICI and CSPratio  DCD=TD  ISPratio  DCD<TD*  ISPR & BOT-2 correlation | ISPRatio  d = 1.553  Correlation:  ηp 2 = 0.401 | 7.5 | - Contrary to expectations, intrahemispheric cortical inhibition at rest is preserved in DCD with no observed group differences.  - Reduced interhemispheric cortical inhibition in DCD  - May predict reduced motor performance for those with DCD. |
| (Hodgson and Hudson, 2017) | Assess left hemisphere lateralization for speech production in DCD v TD using fTCD | N = 12 TD  N = 12 DCD  Adults  Age and non-verbal ability matched | Community | **DCD:**  1.Self-reported diagnosis of DCD within last 10years  2. ADC checklist – sig motor difficulties criteria  **BOTH:**  No neurological disorder or medications affecting CNS  All completed higher education to 18y | ID: NR  ADHD: NR  ASD: NR | Y | Performed a word generation task whilst undergoing functional transcranial doppler (fTCD) while doing a peg-moving task.  Mean peg movement time and laterality indices (LI) | Laterality index (LI) score DCD<TD*  Mean peg movement times:  Non-pref hand*  DCD>TD | LI score: *d* = 0.410  Movement times: *d* = 0.920 | 8 | - Reduced asymmetry for speech and slower motor performance for DCD and provides evidence for links between motor control and language production  - Atypical cerebral lateralization occurs to due to a developmental impairment in either speech or motor control  - Slower non-preferred hand may demonstrate reduced strength of ipsilateral pathways for complex motor action. |
| (Hyde et al., 2018) | Examine the neural correlates (corticospinal excitability) of deficits in motor imagery (MI) in those with DCD. | N= 8 DCD  Age 20-33years  N = 21 TD  Age 18-36 years | University | **DCD:**  1. DSM-5 criteria for DCD in adults  2. BOT-2 scores at<15th%tile.  3. ADC below adult CI95%  **TD:**  1. BOT-2 >20th%tile  2. Free from medical/neurological impairment | ID: NR  ADHD: NR  ASD: NR | Y | MI ability assessed using HLT program.  Single-pulse TMS was delivered to a single hand that sat within response boxes.  Mean and accuracy of response time (RT) and inverse efficiency score (IES) | Mean IES*  Main group  MI during HLT  DCD<TD* | *Mean IES* *d = 1.154*  Group effect:  *η_p_ ^2^* = 0.23 | 8.5 | - Young adults with DCD who adopted MI were significantly less efficient than MI observed in TD.  - Those with DCD may experience difficulties generating internal action representations |
| (Hyde et al., 2019) | Characterise white matter tissue organisation of sensorimotor tracts in young adults with DCD. | N = 12 TD  N = 7 DCD  Age matched  18-46Y | University | **DCD:**  1. BOT-2 <16^th^ %tile: TD >20^th^ %tile  2. ADC CL95%  3. Exclusion of medical, neurological, and intellectual conditions | ID: N  ADHD: N  ASD: N | Y | **High angular diffusion MRI:**  Diffusion metrics | AFD on left SLF:  DCD < TD*  Tract volume on right SLF:  DCD < TD | AFD on left SLF:  *d* = 1.298  Tract volume on right SLF:  *d* = 0.742 | 4.5 | - Motor impairments characteristic of DCD may be subserved by white matter abnormalities in sensori-motor tracts, specifically left and right SLF |
| (Kashuk et al., 2017) | Investigate neural activation patterns in adults with pDCD compared to TD. | N = 12  pDCD  N = 11 TD  Adults 18-40 | University | **pDCD:**  1. MAND > 85  2. ADC Checklist  **TD:**  1. MAND <85  **BOTH**  1. Screening for ADHD, ID, ASD, Physical and neurological conditions**.** |  | N | Motor imagery:  Hand rotation task  Behav:  RTand accuracy  FMRI: BOLD signal activation % of occipito-parietal and parieto-frontal networks | Hard task:  RT and Accuracy pDCD=TD  BOLD activation change %:  PDCD=TD | RT: d = 0.657  Accuracy: d = 1.338  BOLD:  d = 0.222 | 7.5 | - No atypical performance pattern on motor imagery task in pDCD.  - Reduced pattern of activation supports theory of impairment and disruption within parieto-frontal and parieto-cerebellar networks |
| (Koch et al., 2018) | Dorsolateral prefrontal cortex (DLPFC) activation over time in children at risk of DCD using near-infared spectroscopy. | N = 10 rDCD  N = 11 TD  8-12Y | School | **BOTH:**  1. MABC-2: DCD %tile <10^th^ and TD >25^th^  2. KBIT-2 >90  3. Conners Parent <84^th^ %tile | ID: N  ADHD: N  ASD: NR | N | Children completed the stroop (response inhibition and speed) and Wisconsin card sort test (set-shifting and abstract reasoning).  Prefrontal activation was measured by OxyHB concentration changes. | Varied activation during activities.  TD: modulated DLPFC activity over time and only right lateralization during stroop task.  DCD: High sustained activation across hemispheres. | OxyHb concentration during WCST:  *d* = 0.669 | 2.5 | - DCD displayed sustained high activation of both left and right DLPFC during inhibition and se-shifting tasks compared to TD  - The continued high activation may be explained by perceptual and/or motor demands being more challenging in DCD |
| (Lust et al., 2019) | Investigate MNS function in children with DCD using EEG | N = 14 DCD  N = 14 TD  Age and sex matched | School and Clinical | **DCD:**  1. Formal DCD diagnosis  2. Re-ax of MABC <5th %tile  3. DCD-Q ‘DCD range’  4. IQ 75,80,96  **TD:**  1. MABC-2 >15th %tile  2. DCD-Q ‘no DCD’  3. IQ within normal range ‘mainstream education’ | ID: N  ADHD: NR  ASD: NR | Y | **Observational learning and detection**:  EEG testing  Mu suppression (Mu S): desynchronization of Mu  Mu coherence  (Mu C): direct corelate of mirror neuron activity | Task x group interaction for Mu S (Power)  DCD<TD*  Mu C: Observation and pause intervals DCD<TD* | Interaction:  *η_p_ ^2^* = 0.01  Mu C  *d*= 0.00 | 8 | - Clear MNS dysfunction in DCD in behaviour, mu power and mu coherence.  - Mu desynchronization reduced during observational learning and movement sequence imitation in DCD  - Task specific increase and decrease in Mu C during observation and pause intervals of the imitation task sig reduced in DCD. |
| (McLeod et al., 2016) | Examine functional connections of the left and right primary and sensory motor (SM1) cortices in children with DCD using resting state fMRI. | N = 6 DCD  N = 21 TD  Age matched | School | **DCD:**  1. MABC-2 <16^th^ %tile  2. DCD-Q  3. No visual impairment/conditions  4. No ID | ID: N  ADHD: NR  ASD: NR | Y | Functional images collected at resting state.  Connectivity maps | Stronger connections between right putamen and SMI in DCD. | Not reported | 2.5 | - Atypical within-and-between-hemisphere functional connection strength between SM1, regions of the basal ganglia and the cerebellum for DCD. |
| (Reynolds, Licari, et al., 2017a) | Assessed structural brain differences in grey matter (GM) volume in children with and without DCD. | N = 22 DCD  N = 22 TD  7-12Y | Clinical and community | **BOTH:**  1. MABC-2 %tile <16^th^ DCD and >20^th^ TD  2. Exclusion of ASD, ADHD | ID: NR  ADHD: N  ASD: N | N | Structural voxel-based morphology analysis performed.  GM volume | Right lateralised reduction in grey matter volume in medial and middle frontal and superior frontal gyri in DCD* | *d* = 0.608 | 5.5 | - GM volume in premotor frontal regions may underlie motor difficulties characteristic of DCD  - Focal differences in underlying brain structure may contribute to DCD and motor proficiency in general  - Possibility that neurorehabilitation intervention processes can increase GM volume |
| (Reynolds et al., 2019) | Examine brain activation patterns in children with and without DCD using finger tasks during MNS activation states. | N = 10 DCD  N = 9 TD  Age matched  8-13Y | Clinical | **DCD:**  1. MABC-2 <16th %tile  2. Exclusion of ADHD, ASD, Neurological conditions  **TD:**  1. MABC-2 >25th %tile  **BOTH**  1. EHI – Child version Right handedness | ID: NR  ADHD: N  ASD: N |  | Target-directed adduction/abduction index dinger tapping task. Under 1) action observation, 2) motor imagery, 3) action execution and 4) action imitation conditions.  Functional changes in blood-oxygen-level dependent (BOLD) signal across conditions. Region of interest (ROI) changes | Neurological  MNS activation  DCD = TD  R posterior parietal lobe group diff*  DCD<TD | Group:  *d = 0.982* | 9 | - No differences in MNS activation  - In non-mirror regions, clusters of decreased activation were seen: thalamus, caudate, and posterior cingulate.  - No support for MNS dysfunction theory  - Imagery deficits in DCD may stem from difficulties within the planning phase of movement, integration and updating of relevant visuospatial info rather than deficits in MNS. |
| (Rinat et al., 2020) | Compare whole-brain resting-state functional connectivity of children with DCD and TD. | N = 23 TD  N = 35 DCD+ADHD  8-12Y | Clinical and community | **BOTH:**  1. MABC-2 <16^th^ %tile (>25^th^ TD)  2. DCDQ suspect or indicative range  3. Young age MDs  4. No other medical conditions, ASD, ID, ADHD | ID: N  ADHD: N  ASD: N | Y | MRI scanning  at rest for 6minutes  Independent Component Analysis (ICA) of rsMRI data (t-tests)­ | DCD  Altered functional connectivity between sensorimotor network and PCC and pMTG | Sensorimotor network-PCC:  *d* = 1.31  Sensorimotor network-pMTG:  *d* = 1.21 | 7.5 | - PCC to prenucleus and pMTG areas of disrupted functional connectivity in DCD  - Ineffective communication between the sensorimotor network and PCC could indicate deficit in allocation of attentional and neural resources and may influence inefficient motor learning seen in DCD  - pMTG involvement indicate disruptions between mental representation of meaningful actions. |
| (Thornton et al., 2018) | Compared functional brain activation in children and adolescents with DCD compared to DCD/ADHD and TD. | N = 9 DCD  N = 20 TD  8-17Y | School and community | **BOTH:**  1. MABC-2 <16^th^ %tile DCD  2. DCDQ  3. No visual impairment, neurological conditions, or ID | ID: N  ADHD: NR  ASD: NR | Y | Motor response inhibition paradigm (Go/No-go task) with a frown or smile as ‘go’ cue.  Reaction times and interval between stimulus presentation and button pressing. Brain metrics | Go/No-go total errors:  DCD > TD*  BOLD activation patterns:  DCD = TD | Go/No-go total errors:  *d* = 0.763  BOLD activation patterns:  *d* = 0.274 | 8 | - Complex, global patterns of change in white matter and functional connectivity in DCD and ADHD  - Absence of a significant difference between isolated DCD and ADHD and combined highlights differences for co-occuring disorders |
| (Wang et al., 2017) | EEG oscillations associated with visuospatial working memory (VSWM) examined in DCD compared to TD | N = 29 DCD  N = 29 TD  10-11years | School | **DCD:**  1. MABC-2 <15th %tile  2. Behaviour ADHD screen  T**D:**  1. MABC-2 >20th %tile  **BOTH:**  1. No neurological, physical, or behavioural difficulties  2. WISC-R IQ>70 | ID: N  ADHD: N  ASD: NR | Y | **Visuospatial cognitive task**: Ladybug stimuli under delayed and non-delayed conditions  **Behavioural:** RTs, correct trials, and accuracy  **EEG:**  Time-frequency decompositions, alpha and theta activity. | Main effect of group:  RT and Accuracy  DCD<TD*  Theta increases & alpha DCD<TD | Acc: *η_p_ ^2^* = .22  RT:  *η_p_ ^2^* = .10 | 8 | - DCD lower accuracy and slower responses in delayed conditions  - Reduced theta increases during encoding and retrieval phases and less alpha suppression during late maintenance period  - Possible reduction in encoding and recognising new information (particularly task-relevant information) for DCD which result in poorer VSWM |
| (Williams et al., 2017) | Explore white matter (WM) integrity of adults with DCD/pDCD | N = 12 pDCD  N = 11 TD | University | **BOTH:**  1. MAND <85 motor impairment  2. Childhood motor difficulties  3. Exclusions of ADHD, ASD, ID, neurological or physical conditions | ID: N  ADHD: N  ASD: N | N | Lay supine throughout MR acquisition process  MRI metrics | FA (R) CST:  pDCD < TD*  FA (L) SLF:  pDCD < TD*    MD (L) IC:  pDCD < TD*  MD (R) ILF:  pDCD < TD* | FA (R) CST:  *d* = 0.432    FA (L) SLF:  *d* = 0.259    MD (L) IC:  *d* = 1.820    MD (R) ILF:  *d* = 1.599 | 10 | - Superior longitudinal fasciculus and lower mean diffusivity internal capsule and interior longitudinal fasciculus  - Reduced WM integrity in parietofrontal and corticospinal tracts with possible compensatory increases in WM integrity along visual ventral stream and front-occipital networks.  - Consistent with behavioural data showing constellation of deficits across motor planning, control, and cognition in DCD. |

**References**

Adams, I.L.J., Lust, J.M., Steenbergen, B., 2018. Development of motor imagery ability in children with developmental coordination disorder – A goal-directed pointing task. Br. J. Psychol. 109, 187–203. https://doi.org/10.1111/bjop.12274

Adams, I.L.J., Lust, J.M., Wilson, P.H., Steenbergen, B., 2017. Development of motor imagery and anticipatory action planning in children with developmental coordination disorder – A longitudinal approach. Hum. Mov. Sci. 55, 296–306. https://doi.org/10.1016/j.humov.2017.08.021

Adams, I.L.J., Lust, J.M., Wilson, P.H., Steenbergen, B., 2017a. Testing predictive control of movement in children with developmental coordination disorder using converging operations. Br. J. Psychol. 108, 73–90. https://doi.org/10.1111/bjop.12183

Adi-Japha, E., Brestel, G., 2020. Motor skill learning with impaired transfer by children with developmental coordination disorder. Res. Dev. Disabil. 103. https://doi.org/10.1016/j.ridd.2020.103671

Alesi, M., Gómez-López, M., Bianco, A., 2019. Motor differentiation’s and cognitive skill in pre-scholar age. Cuad. Psicol. Deporte 19, 50–59. https://doi.org/10.6018/cpd.338341

Alesi, M., Pecoraro, D., Pepi, A., 2019a. Executive functions in kindergarten children at risk for developmental coordination disorder. Eur. J. Spec. Needs Educ. 34, 285–296. https://doi.org/10.1080/08856257.2018.1468635

Barbacena, M.M., Valladao Novais Van Petten, A.M., Ferreira, D.L., Magalhaes, L. de C., 2019. Cognitive level and developmental coordination disorder: study with schoolchildren aged 7 to 10 years old. Braz. J. Occup. Ther. 27, 534–544. https://doi.org/10.4322/2526-8910.ctoAO1839

Bernardi, M., Leonard, H.C., Hill, E.L., Botting, N., Henry, L.A., 2018. Executive functions in children with developmental coordination disorder: a 2-year follow-up study. Dev. Med. Child Neurol. 60, 306–313. https://doi.org/10.1111/dmcn.13640

Bhoyroo, R., Hands, B., Wilmut, K., Hyde, C., Wigley, A., 2019. Motor planning with and without motor imagery in children with Developmental Coordination Disorder. Acta Psychol. (Amst.) 199. https://doi.org/10.1016/j.actpsy.2019.102902

Bhoyroo, R., Hands, B., Wilmut, K., Hyde, C., Wigley, A., 2018. Investigating motor planning in children with DCD: Evidence from simple and complex grip-selection tasks. Hum. Mov. Sci. 61, 42–51. https://doi.org/10.1016/j.humov.2018.07.006

Bieber, E., Smits-Engelsman, B.C.M., Sgandurra, G., Di Gregorio, F., Guzzetta, A., Cioni, G., Feys, H., Klingels, K., 2021. A new protocol for assessing action observation and imitation abilities in children with Developmental Coordination Disorder: A feasibility and reliability study. Hum. Mov. Sci. 75. https://doi.org/10.1016/j.humov.2020.102717

Blais, M., Amarantini, D., Albaret, J.-M., Chaix, Y., Tallet, J., 2018. Atypical inter-hemispheric communication correlates with altered motor inhibition during learning of a new bimanual coordination pattern in developmental coordination disorder. Dev. Sci. 21. https://doi.org/10.1111/desc.12563

Blais, M., Baly, C., Biotteau, M., Albaret, J.-M., Chaix, Y., Tallet, J., 2017. Lack of Motor Inhibition as a Marker of Learning Difficulties of Bimanual Coordination in Teenagers With Developmental Coordination Disorder. Dev. Neuropsychol. 42, 207–219. https://doi.org/10.1080/87565641.2017.1306526

Brown-Lum, M., Izadi-Najafabadi, S., Oberlander, T.F., Rauscher, A., Zwicker, J.G., 2020. Differences in white matter microstructure among children with developmental coordination disorder. Jama Netw. Open 3. https://doi.org/10.1001/jamanetworkopen.2020.1184

Cacola, P., Getchell, N., Srinivasan, D., Alexandrakis, G., Liu, H., 2018. Cortical activity in fine-motor tasks in children with Developmental Coordination Disorder: A preliminary fNIRS study. Int. J. Dev. Neurosci. 65, 83–90. https://doi.org/10.1016/j.ijdevneu.2017.11.001

Chen, F.-C., Li, L.-L., Chu, C.-H., Pan, C.-Y., Tsai, C.-L., 2019. Finger soaking enhances effects of light touch on reducing body sway in children with developmental coordination disorder. J. Rehabil. Med. 51, 217–224. https://doi.org/10.2340/16501977-2524

Chen, F.-C., Pan, C.-Y., Chu, C.-H., Tsai, C.-L., Tseng, Y.-T., 2020. Joint Position Sense of Lower Extremities is Impaired and Correlated with Balance Function in Children with Developmental Coordination Disorder. J. Rehabil. Med. 52, 1–9. https://doi.org/10.2340/16501977-2720

Cheng, Y.T.Y., Tsang, W.W.N., Schooling, C.M., Fong, S.S.M., 2018. Reactive balance performance and neuromuscular and cognitive responses to unpredictable balance perturbations in children with developmental coordination disorder. Gait Posture 62, 20–26. https://doi.org/10.1016/j.gaitpost.2018.02.025

Cignetti, F., Vaugoyeau, M., Fontan, A., Jover, M., Livet, M.-O., Hugonenq, C., Audic, F., Chabrol, B., Assaiante, C., 2018. Feedforward motor control in developmental dyslexia and developmental coordination disorder: Does comorbidity matter? Res. Dev. Disabil. 76, 25–34. https://doi.org/10.1016/j.ridd.2018.03.001

Costini, O., Roy, A., Faure, S., Remigereau, C., Renaud, E., Blanvillain, L., Fossoud, C., Le Gall, D., 2018. Gestures and related skills in developmental coordination disorder: A production-system deficit? Psychol. Neurosci. 11, 193–215. https://doi.org/10.1037/pne0000115

da Rocha Diz, M.A., Ferracioli, M. de C., Hiraga, C.Y., de Oliveira, M.A., Pellegrini, A.M., 2018. Effects of practice on visual finger-force control in children at risk of developmental coordination disorder. Braz. J. Phys. Ther. 22, 467–473. https://doi.org/10.1016/j.bjpt.2018.04.002

de Carvalho, L.S., Crancianinov, C.S.A., Gama, D.T., Hiraga, C.Y., 2020. Effect of volume of practice in children with probable developmental coordination disorder. Rev. Bras. Cineantropometria E Desempenho Hum. 22, 1–10. https://doi.org/10.1590/1980-0037.2020v22e72028

de Waal, E., Pienaar, A.E., Coetzee, D., 2018. Gender differences in academic achievement of children with developmental coordination disorder. SOUTH Afr. J. Child. Educ. https://doi.org/10.4102/sajce.v8i1.515

Fong, S.S.M., Chung, J.W.Y., Cheng, Y.T.Y., Yam, T.T.T., Chiu, H.-C., Fong, D.Y.T., Cheung, C.Y., Yuen, L., Yu, E.Y.T., Hung, Y.S., Macfarlane, D.J., Ng, S.S.M., 2016. Attention during functional tasks is associated with motor performance in children with developmental coordination disorder: A cross-sectional study. Medicine (Baltimore) 95. https://doi.org/10.1097/MD.0000000000004935

Fuchs, C.T., Cacola, P., 2018. Differences in accuracy and vividness of motor imagery in children with and without Developmental Coordination Disorder. Hum. Mov. Sci. 60, 234–241. https://doi.org/10.1016/j.humov.2018.06.015

Gama, D.T., Ferracioli, M.D.C., Hiraga, C.Y., Pellegrini, A.M., 2016. Value of pre-cue information for motor tasks performed by children with developmental coordination disorder (DCD). Mot. Rev. Educ. Fis. 22, 138–143. https://doi.org/10.1590/S1980-6574201600030004

Ganapathy Sankar, U., Monisha, R., 2019. Assessment of balance in children with developmental coordination disorder in Indian context. Indian J. Public Health Res. Dev. 10, 67–70. https://doi.org/10.5958/0976-5506.2019.01538.9

Gauthier, S., Anzalone, S.M., Cohen, D., Zaoui, M., Chetouani, M., Villa, F., Berthoz, A., Xavier, J., 2018. Behavioral Own-Body-Transformations in Children and Adolescents With Typical Development, Autism Spectrum Disorder, and Developmental Coordination Disorder. Front. Psychol. 9. https://doi.org/10.3389/fpsyg.2018.00676

Gaymard, B., Giannitelli, M., Challes, G., Rivaud-Pechoux, S., Bonnot, O., Cohen, D., Xavier, J., 2017. Oculomotor Impairments in Developmental Dyspraxia. Cerebellum 16, 411–420. https://doi.org/10.1007/s12311-016-0817-6

Gentle, J., Barnett, A.L., Wilmut, K., 2016. Adaptations to walking on an uneven terrain for individuals with and without Developmental Coordination Disorder. Hum. Mov. Sci. 49, 346–353. https://doi.org/10.1016/j.humov.2016.08.010

Ghotbi, M., Sohrabi, M., Taheri, H.R., Khodashenas, E., 2016. The comparison of depth perception in 7-9 years old healthy children with developmental coordination disorder. J. Ecophysiol. Occup. Health 16, 138–143. https://doi.org/10.15512/joeoh/2016/v16i3&4/15461

Golenia, L., Bongers, R.M., van Hoorn, J.F., Otten, E., Mouton, L.J., Schoemaker, M.M., 2018. Variability in coordination patterns in children with developmental coordination disorder (DCD). Hum. Mov Sci. https://doi.org/10.1016/j.humov.2018.06.009

Gomez-Moya, R., Diaz, R., Vaca-Palomares, I., Fernandez-Ruiz, J., 2020. Procedural and strategic visuomotor learning deficits in children with developmental coordination disorder. Res. Q. Exerc. Sport. https://doi.org/10.1080/02701367.2019.1675852

Gonzalez, C.C., Mon-Williams, M., Burke, S., Burke, M.R., 2016. Cognitive control of saccadic eye movements in children with developmental coordination disorder. PloS One. https://doi.org/10.1371/journal.pone.0165380

He, J.L., Fuelscher, I., Coxon, J., Barhoun, P., Parmar, D., Enticott, P.G., Hyde, C., 2018. Impaired motor inhibition in developmental coordination disorder. Brain Cogn. 127, 23–33. <https://doi.org/10.1016/j.bandc.2018.09.002>

He, J.L., Fuelscher, I., Enticott, P.G., Teo, W., Barhoun, P., Hyde, C., 2018a. Interhemispheric cortical inhibition is reduced in young adults with developmental coordination disorder. Front. Neurol. 9. https://doi.org/10.3389/fneur.2018.00179

Hodgson, J.C., Hudson, J.M., 2017. Atypical speech lateralization in adults with developmental coordination disorder demonstrated using functional transcranial Doppler ultrasound. J. Neuropsychol. 11, 1–13. https://doi.org/10.1111/jnp.12102

Hsu, L.-Y., Jirikowic, T., Ciol, M.A., Clark, M., Kartin, D., McCoy, S.W., 2018. Motor planning and gait coordination assessments for children with developmental coordination disorder. Phys. Occup. Ther. Pediatr. 38, 562–574. https://doi.org/10.1080/01942638.2018.1477226

Hyde, C., Fuelscher, I., Williams, J., Lum, J.A.G., He, J., Barhoun, P., Enticott, P.G., 2018. Corticospinal excitability during motor imagery is reduced in young adults with developmental coordination disorder. Res. Dev. Disabil. 72, 214–224. https://doi.org/10.1016/j.ridd.2017.11.009

Hyde, C., Ian, F., Peter, E.G., Derek, J.K., Shawna, F., Tim, S.J., Jacqueline, W., Karen, C., 2019. White matter organization in developmental coordination disorder: A pilot study exploring the added value of constrained spherical deconvolution. Neuroimage-Clin. 21. https://doi.org/10.1016/j.nicl.2018.101625

Jelsma, L.D., Geuze, R.H., Smits-Engelsman, B.C.M., 2020. Movement control strategies in a dynamic balance task in children with and without developmental coordination disorder. J. Mot. Behav. 52, 175–186. https://doi.org/10.1080/00222895.2019.1599809

Job, X.E., Bradya, D., de Fockert, J.W., Luft, C.D.B., Hill, E.L., van Velzen, J., 2019. Adults with probable developmental coordination disorder selectively process early visual, but not tactile information during action preparation. An electrophysiological study. Hum. Mov. Sci. 66, 631–644. https://doi.org/10.1016/j.humov.2019.02.018

Johnston, J.S., Ali, J.B., Hill, E.L., Bremner, A.J., 2017. Tactile localization performance in children with developmental coordination disorder (DCD) corresponds to their motor skill and not their cognitive ability. Hum. Mov. Sci. 53, 72–83. https://doi.org/10.1016/j.humov.2016.12.008

Kashuk, S.R., Williams, J., Thorpe, G., Wilson, P.H., Egan, G.F., 2017. Diminished motor imagery capability in adults with motor impairment: An fMRI mental rotation study. Behav. Brain Res. 334, 86–96. https://doi.org/10.1016/j.bbr.2017.06.042

Ke, L., Duan, W., Xue, Y., Wang, Y., 2019. Developmental Coordination Disorder in Chinese Children Is Correlated With Cognitive Deficits. Front. PSYCHIATRY. https://doi.org/10.3389/fpsyt.2019.00404

Khatab, S.F., Ghasemi, A., Sadati, S.K.M., 2018. The Effect of Focus Instructions on Dart Throwing Performance in Children With and Without Developmental Coordination Disorder. Ann. Appl. Sport Sci. 6, 55–60.

Koch, J.K.L., Miguel, H., Smiley-Oyen, A.L., 2018. Prefrontal activation during Stroop and Wisconsin card sort tasks in children with developmental coordination disorder: a NIRS study. Exp. Brain Res. 236, 3053–3064. https://doi.org/10.1007/s00221-018-5358-4

Krajenbrink, H., Lust, J.M., Steenbergen, B., 2021. Eliciting End-State Comfort Planning in Children With and Without Developmental Coordination Disorder Using a Hammer Task: A Pilot Study. Front. Psychol. 12. https://doi.org/10.3389/fpsyg.2021.625577

Lê, M., Blais, M., Jucla, M., Chauveau, N., Maziero, S., Biotteau, M., Albaret, J., Péran, P., Chaix, Y., Tallet, J., 2021. Procedural learning and retention of audio‐verbal temporal sequence is altered in children with developmental coordination disorder but cortical thickness matters. Dev. Sci. 24, 1–14. https://doi.org/10.1111/desc.13009

Li, L.-L., Li, Y.-C., Chu, C.-H., Pan, C.-Y., Chen, F.-C., 2019. External focus of attention concurrently elicits optimal performance of suprapostural pole-holding task and postural stability in children with developmental coordination disorder. Neurosci. Lett. 703, 32–37. https://doi.org/10.1016/j.neulet.2019.03.011

Licari, M.K., Reynolds, J.E., Tidman, S., Ndiaye, S., Sekaran, S.N., Reid, S.L., Lay, B.S., 2018. Visual tracking behaviour of two-handed catching in boys with developmental coordination disorder. Res. Dev. Disabil. 83, 280–286. https://doi.org/10.1016/j.ridd.2018.07.005

Lust, J.M., van Schie, H.T., Wilson, P.H., van der Helden, J., Pelzer, B., Steenbergen, B., 2019. Activation of mirror neuron regions is altered in Developmental Coordination Disorder (DCD)–neurophysiological evidence using an action observation paradigm. Front. Hum. Neurosci. 13. https://doi.org/10.3389/fnhum.2019.00232

Mannini, A., Martinez-Manzanera, O., Lawerman, T.F., Trojaniello, D., Della Croce, U., Sival, D.A., Maurits, N.M., Sabatini, A.M., 2017. Automatic classification of gait in children with early-onset ataxia or developmental coordination disorder and controls using inertial sensors. Gait Posture 52, 287–292. https://doi.org/10.1016/j.gaitpost.2016.12.002

McLeod, K.R., Langevin, L.M., Dewey, D., Goodyear, B.G., 2016. Atypical within-and between-hemisphere motor network functional connections in children with developmental coordination disorder and attention-deficit/hyperactivity disorder. Neuroimage-Clin. 12, 157–164. https://doi.org/10.1016/j.nicl.2016.06.019

Michel, E., Molitor S., Schneider W., 2018. Differential changes in the development of motor coordination and executive functions in children with motor coordination impairments. Child Neuropsychol. 24, 20–45. https://doi.org/10.1080/09297049.2016.1223282

Miller, H.L., Cacola, P.M., Sherrod, G.M., Patterson, R.M., Bugnariu, N.L., 2019. Children with Autism Spectrum Disorder, Developmental Coordination Disorder, and typical development differ in characteristics of dynamic postural control: A preliminary study. Gait Posture 67, 9–11. https://doi.org/10.1016/j.gaitpost.2018.08.038

Mirabella, G., Del Signore, S., Lakens, D., Averna, R., Penge, R., Capozzi, F., 2017. Developmental coordination disorder affects the processing of action-related verbs. Front. Hum. Neurosci. 10. https://doi.org/10.3389/fnhum.2016.00661

Nieto, M.P., Valtr, L., Abdollahipour, R., Agricola, A., Psotta, R., 2018. The role of vision in walking patterns in children with different levels of motor coordination. Rev. Iberoam. Psicol. Ejerc. El Deporte 13, 289–296.

Nobusako, S., Osumi, M., Furukawa, E., Nakai, A., Maeda, T., Morioka, S., 2021. Increased visual bias in children with developmental coordination disorder: Evidence from a visual-tactile temporal order judgment task. Hum. Mov. Sci. 75, 102743. https://doi.org/10.1016/j.humov.2020.102743

Nobusako, S., Sakai, A., Tsujimoto, T., Shuto, T., Nishi, Y., Asano, D., Furukawa, E., Zama, T., Osumi, M., Shimada, S., Morioka, S., Nakai, A., 2018. Deficits in visuo-motor temporal integration impacts manual dexterity in probable developmental coordination disorder. Front. Neurol. 9. https://doi.org/10.3389/fneur.2018.00114

Nunzi, M., Sylos Labini, F., Meli, A., Baldi, S., Tufarelli, D., Di Brina, C., 2018. Static balance performance and sensory integration abilities of children with dyslexia and developmental coordination disorder, in: da Silva H.P., Constantine L., Escalona M.J., Ramirez A.J., Helfert M. (Eds.), CHIRA - Proc. Int. Conf. Computer-Human Interact. Res. Appl. SciTePress, pp. 150–155. https://doi.org/10.5220/0006930601500155

Opitz, B., Brady, D., Leonard, H.C., 2020. Motor and non-motor sequence prediction is equally affected in children with developmental coordination disorder. PloS One 15, e0232562. https://doi.org/10.1371/journal.pone.0232562

Parr, J.V.V., Foster, R.J., Wood, G., Hollands, M.A., 2020. Children with developmental coordination disorder exhibit greater stepping error despite similar gaze patterns and state anxiety levels to their typically developing peers. Front. Hum. Neurosci. 14. https://doi.org/10.3389/fnhum.2020.00303

Parr, J.V.V., Foster, R.J., Wood, G., Thomas, N.M., Hollands, M.A., 2020a. Children with developmental coordination disorder show altered visuomotor control during stair negotiation associated with heightened state anxiety. Front. Hum. Neurosci. 14. https://doi.org/10.3389/fnhum.2020.589502

Prunty, M., Barnett, A.L., Wilmut, K., Plumb, M., 2016. Visual perceptual and handwriting skills in children with Developmental Coordination Disorder. Hum. Mov. Sci. 49, 54–65. https://doi.org/10.1016/j.humov.2016.06.003

Psotta, R., Abdollahipour, R., Janura, M., 2020. The Effects of Attentional Focus Instruction on the Performance of a Whole-Body Coordination Task in Children With Developmental Coordination Disorder. Res. Dev. Disabil. 101. https://doi.org/10.1016/j.ridd.2020.103654

Purcell, C., Wilmut, K., Wann, J.P., 2017. The use of visually guided behaviour in children with Developmental Coordination Disorder (DCD) when crossing a virtual road. Hum. Mov. Sci. 53, 37–44. https://doi.org/10.1016/j.humov.2016.11.007

Rahimi-Golkhandan, S., Steenbergen, B., Piek, J.P., Caeyenberghs, K., Wilson, P.H., 2016. Revealing hot executive function in children with motor coordination problems: What’s the go? Brain Cogn. 106, 55–64. https://doi.org/10.1016/j.bandc.2016.04.010

Reynolds, J.E., Billington, J., Kerrigan, S., Williams, J., Elliott, C., Winsor, A.M., Codd, L., Bynevelt, M., Licari, M.K., 2019. Mirror neuron system activation in children with developmental coordination disorder: A replication functional MRI study. Res. Dev. Disabil. 84, 16–27. https://doi.org/10.1016/j.ridd.2017.11.012

Reynolds, J.E., Kerrigan, S., Elliott, C., Lay, B.S., Licari, M.K., 2017. Poor imitative performance of unlearned gestures in children with probable developmental coordination disorder. J. Mot. Behav. 49, 378–387. https://doi.org/10.1080/00222895.2016.1219305

Reynolds, J.E., Licari, M.K., Reid, S.L., Elliott, C., Winsor, A.M., Bynevelt, M., Billington, J., 2017a. Reduced relative volume in motor and attention regions in developmental coordination disorder: A voxel-based morphometry study. Int. J. Dev. Neurosci. 58, 59–64. https://doi.org/10.1016/j.ijdevneu.2017.01.008

Rinat, S., Izadi-Najafabadi, S., Zwicker, J.G., 2020. Children with developmental coordination disorder show altered functional connectivity compared to peers. NeuroImage Clin. 27. https://doi.org/10.1016/j.nicl.2020.102309

Roche, R., Viswanathan, P., Clark, J.E., Whitall, J., 2016. Children with developmental coordination disorder (DCD) can adapt to perceptible and subliminal rhythm changes but are more variable. Hum. Mov. Sci. 50, 19–29. https://doi.org/10.1016/j.humov.2016.09.003

Sartori, R.F., Valentini, N.C., Fonseca, R.P., 2020. Executive function in children with and without developmental coordination disorder: A comparative study. Child Care Health Dev. 46, 294–302. https://doi.org/10.1111/cch.12734

Schott, N., El-Rajab, I., Klotzbier, T., 2016. Cognitive-motor interference during fine and gross motor tasks in children with Developmental Coordination Disorder (DCD). Res. Dev. Disabil. 57, 136–148. https://doi.org/10.1016/j.ridd.2016.07.003

Scott, M.W., Emerson, J.R., Dixon, J., Tayler, M.A., Eaves, D.L., 2020. Motor imagery during action observation enhances imitation of everyday rhythmical actions in children with and without developmental coordination disorder. Hum. Mov. Sci. 71. https://doi.org/10.1016/j.humov.2020.102620

Scott, M.W., Emerson, J.R., Dixon, J., Tayler, M.A., Eaves, D.L., 2019. Motor imagery during action observation enhances automatic imitation in children with and without developmental coordination disorder. J. Exp. Child Psychol. 183, 242–260. https://doi.org/10.1016/j.jecp.2019.03.001

Smits-Engelsman, B., Bonney, E., Ferguson, G., 2020. Motor skill learning in children with and without Developmental Coordination Disorder. Hum. Mov. Sci. 74. https://doi.org/10.1016/j.humov.2020.102687

Speedtsberg, M.B., Christensen, S.B., Andersen, K.K., Bencke, J., Jensen, B.R., Curtis, D.J., 2017. Impaired postural control in children with developmental coordination disorder is related to less efficient central as well as peripheral control. Gait Posture 51, 1–6. https://doi.org/10.1016/j.gaitpost.2016.09.019

Speedtsberg, M.B., Christensen, S.B., Stenum, J., Kallemose, T., Bencke, J., Curtis, D.J., Jensen, B.R., 2018. Local dynamic stability during treadmill walking can detect children with developmental coordination disorder. Gait Posture 59, 99–103. https://doi.org/10.1016/j.gaitpost.2017.09.035

Sumner, E., Hutton, S.B., Kuhn, G., Hill, E.L., 2018a. Oculomotor atypicalities in Developmental Coordination Disorder. Dev. Sci. 21. https://doi.org/10.1111/desc.12501

Sumner, E., Pratt, M.L., Hill, E.L., 2016. Examining the cognitive profile of children with Developmental Coordination Disorder. Res. Dev. Disabil. 56, 10–17. https://doi.org/10.1016/j.ridd.2016.05.012

Suzuki, K., Kita, Y., Shirakawa, Y., Egashira, Y., Mitsuhashi, S., Kitamura, Y., Okuzumi, H., Kaga, Y., Inagaki, M., 2020. Reduced Nogo-P3 in adults with developmental coordination disorder (DCD). Int. J. Psychophysiol. 153, 37–44. https://doi.org/10.1016/j.ijpsycho.2020.04.009

Thornton, S., Bray, S., Langevin, L.M., Dewey, D., 2018. Functional brain correlates of motor response inhibition in children with developmental coordination disorder and attention deficit/hyperactivity disorder. Hum. Mov. Sci. 59, 134–142. https://doi.org/10.1016/j.humov.2018.03.018

Tseng, Y.-T., Holst-Wolf, J.M., Tsai, C.-L., Chen, F.-C., Konczak, J., 2019. Haptic perception is altered in children with developmental coordination disorder. Neuropsychologia 127, 29–34. https://doi.org/10.1016/j.neuropsychologia.2019.02.004

Tseng, Y.-T., Tsai, C.-L., Chen, F.-C., Konczak, J., 2018. Wrist position sense acuity and its relation to motor dysfunction in children with developmental coordination disorder. Neurosci. Lett. 674, 106–111. https://doi.org/10.1016/j.neulet.2018.03.031

Tseng, Y.-T., Tsai, C.-L., Chen, F.-C., Konczak, J., 2019a. Position Sense Dysfunction Affects Proximal and Distal Arm Joints in Children with Developmental Coordination Disorder. J. Mot. Behav. 51, 49–58. https://doi.org/10.1080/00222895.2017.1415200

Wang, C.-H., Tseng, Y.-T., Liu, D., Tsai, C.-L., 2017. Neural oscillation reveals deficits in visuospatial working memory in children with developmental coordination disorder. Child Dev. 88, 1716–1726. https://doi.org/10.1111/cdev.12708

Warlop, G., Vansteenkiste, P., Lenoir, M., Deconinck, F.J.A., 2020a. An exploratory study of gaze behaviour in young adults with developmental coordination disorder. Hum. Mov. Sci. 73. https://doi.org/10.1016/j.humov.2020.102656

Warlop, G., Vansteenkiste, P., Lenoir, M., Van Causenbroeck, J., Deconinck, F.J.A., 2020. Gaze behaviour during walking in young adults with developmental coordination disorder. Hum. Mov. Sci. 71. https://doi.org/10.1016/j.humov.2020.102616

Wattad, R., Gabis, L., V., Shefer, S., Tresser, S., Portnoy, S., 2020. Correlations between performance in a virtual reality game and the movement assessment battery diagnostics in children with developmental coordination disorder. Appl. Sci.-Basel 10. https://doi.org/10.3390/app10030833

Williams, J., Kashuk, S.R., Wilson, P.H., Thorpe, G., Egan, G.F., 2017. White matter alterations in adults with probable developmental coordination disorder: an MRI diffusion tensor imaging study. NeuroReport 28, 87–92. https://doi.org/10.1097/WNR.0000000000000711

Wilmut, K., Barnett, A.L., 2017a. When an object appears unexpectedly: anticipatory movement and object circumvention in individuals with and without Developmental Coordination Disorder. Exp. Brain Res. 235, 1531–1540. https://doi.org/10.1007/s00221-017-4901-z

Wilmut, K., Barnett, A.L., 2017b. When an object appears unexpectedly: foot placement during obstacle circumvention in children and adults with developmental coordination disorder. Exp. Brain Res. 235, 2947–2958. https://doi.org/10.1007/s00221-017-5031-3

Wilmut, K., Du, W., Barnett, A.L., 2017. Navigating through apertures: perceptual judgements and actions of children with Developmental Coordination Disorder. Dev. Sci. 20. https://doi.org/10.1111/desc.12462

Wilmut, K., Gentle, J., Barnett, A.L., 2017c. Gait symmetry in individuals with and without developmental coordination disorder. Res. Dev. Disabil. 60, 107–114. https://doi.org/10.1016/j.ridd.2016.11.016

Wilson, P., Ruddock S., Rahimi-Golkhandan S., Piek J., Sugden D., Green D., Steenbergen B., 2020. Cognitive and motor function in developmental coordination disorder. Dev. Med. Child Neurol. https://doi.org/10.1111/dmcn.14646

Yam, T.T.T., Fong, S.S.M., 2019. Y-balance test performance and leg muscle activations of children with developmental coordination disorder. J. Mot. Behav. 51, 385–393. https://doi.org/10.1080/00222895.2018.1485011

Yam, T.T.T., Fong, S.S.M., 2018. Leg muscle activation patterns during walking and leg lean mass are different in children with and without developmental coordination disorder. Res. Dev. Disabil. 73, 87–95. https://doi.org/10.1016/j.ridd.2017.12.014
