# Supplementary material for: Behavioral and Neuroimaging Research on Developmental Coordination Disorder (DCD): A Combined Systematic Review and Meta-Analysis of Recent Findings
Source: Front Psychol. 2022 Jan 27;13:809455. doi: 10.3389/fpsyg.2022.809455 (PMC8829815; doi:10.3389/fpsyg.2022.809455)
Supplement: Supplementary file 1 [file Data_Sheet_1.zip › Supplementary Material - Data Sheet 1/Supplementary Material 3.docx]

**Supplementary Material 3**

Summary of performance categories (and subcategories), their main measures, and contributing studies.

| **Coded categories** | **Main measures** | **Contributing studies** |
| --- | --- | --- |
| **1 Cognitivist Approach** | | |
| **1.1 Basic visual functions** |  |  |
| - Depth perception | Depth perception score | (Ghotbi et al., 2016) |
| **1.2 Visuo-perceptual processing** |  |  |
| No motor involvement |  |  |
| *Object perception* |  |  |
| - Object identification - Matching patterns - Non-delayed location matching - Visual discrimination of apertures | Object naming time  Pattern matching and association  Response accuracy and time  Visual discrimination; TVPS | (Alesi et al., 2019; Costini et al., 2018; Prunty et al., 2016; Wang et al., 2017) |
| *Spatial integration* |  |  |
| - Spatial integration/relations | NEPSY arrows | (Costini et al., 2018) |
| Motor involvement |  |  |
| - Visual-motor integration | VMI (e.g., Beery); ROF | (Adi-Japha and Brestel, 2020; Cacola et al., 2018; Costini et al., 2018; de Waal et al., 2018; Prunty et al., 2016) |
| **1.3 Kinaesthetic perception** |  |  |
| Proximal and distal |  |  |
| - Position reproduction task (passive model) - Position sense acuity: Wrist and elbow sensitivity; contralateral & ipsilateral matching - Position sense acuity under passive movement | Position sense error (or *Bias*); position sense error variability (or *Precision*); position sense thresholds (deg) | (Chen et al., 2020; Tseng et al., 2018, 2019a) |
| **1.4 Tactile and Haptic perception** |  |  |
| - Tactual Performance Test - Visuo-Tactile Temporal Order Judgement Task - Spatial Tactile Localisation - Haptic detection and acuity | Pressure  Point of subjective equality  Accuracy of localisation  Detection threshold; discrimination threshold | (Chen et al., 2019; Johnston et al., 2017; Nobusako et al., 2021; Tseng et al., 2019) |
| **1.5 Executive function** |  |  |
| Inhibitory Control |  |  |
| - Action Restraint: Go/NoGo (auditory-motor, visual-motor, Hayling test, delayed reaching task, semantic task, colour discrimination) | Response time; movement time; errors (no. or %); discrimination index; action restraint efficiency index | (He et al., 2018; Job et al., 2019; Michel et al., 2018; Mirabella et al., 2017; Sartori et al., 2020; Suzuki et al., 2020; Thornton et al., 2018) |
| - Action Cancellation: Stop-signal | Stop-signal reaction time; lift time; absolute and variable error | (He et al., 2018) |
| - Interference Control: Stroop task, animal Stroop, five-digit test, HTKS task | Response time; errors; total score | (Adi-Japha and Brestel, 2020; Alesi et al., 2019; Costini et al., 2018; Koch et al., 2018; Sartori et al., 2020) |
| - Jump Reaching Task and others: VIMI, HTKS task, double step reaching | Movement time; errors; path length | (Adams et al., 2017a; Bernardi et al., 2018; Michel et al., 2018) |
| Working Memory |  |  |
| - Visual (pathway recall, semi-circles, colour span, Corsi-Blocks, odd-one-out) - Verbal (Word recall, word span, number recall) | Correct responses; errors; longest sequence; reaction time  No. correct repetitions; longest sequence | (Adi-Japha & Brestel, 2020; Alesi, Gómez-López, et al., 2019; Alesi, Pecoraro, et al., 2019; Michel et al., 2018; Sartori et al., 2020; Wang et al., 2017) |
| Executive attention |  |  |
| - Task / Set Shifting (animal fluency, colours fluency, fruits fluency, toys fluency, verbal fluency, design fluency, listening recall, sorting, trail making, flanker task, HTKS task, GMLT, WCST-64) | No. words; correct trials; completion time; errors; reaction time | (Alesi, Gómez-López, et al., 2019; Bernardi et al., 2018; Koch et al., 2018; Michel et al., 2018; Sartori et al., 2020; Schott et al., 2016; Wilson et al., 2020) |
| Hot EF |  |  |
| - Gift Wrap Task, Snack Delay Task, Emotional Go/No Go | No. peeking behaviours; period of restraint; errors; reaction time | (Alesi et al., 2019; Rahimi-Golkhandan et al., 2016) |
| **1.6 Intelligence Factors** |  |  |
| - Full Scale IQ and subscales | WISC Full Scale and factors; Raven’s Progressive Matrix; Raven’s Coloured Progressive Matrix; NEPSY Comprehension & Verbal Fluency | (Adi-Japha and Brestel, 2020; Barbacena et al., 2019; Costini et al., 2018; Gomez-Moya et al., 2020; Johnston et al., 2017; Sumner et al., 2016) |
|  |  |  |
| **2 Dynamical / Ecological Paradigms** | | |
| **2.1 Rhythmic coordination and timing** |  |  |
| Visuo-Manual coupling |  |  |
| - Coupling/accuracy | Relative phase; relative phase SD; additional taps | (Blais et al., 2018, 2017) |
| Auditory-Manual coupling |  |  |
| - Coupling/accuracy | Relative phase; relative phase SD | (Roche et al., 2016) |
| Auditory-Verbal coupling |  |  |
| - Coupling/accuracy - Stability - Errors | Vector angle  Vector length  No. errors | (Lê et al., 2021) |
| Visual-Verbal coupling |  |  |
| - Coupling/accuracy - Stability - Errors | Vector angle  Vector length  No. errors | (Lê et al., 2021) |
| Auditory-Visual-Verbal coupling |  |  |
| - Coupling/accuracy - Stability - Errors | Vector angle  Vector length  No. errors | (Lê et al., 2021) |
| **2.2 Ecological perception** |  |  |
| Time to contact |  |  |
| - Virtual traffic crossing | Temporal gap acceptance threshold | (Purcell et al., 2017) |
| Perception of affordances |  |  |
| - Visual estimation task - Perceptual Action Judgement - Aperture traversing | Absolute error; constant error  Absolute error; constant error; critical ratio  Critical ratio; approach speed; lateral trunk movement; baseline yaw; % change in speed; shoulder angle | (Wilmut et al., 2017) |
|  |  |  |
| **3 Cognitive Neuroscience & Hybrid** | | |
| **3.1 Motor imagery** |  |  |
| Explicit motor imagery |  |  |
| - Objective task - Subjective task | C-VRFT  FPIQ; MIQ-C | (Adams et al., 2018; Fuchs and Cacola, 2018; Scott et al., 2019) |
| Implicit motor imagery |  |  |
| - Mental limb rotation | Response time; accuracy; reaction time; no. errors | (Adams et al., 2017, 2017a; Hyde et al., 2018; Kashuk et al., 2017) |
| **3.2 Action observation, imitation, and gesture production** | |  |
| Imitation of simple gestures |  |  |
| - AO alone - AO + MI instruction - Imitation | Mean cycle time ratio %; no. errors | (Lust et al., 2019; Scott et al., 2019) |
| Imitation of everyday actions |  |  |
| - Imitation - Imitation with AO and MI - Imitation with MI | Mean cycle time ratio % | (Scott et al., 2020) |
| Imitation of complex novel gestures |  |  |
| - Sequence gestures (hand, arm, fingers) - Non-representational gestures | Correct response %; total score | (Bieber et al., 2021; Costini et al., 2018; Reynolds et al., 2017) |
| Action imitation |  |  |
| - Perspective taking, tightrope walker paradigm | No. tagged movements; movement amplitude; ego- and hetero-movement latency; movement length | (Gauthier et al., 2018) |
| Observation and imitation – assembly task |  |  |
| - Action observation test using sticks, nuts and bolts (simple and complex). | Imitation and execution score | (Bieber et al., 2021) |
| Representational gesture observation and recognition |  |  |
| - Recognition - Meaningful gestures on verbal command - Meaningful gestures to tool picture - Knowledge of tool use | Accuracy %; total score | (Bieber et al., 2021; Costini et al., 2018) |
| Automatic imitation |  |  |
| - Motor Interference Task | Error value % | (Nobusako et al., 2018) |
| Mirrored movements |  |  |
| - Presence of mirrored movements | No. mirrored movements | (Blais et al., 2018) |
| **3.3 Internal modelling (including prospective reaching/grasping)** | |  |
| Throwing task, prism adaptation |  |  |
| - Displacing wedge prism - Reversing dove prism | Variable error; adaptation; after effect | (Gomez-Moya et al., 2020) |
| Bimanual lifting and unloading |  |  |
| - Imposed unloading - Voluntary unloading | Maximum angular amplitude; duration of voluntary unloading; duration of muscle onset | (Cignetti et al., 2018) |
| Serial Prediction Tasks |  |  |
| - Visuomotor Task - Perceptual Tasks - Control | Response accuracy; EEG % area latency | (Opitz et al., 2020) |
| Delayed Visual Feedback Detection |  |  |
| - Delayed visual feedback detection task | Delayed detection threshold | (Nobusako et al., 2018) |
| Prospective reaching/grasping |  |  |
| - Simple tasks (sword task, bar grasping task, bar transport task) - Complex tasks (hammer task, octagon task) with and without motor imagery | End state comfort (all trials, non-critical trials, critical trials); minimal rotation | (Adams et al., 2017, 2017a; Bhoyroo et al., 2018, 2019; Krajenbrink et al., 2021) |
| **3.4 Oculomotor control** |  |  |
| Visual tracking tasks |  |  |
| - Prosaccade (VGST) - Antisaccade task - Delayed saccade task - Memory-guided saccade task - Fixation Task - Smooth pursuit task | Saccade latency; saccade gain; saccade gain variance; saccade velocity; saccade skewness; saccade error; number of saccades; time on target; pursuit duration; accuracy | (Gaymard et al., 2017; Sumner et al., 2018a) |
| **3.5 Reaching and manual control** |  |  |
| Target directed coordination. Effect of pre cues on planning target-directed eye-movements. |  |  |
| - Target-directed Eye Movement - Target-directed Hand Movement - Target-directed Eye & Hand | Eye movements: Saccade latency; saccade error; fixation errors; inhibition errors  Hand movements: Touch time; accuracy | (Gonzalez et al., 2016) |
| Target directed pointing |  |  |
| - Pointing movement along mid-sagittal plane | Variable error; constant error; movement time; variability in coordination pattern | (Golenia et al., 2018) |
| Manual cup stacking task: Hand-eye coordination |  |  |
| - Unimanual stacking - Bimanual stacking | Hand movements: Movement time (reach, displacement, total)  Eye movements: No. fixations on AOI; fixation duration on AOI; no. fixations per trial; no. fixations per second | (Warlop et al., 2020a) |
| Target-directed aiming using stylus |  |  |
| - Valid cue - Invalid cue - Neutral cue | Reaction time; movement time; deceleration time; movement unit | (Gama et al., 2016) |
| Manual tracking |  |  |
| - Steering/guiding a grommet on a wire “track” of differing complexity | Quality scores; completion time; maze summary score | (Hsu et al., 2018) |
| Force control. Kinetics of manual and leg force control. |  |  |
| - Peak manual force - With visual feedback - Without visual feedback - Retention test | Maximum force; RMSE; CV | (da Rocha Diz et al., 2018) |
| **3.6 Catching** |  |  |
| Two handed catching |  |  |
| - Performance - Kinematics - Eye movement during object recognition and object tracking | No. correct catches; grasping errors  Time to initiate movement; movement time  % fixation time on AOI (ball, experimenter, ball machine, other) | (Licari et al., 2018) |
| Virtual Reality interception |  |  |
| - Performance - Kinematics | VR game score  Hand path length | (Wattad et al., 2020) |
| **3.7 Gait (including visual control of gait and clinical assessment)** | |  |
| Regular terrain |  |  |
| - Stability - Temporal - Spatial - Speed | Step length and step time ratio, difference, and variability  Single support %; load response %; pre-swing %  Proportional step length; step width; double support; head angle; trunk angle  Velocity; walk time | (Gentle et al., 2016; Nieto et al., 2018; Schott et al., 2016; Wilmut, Gentle, et al., 2017c) |
| Irregular terrain |  |  |
| - Spatial - Speed | Proportional step length; step width; double support; head angle; trunk angle (% change from regular terrain)  Velocity | (Gentle et al., 2016) |
| Treadmill walking |  |  |
| - Stability - Speed (preferred) - Leg muscle activation | Normalised and raw RMS accelerations and Lyapunov exponent (VL, ML, AP)  Walking speed  Peak EMG (heel strike, loading response, mid stance, late stance, toe off, early swing, mid swing, and late swing (rectus femoris, biceps femoris, tibialis anterior, gastrocnemius medialis)) | (Speedtsberg et al., 2018; Yam and Fong, 2018) |
| Regular terrain with restricted vision |  |  |
| - Intermittent vision - No vision | Single support; load response; pre-swing | (Nieto et al., 2018) |
| Locomotor pointing – stair ascent/descent |  |  |
| - Stability - Temporal - Spatial - Speed | Handrail use; AP MoS  Step duration; step duration variability  Vertical/horizontal toe clearance  Ascent/descent duration | (Parr et al., 2020a) |
| Locomotor pointing – target-directed stepping |  |  |
| - Stability - Spatial - Approach speed | ML COM velocity (maximal and variability)  No. errors; no. box contacts; ML and AP error (absolute, constant, variable); step length and width variability  Approach speed | (Parr et al., 2020; Schott et al., 2016) |
| Locomotor pointing – stepping-stone task |  |  |
| - Temporal | Swing time; stance time; total movement time | (Warlop et al., 2020) |
| Locomotor pointing – obstacle avoidance |  |  |
| - Anticipation - Circumvention - Foot placement adjustments | Step length; step width; velocity and acceleration (ML, AP, VL)  Time and distance before deviation; size of deviation; velocity and acceleration (ML, AP, VL)  % adjustment steps used | (Wilmut and Barnett, 2017b, 2017a) |
| Visual gaze during gait |  |  |
| - Stair ascent/descent – eye movement - Target-directed stepping – general fixation - Target-directed stepping – proximal AOI - Target-directed stepping – distal AOI - Target-directed stepping – target AOI | Fixation rate; fixation duration; steps ahead; gaze time to areas  Fixation rate; fixation duration; no. fixations; gaze shifts toward/away from target; gaze transfers | (Parr et al., 2020, 2020a; Warlop et al., 2020) |
| Clinical assessment of gait |  |  |
| - Functional gait assessment - Ataxia gait assessment | pmFGA movement quality score; number steps before losing balance; summary score  SARA total score and gait subscore | (Hsu et al., 2018; Mannini et al., 2017) |
| **3.8 Postural control** |  |  |
| Static – full vision |  |  |
| - Bipedal – firm surface - Unipedal – firm surface - Bipedal – compliant surface | COP displacement and variability (ML, AP); sway length; rambling and trembling (ML, AP) | (Ganapathy Sankar & Monisha, 2019; Nunzi et al., 2018; Speedtsberg et al., 2017) |
| Static – restricted vision |  |  |
| - Bipedal – firm surface - Unipedal – firm surface - Bipedal – compliant surface - Bipedal – compliant surface EMG | COP displacement and variability (ML, AP); body sway (ML, AP); reactive balance performance; rambling and trembling (ML, AP)  Muscle onset latency (hamstrings, gastrocnemius, rectus femoris, tibialis anterior) | (Chen et al., 2019; Cheng et al., 2018; Ganapathy Sankar & Monisha, 2019; Nunzi et al., 2018; Speedtsberg et al., 2017) |
| Dynamic |  |  |
| - WiiFit - Virtual Reality - Y-balance test - Y-balance test – EMG | Movement score (head, upper trunk, arm, hip, feet); gates passed; score  COP velocity, acceleration, and range (ML, AP); COP path length  Normalised scores  Peak and time to peak EMG (rectus femoris, biceps femoris, tibialis anterior, gastrocnemius medialis) | (Jelsma et al., 2020; Miller et al., 2019; Wattad et al., 2020; Yam and Fong, 2019) |
| **3.9** **Dual-Task performance** |  |  |
| Motor-cognitive dual-task |  |  |
| - Locomotor-cognitive (trail *walking* test) – high and low cognitive load - Manual-cognitive (trail *making* test) – high and low cognitive load | Completion time; no. errors; motor dual-task costs; cognitive dual-task costs | (Schott et al., 2016) |
| **3.10 Attentional focus and motor learning** |  |  |
| Non-learning paradigm – pole stability |  |  |
| - Internal focus of attention - External focus of attention - No focus of attention | Pole displacement (AP, ML, VL); pole rotation (yaw, pitch, roll); COP sway (AP, ML) | (Li et al., 2019) |
| Non-learning paradigm – jumping task |  |  |
| - Internal focus of attention - External focus of attention - Combined focus of attention | Jump height; trochanter take-off velocity; trochanter vertical displacement; minimal knee angle; knee angle at take-off; knee angular displacement | (Psotta et al., 2020) |
| Learning paradigm – focus of attention |  |  |
| - Internal focus of attention - External focus of attention | Dart throwing accuracy | (Khatab et al., 2018) |
| Learning paradigm – stages of learning |  |  |
| - Baseline - Consolidation - Retention - Near transfer - Far transfer | Game score (Wii ski-slalom game); gates missed; time to finish  No. sessions to reach stable performance (Wii tennis); no. practice sessions (Wii archery); invented letter task production time, time on page, time off page, accuracy, number of segments, dysfluency  Short-term and long-term learning score (Wii ski-slalom)  MABC balance score | (Adi-Japha and Brestel, 2020; de Carvalho et al., 2020; Smits-Engelsman et al., 2020) |
|  |  |  |
| **4 Neuroimaging** |  |  |
| **4.1 Structural MRI** |  |  |
| White matter microstructure |  |  |
| - MRI Diffusion Tensor Imaging - Constrained Spherical Deconvolution | Mean tract volume; Mean FA; MD; AFD; white matter volume | (Brown-Lum et al., 2020; Hyde et al., 2019; Reynolds et al., 2017a; Williams et al., 2017) |
| Grey matter volume |  |  |
| - Voxel-based morphometry | Grey matter volume; total intracranial volume; cortical thickness | (Lê et al., 2021; Reynolds et al., 2017a) |
| **4.2 Functional MRI** |  |  |
| Resting State Functional Connectivity |  |  |
| - Whole brain MRI | Independent Component Analysis | (Rinat et al., 2020) |
| During tasks |  |  |
| - Motor imagery - Action observation and imitation - Fine motor tasks - Cognitive control (inhibition) | Mean BOLD signal % change  BOLD signal  fNIRS  OxyHb; BOLD activation patterns | (Cacola et al., 2018; Kashuk et al., 2017; Koch et al., 2018; Reynolds et al., 2019; Thornton et al., 2018) |
| **4.3 EEG / Neurophysiological** |  |  |
| Cortical inhibition |  |  |
| - Motor thresholds - Intra-hemispheric cortical inhibition - Inter-hemispheric cortical inhibition | Resting-motor threshold; active-motor threshold; MEP  SICI; LICI; CSP ratio  ISP ratio | (He, Fuelscher, Enticott, et al., 2018a) |
| Corticospinal excitability |  |  |
| - Motor imagery | MEP amplitude | (Hyde et al., 2018) |
| Action observation and imitation |  |  |
| - Detection and imitation task | Mu power; Mu coherence | (Lust et al., 2019) |
| Action preparation |  |  |
| - Visual task-irrelevant probe - Tactile task-irrelevant probe - Movement-cue processing | ERP mean peak amplitude P1 and N1  ERP mean peak amplitude P60 and N140  Beta oscillation | (Job et al., 2019) |
| Attention during motor performance |  |  |
| - MABC - Unexpected perturbation | EEG derived attention | (Cheng et al., 2018; Fong et al., 2016) |
| Bimanual coordination |  |  |
| - Learning a finger tapping pattern | Inter-hemispheric task-related coherence | (Blais et al., 2018) |
| Motor inhibition |  |  |
| - Go/Nogo paradigm | ERP mean peak amplitude at N2 and P3 | (Suzuki et al., 2020) |
| Visuospatial working memory |  |  |
| - Delayed and non-delay visual matching task | Theta power; alpha power | (Wang et al., 2017) |

Note: TVPS, Test of Visual Perception Score; VMI, Test of Visual-Motor Integration; ROF, Rey-Osterrieth figure; HTKS, Head-Toes-Knees-Shoulders; VIMI, Verbal Inhibition Motor Inhibition task; GMLT, Groton Maze Learning Task; WCST-64, Wisconsin Card Sort Task; WISC, Wechsler Intelligence Scales for Children; C-VRFT, Computerized Visual Radial Fitts’ Task; FPIQ, Florida Praxis Imagery Questionnaire; MIQ-c, Movement Imagery Questionnaire – Children; AO, action observation; MI, motor imagery; EEG, electroencephalograph; VGST, visually-guided saccade task; AOI, Area of interest; RMSE, root mean squared error; CV, coefficient of variation; VR, virtual reality; RMS, root mean squared; VL, vertical; ML, mediolateral; AP, anteroposterior; EMG, electromyography; MoS, margin of stability; COM, centre of mass; pmFGA, Pediatric-modified Functional Gait Assessment; SARA, Scale for Assessment of Ataxia Severity; COP, centre of pressure; MABC, Movement Assessment Battery for Children; MRI, Magnetic Resonance Imaging; MD, mean diffusivity; AFD, apparent fibre density; BOLD, Blood-Oxygen-Level-Dependent; fNIRS, functional near-infrared spectroscopy; OxyHb, oxyhemoglobin; MEP, motor-evoked potential; SICI, short-interval cortical inhibition; LICI, long-interval cortical inhibition; CSP ratio, cortical silent period ration; ISP ratio, ipsilateral silent period ratio; ERP, event-related potential.

**References**

Adams, I.L.J., Lust, J.M., Steenbergen, B., 2018. Development of motor imagery ability in children with developmental coordination disorder – A goal-directed pointing task. Br. J. Psychol. 109, 187–203. https://doi.org/10.1111/bjop.12274

Adams, I.L.J., Lust, J.M., Wilson, P.H., Steenbergen, B., 2017. Development of motor imagery and anticipatory action planning in children with developmental coordination disorder – A longitudinal approach. Hum. Mov. Sci. 55, 296–306. https://doi.org/10.1016/j.humov.2017.08.021

Adams, I.L.J., Lust, J.M., Wilson, P.H., Steenbergen, B., 2017a. Testing predictive control of movement in children with developmental coordination disorder using converging operations. Br. J. Psychol. 108, 73–90. https://doi.org/10.1111/bjop.12183

Adi-Japha, E., Brestel, G., 2020. Motor skill learning with impaired transfer by children with developmental coordination disorder. Res. Dev. Disabil. 103. https://doi.org/10.1016/j.ridd.2020.103671

Alesi, M., Gómez-López, M., Bianco, A., 2019. Motor differentiation’s and cognitive skill in pre-scholar age. Cuad. Psicol. Deporte 19, 50–59. https://doi.org/10.6018/cpd.338341

Alesi, M., Pecoraro, D., Pepi, A., 2019a. Executive functions in kindergarten children at risk for developmental coordination disorder. Eur. J. Spec. Needs Educ. 34, 285–296. https://doi.org/10.1080/08856257.2018.1468635

Barbacena, M.M., Valladao Novais Van Petten, A.M., Ferreira, D.L., Magalhaes, L. de C., 2019. Cognitive level and developmental coordination disorder: study with schoolchildren aged 7 to 10 years old. Braz. J. Occup. Ther. 27, 534–544. https://doi.org/10.4322/2526-8910.ctoAO1839

Bernardi, M., Leonard, H.C., Hill, E.L., Botting, N., Henry, L.A., 2018. Executive functions in children with developmental coordination disorder: a 2-year follow-up study. Dev. Med. Child Neurol. 60, 306–313. https://doi.org/10.1111/dmcn.13640

Bhoyroo, R., Hands, B., Wilmut, K., Hyde, C., Wigley, A., 2019. Motor planning with and without motor imagery in children with Developmental Coordination Disorder. Acta Psychol. (Amst.) 199. https://doi.org/10.1016/j.actpsy.2019.102902

Bhoyroo, R., Hands, B., Wilmut, K., Hyde, C., Wigley, A., 2018. Investigating motor planning in children with DCD: Evidence from simple and complex grip-selection tasks. Hum. Mov. Sci. 61, 42–51. https://doi.org/10.1016/j.humov.2018.07.006

Bieber, E., Smits-Engelsman, B.C.M., Sgandurra, G., Di Gregorio, F., Guzzetta, A., Cioni, G., Feys, H., Klingels, K., 2021. A new protocol for assessing action observation and imitation abilities in children with Developmental Coordination Disorder: A feasibility and reliability study. Hum. Mov. Sci. 75. https://doi.org/10.1016/j.humov.2020.102717

Blais, M., Amarantini, D., Albaret, J.-M., Chaix, Y., Tallet, J., 2018. Atypical inter-hemispheric communication correlates with altered motor inhibition during learning of a new bimanual coordination pattern in developmental coordination disorder. Dev. Sci. 21. https://doi.org/10.1111/desc.12563

Blais, M., Baly, C., Biotteau, M., Albaret, J.-M., Chaix, Y., Tallet, J., 2017. Lack of Motor Inhibition as a Marker of Learning Difficulties of Bimanual Coordination in Teenagers With Developmental Coordination Disorder. Dev. Neuropsychol. 42, 207–219. https://doi.org/10.1080/87565641.2017.1306526

Brown-Lum, M., Izadi-Najafabadi, S., Oberlander, T.F., Rauscher, A., Zwicker, J.G., 2020. Differences in white matter microstructure among children with developmental coordination disorder. Jama Netw. Open 3. https://doi.org/10.1001/jamanetworkopen.2020.1184

Cacola, P., Getchell, N., Srinivasan, D., Alexandrakis, G., Liu, H., 2018. Cortical activity in fine-motor tasks in children with Developmental Coordination Disorder: A preliminary fNIRS study. Int. J. Dev. Neurosci. 65, 83–90. https://doi.org/10.1016/j.ijdevneu.2017.11.001

Chen, F.-C., Li, L.-L., Chu, C.-H., Pan, C.-Y., Tsai, C.-L., 2019. Finger soaking enhances effects of light touch on reducing body sway in children with developmental coordination disorder. J. Rehabil. Med. 51, 217–224. https://doi.org/10.2340/16501977-2524

Chen, F.-C., Pan, C.-Y., Chu, C.-H., Tsai, C.-L., Tseng, Y.-T., 2020. Joint Position Sense of Lower Extremities is Impaired and Correlated with Balance Function in Children with Developmental Coordination Disorder. J. Rehabil. Med. 52, 1–9. https://doi.org/10.2340/16501977-2720

Cheng, Y.T.Y., Tsang, W.W.N., Schooling, C.M., Fong, S.S.M., 2018. Reactive balance performance and neuromuscular and cognitive responses to unpredictable balance perturbations in children with developmental coordination disorder. Gait Posture 62, 20–26. https://doi.org/10.1016/j.gaitpost.2018.02.025

Cignetti, F., Vaugoyeau, M., Fontan, A., Jover, M., Livet, M.-O., Hugonenq, C., Audic, F., Chabrol, B., Assaiante, C., 2018. Feedforward motor control in developmental dyslexia and developmental coordination disorder: Does comorbidity matter? Res. Dev. Disabil. 76, 25–34. https://doi.org/10.1016/j.ridd.2018.03.001

Costini, O., Roy, A., Faure, S., Remigereau, C., Renaud, E., Blanvillain, L., Fossoud, C., Le Gall, D., 2018. Gestures and related skills in developmental coordination disorder: A production-system deficit? Psychol. Neurosci. 11, 193–215. https://doi.org/10.1037/pne0000115

da Rocha Diz, M.A., Ferracioli, M. de C., Hiraga, C.Y., de Oliveira, M.A., Pellegrini, A.M., 2018. Effects of practice on visual finger-force control in children at risk of developmental coordination disorder. Braz. J. Phys. Ther. 22, 467–473. https://doi.org/10.1016/j.bjpt.2018.04.002

de Carvalho, L.S., Crancianinov, C.S.A., Gama, D.T., Hiraga, C.Y., 2020. Effect of volume of practice in children with probable developmental coordination disorder. Rev. Bras. Cineantropometria E Desempenho Hum. 22, 1–10. https://doi.org/10.1590/1980-0037.2020v22e72028

de Waal, E., Pienaar, A.E., Coetzee, D., 2018. Perceptual-motor contributors to the association between developmental coordination disorder and academic performance: North-West Child Health, Integrated with Learning and Development study. South Afr. J. Child. Educ. 8. https://doi.org/10.4102/sajce.v8i2.562

Fong, S.S.M., Chung, J.W.Y., Cheng, Y.T.Y., Yam, T.T.T., Chiu, H.-C., Fong, D.Y.T., Cheung, C.Y., Yuen, L., Yu, E.Y.T., Hung, Y.S., Macfarlane, D.J., Ng, S.S.M., 2016. Attention during functional tasks is associated with motor performance in children with developmental coordination disorder: A cross-sectional study. Medicine (Baltimore) 95. https://doi.org/10.1097/MD.0000000000004935

Fuchs, C.T., Cacola, P., 2018. Differences in accuracy and vividness of motor imagery in children with and without Developmental Coordination Disorder. Hum. Mov. Sci. 60, 234–241. https://doi.org/10.1016/j.humov.2018.06.015

Gama, D.T., Ferracioli, M.D.C., Hiraga, C.Y., Pellegrini, A.M., 2016. Value of pre-cue information for motor tasks performed by children with developmental coordination disorder (DCD). Mot. Rev. Educ. Fis. 22, 138–143. https://doi.org/10.1590/S1980-6574201600030004

Ganapathy Sankar, U., Monisha, R., 2019. Assessment of balance in children with developmental coordination disorder in Indian context. Indian J. Public Health Res. Dev. 10, 67–70. https://doi.org/10.5958/0976-5506.2019.01538.9

Gauthier, S., Anzalone, S.M., Cohen, D., Zaoui, M., Chetouani, M., Villa, F., Berthoz, A., Xavier, J., 2018. Behavioral Own-Body-Transformations in Children and Adolescents With Typical Development, Autism Spectrum Disorder, and Developmental Coordination Disorder. Front. Psychol. 9. https://doi.org/10.3389/fpsyg.2018.00676

Gaymard, B., Giannitelli, M., Challes, G., Rivaud-Pechoux, S., Bonnot, O., Cohen, D., Xavier, J., 2017. Oculomotor Impairments in Developmental Dyspraxia. Cerebellum 16, 411–420. https://doi.org/10.1007/s12311-016-0817-6

Gentle, J., Barnett, A.L., Wilmut, K., 2016. Adaptations to walking on an uneven terrain for individuals with and without Developmental Coordination Disorder. Hum. Mov. Sci. 49, 346–353. https://doi.org/10.1016/j.humov.2016.08.010

Ghotbi, M., Sohrabi, M., Taheri, H.R., Khodashenas, E., 2016. The comparison of depth perception in 7-9 years old healthy children with developmental coordination disorder. J. Ecophysiol. Occup. Health 16, 138–143. https://doi.org/10.15512/joeoh/2016/v16i3&4/15461

Golenia, L., Bongers, R.M., van Hoorn, J.F., Otten, E., Mouton, L.J., Schoemaker, M.M., 2018. Variability in coordination patterns in children with developmental coordination disorder (DCD). Hum. Mov. Sci. https://doi.org/10.1016/j.humov.2018.06.009

Gomez-Moya, R., Diaz, R., Vaca-Palomares, I., Fernandez-Ruiz, J., 2020. Procedural and strategic visuomotor learning deficits in children with developmental coordination disorder. Res. Q. Exerc. Sport. https://doi.org/10.1080/02701367.2019.1675852

Gonzalez, C.C., Mon-Williams, M., Burke, S., Burke, M.R., 2016. Cognitive control of saccadic eye movements in children with developmental coordination disorder. PloS One. https://doi.org/10.1371/journal.pone.0165380

He, J.L., Fuelscher, I., Coxon, J., Barhoun, P., Parmar, D., Enticott, P.G., Hyde, C., 2018. Impaired motor inhibition in developmental coordination disorder. Brain Cogn. 127, 23–33. https://doi.org/10.1016/j.bandc.2018.09.002

He, J.L., Fuelscher, I., Enticott, P.G., Teo, W., Barhoun, P., Hyde, C., 2018a. Interhemispheric cortical inhibition is reduced in young adults with developmental coordination disorder. Front. Neurol. 9. https://doi.org/10.3389/fneur.2018.00179

Hsu, L.-Y., Jirikowic, T., Ciol, M.A., Clark, M., Kartin, D., McCoy, S.W., 2018. Motor planning and gait coordination assessments for children with developmental coordination disorder. Phys. Occup. Ther. Pediatr. 38, 562–574. https://doi.org/10.1080/01942638.2018.1477226

Hyde, C., Fuelscher, I., Williams, J., Lum, J.A.G., He, J., Barhoun, P., Enticott, P.G., 2018. Corticospinal excitability during motor imagery is reduced in young adults with developmental coordination disorder. Res. Dev. Disabil. 72, 214–224. https://doi.org/10.1016/j.ridd.2017.11.009

Hyde, C., Ian, F., Peter, E.G., Derek, J.K., Shawna, F., Tim, S.J., Jacqueline, W., Karen, C., 2019. White matter organization in developmental coordination disorder: A pilot study exploring the added value of constrained spherical deconvolution. Neuroimage-Clin. 21. https://doi.org/10.1016/j.nicl.2018.101625

Jelsma, L.D., Geuze, R.H., Smits-Engelsman, B.C.M., 2020. Movement control strategies in a dynamic balance task in children with and without developmental coordination disorder. J. Mot. Behav. 52, 175–186. https://doi.org/10.1080/00222895.2019.1599809

Job, X.E., Bradya, D., de Fockert, J.W., Luft, C.D.B., Hill, E.L., van Velzen, J., 2019. Adults with probable developmental coordination disorder selectively process early visual, but not tactile information during action preparation. An electrophysiological study. Hum. Mov. Sci. 66, 631–644. https://doi.org/10.1016/j.humov.2019.02.018

Johnston, J.S., Ali, J.B., Hill, E.L., Bremner, A.J., 2017. Tactile localization performance in children with developmental coordination disorder (DCD) corresponds to their motor skill and not their cognitive ability. Hum. Mov. Sci. 53, 72–83. https://doi.org/10.1016/j.humov.2016.12.008

Kashuk, S.R., Williams, J., Thorpe, G., Wilson, P.H., Egan, G.F., 2017. Diminished motor imagery capability in adults with motor impairment: An fMRI mental rotation study. Behav. Brain Res. 334, 86–96. https://doi.org/10.1016/j.bbr.2017.06.042

Khatab, S.F., Ghasemi, A., Sadati, S.K.M., 2018. The Effect of Focus Instructions on Dart Throwing Performance in Children With and Without Developmental Coordination Disorder. Ann. Appl. Sport Sci. 6, 55–60.

Koch, J.K.L., Miguel, H., Smiley-Oyen, A.L., 2018. Prefrontal activation during Stroop and Wisconsin card sort tasks in children with developmental coordination disorder: a NIRS study. Exp. Brain Res. 236, 3053–3064. https://doi.org/10.1007/s00221-018-5358-4

Krajenbrink, H., Lust, J.M., Steenbergen, B., 2021. Eliciting End-State Comfort Planning in Children With and Without Developmental Coordination Disorder Using a Hammer Task: A Pilot Study. Front. Psychol. 12. https://doi.org/10.3389/fpsyg.2021.625577

Lê, M., Blais, M., Jucla, M., Chauveau, N., Maziero, S., Biotteau, M., Albaret, J., Péran, P., Chaix, Y., Tallet, J., 2021. Procedural learning and retention of audio‐verbal temporal sequence is altered in children with developmental coordination disorder but cortical thickness matters. Dev. Sci. 24, 1–14. https://doi.org/10.1111/desc.13009

Li, L.-L., Li, Y.-C., Chu, C.-H., Pan, C.-Y., Chen, F.-C., 2019. External focus of attention concurrently elicits optimal performance of suprapostural pole-holding task and postural stability in children with developmental coordination disorder. Neurosci. Lett. 703, 32–37. https://doi.org/10.1016/j.neulet.2019.03.011

Licari, M.K., Reynolds, J.E., Tidman, S., Ndiaye, S., Sekaran, S.N., Reid, S.L., Lay, B.S., 2018. Visual tracking behaviour of two-handed catching in boys with developmental coordination disorder. Res. Dev. Disabil. 83, 280–286. https://doi.org/10.1016/j.ridd.2018.07.005

Lust, J.M., van Schie, H.T., Wilson, P.H., van der Helden, J., Pelzer, B., Steenbergen, B., 2019. Activation of mirror neuron regions is altered in Developmental Coordination Disorder (DCD)–neurophysiological evidence using an action observation paradigm. Front. Hum. Neurosci. 13. https://doi.org/10.3389/fnhum.2019.00232

Mannini, A., Martinez-Manzanera, O., Lawerman, T.F., Trojaniello, D., Della Croce, U., Sival, D.A., Maurits, N.M., Sabatini, A.M., 2017. Automatic classification of gait in children with early-onset ataxia or developmental coordination disorder and controls using inertial sensors. Gait Posture 52, 287–292. https://doi.org/10.1016/j.gaitpost.2016.12.002

Michel, E., Molitor S., Schneider W., 2018. Differential changes in the development of motor coordination and executive functions in children with motor coordination impairments. Child Neuropsychol. 24, 20–45. https://doi.org/10.1080/09297049.2016.1223282

Miller, H.L., Cacola, P.M., Sherrod, G.M., Patterson, R.M., Bugnariu, N.L., 2019. Children with Autism Spectrum Disorder, Developmental Coordination Disorder, and typical development differ in characteristics of dynamic postural control: A preliminary study. Gait Posture 67, 9–11. https://doi.org/10.1016/j.gaitpost.2018.08.038

Mirabella, G., Del Signore, S., Lakens, D., Averna, R., Penge, R., Capozzi, F., 2017. Developmental coordination disorder affects the processing of action-related verbs. Front. Hum. Neurosci. 10. https://doi.org/10.3389/fnhum.2016.00661

Nieto, M.P., Valtr, L., Abdollahipour, R., Agricola, A., Psotta, R., 2018. The role of vision in walking patterns in children with different levels of motor coordination. Rev. Iberoam. Psicol. Ejerc. El Deporte 13, 289–296.

Nobusako, S., Osumi, M., Furukawa, E., Nakai, A., Maeda, T., Morioka, S., 2021. Increased visual bias in children with developmental coordination disorder: Evidence from a visual-tactile temporal order judgment task. Hum. Mov. Sci. 75, 102743. https://doi.org/10.1016/j.humov.2020.102743

Nobusako, S., Sakai, A., Tsujimoto, T., Shuto, T., Nishi, Y., Asano, D., Furukawa, E., Zama, T., Osumi, M., Shimada, S., Morioka, S., Nakai, A., 2018. Deficits in visuo-motor temporal integration impacts manual dexterity in probable developmental coordination disorder. Front. Neurol. 9. https://doi.org/10.3389/fneur.2018.00114

Nunzi, M., Sylos Labini, F., Meli, A., Baldi, S., Tufarelli, D., Di Brina, C., 2018. Static balance performance and sensory integration abilities of children with dyslexia and developmental coordination disorder, in: da Silva H.P., Constantine L., Escalona M.J., Ramirez A.J., Helfert M. (Eds.), CHIRA - Proc. Int. Conf. Computer-Human Interact. Res. Appl. SciTePress, pp. 150–155. https://doi.org/10.5220/0006930601500155

Opitz, B., Brady, D., Leonard, H.C., 2020. Motor and non-motor sequence prediction is equally affected in children with developmental coordination disorder. PloS One 15, e0232562. https://doi.org/10.1371/journal.pone.0232562

Parr, J.V.V., Foster, R.J., Wood, G., Hollands, M.A., 2020. Children with developmental coordination disorder exhibit greater stepping error despite similar gaze patterns and state anxiety levels to their typically developing peers. Front. Hum. Neurosci. 14. https://doi.org/10.3389/fnhum.2020.00303

Parr, J.V.V., Foster, R.J., Wood, G., Thomas, N.M., Hollands, M.A., 2020a. Children with developmental coordination disorder show altered visuomotor control during stair negotiation associated with heightened state anxiety. Front. Hum. Neurosci. 14. https://doi.org/10.3389/fnhum.2020.589502

Prunty, M., Barnett, A.L., Wilmut, K., Plumb, M., 2016. Visual perceptual and handwriting skills in children with Developmental Coordination Disorder. Hum. Mov. Sci. 49, 54–65. https://doi.org/10.1016/j.humov.2016.06.003

Psotta, R., Abdollahipour, R., Janura, M., 2020. The Effects of Attentional Focus Instruction on the Performance of a Whole-Body Coordination Task in Children With Developmental Coordination Disorder. Res. Dev. Disabil. 101. https://doi.org/10.1016/j.ridd.2020.103654

Purcell, C., Wilmut, K., Wann, J.P., 2017. The use of visually guided behaviour in children with Developmental Coordination Disorder (DCD) when crossing a virtual road. Hum. Mov. Sci. 53, 37–44. https://doi.org/10.1016/j.humov.2016.11.007

Rahimi-Golkhandan, S., Steenbergen, B., Piek, J.P., Caeyenberghs, K., Wilson, P.H., 2016. Revealing hot executive function in children with motor coordination problems: What’s the go? Brain Cogn. 106, 55–64. https://doi.org/10.1016/j.bandc.2016.04.010

Reynolds, J.E., Billington, J., Kerrigan, S., Williams, J., Elliott, C., Winsor, A.M., Codd, L., Bynevelt, M., Licari, M.K., 2019. Mirror neuron system activation in children with developmental coordination disorder: A replication functional MRI study. Res. Dev. Disabil. 84, 16–27. https://doi.org/10.1016/j.ridd.2017.11.012

Reynolds, J.E., Kerrigan, S., Elliott, C., Lay, B.S., Licari, M.K., 2017. Poor imitative performance of unlearned gestures in children with probable developmental coordination disorder. J. Mot. Behav. 49, 378–387. https://doi.org/10.1080/00222895.2016.1219305

Reynolds, J.E., Licari, M.K., Reid, S.L., Elliott, C., Winsor, A.M., Bynevelt, M., Billington, J., 2017a. Reduced relative volume in motor and attention regions in developmental coordination disorder: A voxel-based morphometry study. Int. J. Dev. Neurosci. 58, 59–64. https://doi.org/10.1016/j.ijdevneu.2017.01.008

Rinat, S., Izadi-Najafabadi, S., Zwicker, J.G., 2020. Children with developmental coordination disorder show altered functional connectivity compared to peers. NeuroImage Clin. 27. https://doi.org/10.1016/j.nicl.2020.102309

Roche, R., Viswanathan, P., Clark, J.E., Whitall, J., 2016. Children with developmental coordination disorder (DCD) can adapt to perceptible and subliminal rhythm changes but are more variable. Hum. Mov. Sci. 50, 19–29. https://doi.org/10.1016/j.humov.2016.09.003

Sartori, R.F., Valentini, N.C., Fonseca, R.P., 2020. Executive function in children with and without developmental coordination disorder: A comparative study. Child Care Health Dev. 46, 294–302. https://doi.org/10.1111/cch.12734

Schott, N., El-Rajab, I., Klotzbier, T., 2016. Cognitive-motor interference during fine and gross motor tasks in children with Developmental Coordination Disorder (DCD). Res. Dev. Disabil. 57, 136–148. https://doi.org/10.1016/j.ridd.2016.07.003

Scott, M.W., Emerson, J.R., Dixon, J., Tayler, M.A., Eaves, D.L., 2020. Motor imagery during action observation enhances imitation of everyday rhythmical actions in children with and without developmental coordination disorder. Hum. Mov. Sci. 71. https://doi.org/10.1016/j.humov.2020.102620

Scott, M.W., Emerson, J.R., Dixon, J., Tayler, M.A., Eaves, D.L., 2019. Motor imagery during action observation enhances automatic imitation in children with and without developmental coordination disorder. J. Exp. Child Psychol. 183, 242–260. https://doi.org/10.1016/j.jecp.2019.03.001

Smits-Engelsman, B., Bonney, E., Ferguson, G., 2020. Motor skill learning in children with and without Developmental Coordination Disorder. Hum. Mov. Sci. 74. https://doi.org/10.1016/j.humov.2020.102687

Speedtsberg, M.B., Christensen, S.B., Andersen, K.K., Bencke, J., Jensen, B.R., Curtis, D.J., 2017. Impaired postural control in children with developmental coordination disorder is related to less efficient central as well as peripheral control. Gait Posture 51, 1–6. https://doi.org/10.1016/j.gaitpost.2016.09.019

Speedtsberg, M.B., Christensen, S.B., Stenum, J., Kallemose, T., Bencke, J., Curtis, D.J., Jensen, B.R., 2018. Local dynamic stability during treadmill walking can detect children with developmental coordination disorder. Gait Posture 59, 99–103. https://doi.org/10.1016/j.gaitpost.2017.09.035

Sumner, E., Hutton, S.B., Kuhn, G., Hill, E.L., 2018a. Oculomotor atypicalities in Developmental Coordination Disorder. Dev. Sci. 21. https://doi.org/10.1111/desc.12501

Sumner, E., Pratt, M.L., Hill, E.L., 2016. Examining the cognitive profile of children with Developmental Coordination Disorder. Res. Dev. Disabil. 56, 10–17. https://doi.org/10.1016/j.ridd.2016.05.012

Suzuki, K., Kita, Y., Shirakawa, Y., Egashira, Y., Mitsuhashi, S., Kitamura, Y., Okuzumi, H., Kaga, Y., Inagaki, M., 2020. Reduced Nogo-P3 in adults with developmental coordination disorder (DCD). Int. J. Psychophysiol. 153, 37–44. https://doi.org/10.1016/j.ijpsycho.2020.04.009

Thornton, S., Bray, S., Langevin, L.M., Dewey, D., 2018. Functional brain correlates of motor response inhibition in children with developmental coordination disorder and attention deficit/hyperactivity disorder. Hum. Mov. Sci. 59, 134–142. https://doi.org/10.1016/j.humov.2018.03.018

Tseng, Y.-T., Holst-Wolf, J.M., Tsai, C.-L., Chen, F.-C., Konczak, J., 2019. Haptic perception is altered in children with developmental coordination disorder. Neuropsychologia 127, 29–34. https://doi.org/10.1016/j.neuropsychologia.2019.02.004

Tseng, Y.-T., Tsai, C.-L., Chen, F.-C., Konczak, J., 2018. Wrist position sense acuity and its relation to motor dysfunction in children with developmental coordination disorder. Neurosci. Lett. 674, 106–111. https://doi.org/10.1016/j.neulet.2018.03.031

Tseng, Y.-T., Tsai, C.-L., Chen, F.-C., Konczak, J., 2019a. Position Sense Dysfunction Affects Proximal and Distal Arm Joints in Children with Developmental Coordination Disorder. J. Mot. Behav. 51, 49–58. https://doi.org/10.1080/00222895.2017.1415200

Wang, C.-H., Tseng, Y.-T., Liu, D., Tsai, C.-L., 2017. Neural oscillation reveals deficits in visuospatial working memory in children with developmental coordination disorder. Child Dev. 88, 1716–1726. https://doi.org/10.1111/cdev.12708

Warlop, G., Vansteenkiste, P., Lenoir, M., Deconinck, F.J.A., 2020a. An exploratory study of gaze behaviour in young adults with developmental coordination disorder. Hum. Mov. Sci. 73. https://doi.org/10.1016/j.humov.2020.102656

Warlop, G., Vansteenkiste, P., Lenoir, M., Van Causenbroeck, J., Deconinck, F.J.A., 2020. Gaze behaviour during walking in young adults with developmental coordination disorder. Hum. Mov. Sci. 71. https://doi.org/10.1016/j.humov.2020.102616

Wattad, R., Gabis, L., V., Shefer, S., Tresser, S., Portnoy, S., 2020. Correlations between performance in a virtual reality game and the movement assessment battery diagnostics in children with developmental coordination disorder. Appl. Sci.-Basel 10. https://doi.org/10.3390/app10030833

Williams, J., Kashuk, S.R., Wilson, P.H., Thorpe, G., Egan, G.F., 2017. White matter alterations in adults with probable developmental coordination disorder: an MRI diffusion tensor imaging study. NeuroReport 28, 87–92. https://doi.org/10.1097/WNR.0000000000000711

Wilmut, K., Barnett, A.L., 2017b. When an object appears unexpectedly: foot placement during obstacle circumvention in children and adults with developmental coordination disorder. Exp. Brain Res. 235, 2947–2958. https://doi.org/10.1007/s00221-017-5031-3

Wilmut, K., Barnett, A.L., 2017a. When an object appears unexpectedly: anticipatory movement and object circumvention in individuals with and without Developmental Coordination Disorder. Exp. Brain Res. 235, 1531–1540. https://doi.org/10.1007/s00221-017-4901-z

Wilmut, K., Du, W., Barnett, A.L., 2017. Navigating through apertures: perceptual judgements and actions of children with Developmental Coordination Disorder. Dev. Sci. 20. https://doi.org/10.1111/desc.12462

Wilmut, K., Gentle, J., Barnett, A.L., 2017c. Gait symmetry in individuals with and without developmental coordination disorder. Res. Dev. Disabil. 60, 107–114. https://doi.org/10.1016/j.ridd.2016.11.016

Wilson, P., Ruddock S., Rahimi-Golkhandan S., Piek J., Sugden D., Green D., Steenbergen B., 2020. Cognitive and motor function in developmental coordination disorder. Dev. Med. Child Neurol. https://doi.org/10.1111/dmcn.14646

Yam, T.T.T., Fong, S.S.M., 2019. Y-balance test performance and leg muscle activations of children with developmental coordination disorder. J. Mot. Behav. 51, 385–393. https://doi.org/10.1080/00222895.2018.1485011

Yam, T.T.T., Fong, S.S.M., 2018. Leg muscle activation patterns during walking and leg lean mass are different in children with and without developmental coordination disorder. Res. Dev. Disabil. 73, 87–95. https://doi.org/10.1016/j.ridd.2017.12.014
