# Supplementary material for: Behavioral and Neuroimaging Research on Developmental Coordination Disorder (DCD): A Combined Systematic Review and Meta-Analysis of Recent Findings
Source: Front Psychol. 2022 Jan 27;13:809455. doi: 10.3389/fpsyg.2022.809455 (PMC8829815; doi:10.3389/fpsyg.2022.809455)
Supplement: Supplementary file 1 [file Data_Sheet_1.zip › Supplementary Material - Data Sheet 1/Supplementary Material 2.docx]

**Supplementary Material 2**

Modified CASP criteria for the assessment of methodological quality.

| **Item No.** | **Item description** |
| --- | --- |
| *1* | In the study rationale, is there sufficient acknowledgement of essential aspects of theory and pivotal studies? |
| *2* | Did the study address a clearly focused (theory-driven) question? |
| *3* | Was the task paradigm well chosen to address the research question(s)? |
| *4* | Was sample size sufficient or justified using power calculation? |
| *5* | Were children with DCD identified/screened appropriately and thus (sufficiently) representative of the population? |
| *6* | Were control children/adults representative of the population? |
| *7* | Were the constructs of interest clearly operationalised and measured? |
| *8* | Were major confounds adequately controlled? |
| *9* | Were the statistical methods appropriate and adequately presented? |
| *10* | Are the major implications of the results clearly discussed? |

*k* = number of studies.
