# Supplementary material for: Behavioral and Neuroimaging Research on Developmental Coordination Disorder (DCD): A Combined Systematic Review and Meta-Analysis of Recent Findings
Source: Front Psychol. 2022 Jan 27;13:809455. doi: 10.3389/fpsyg.2022.809455 (PMC8829815; doi:10.3389/fpsyg.2022.809455)
Supplement: Supplementary file 1 [file Data_Sheet_1.zip › Supplementary Material - Data Sheet 1/Supplementary Material 5.pdf]

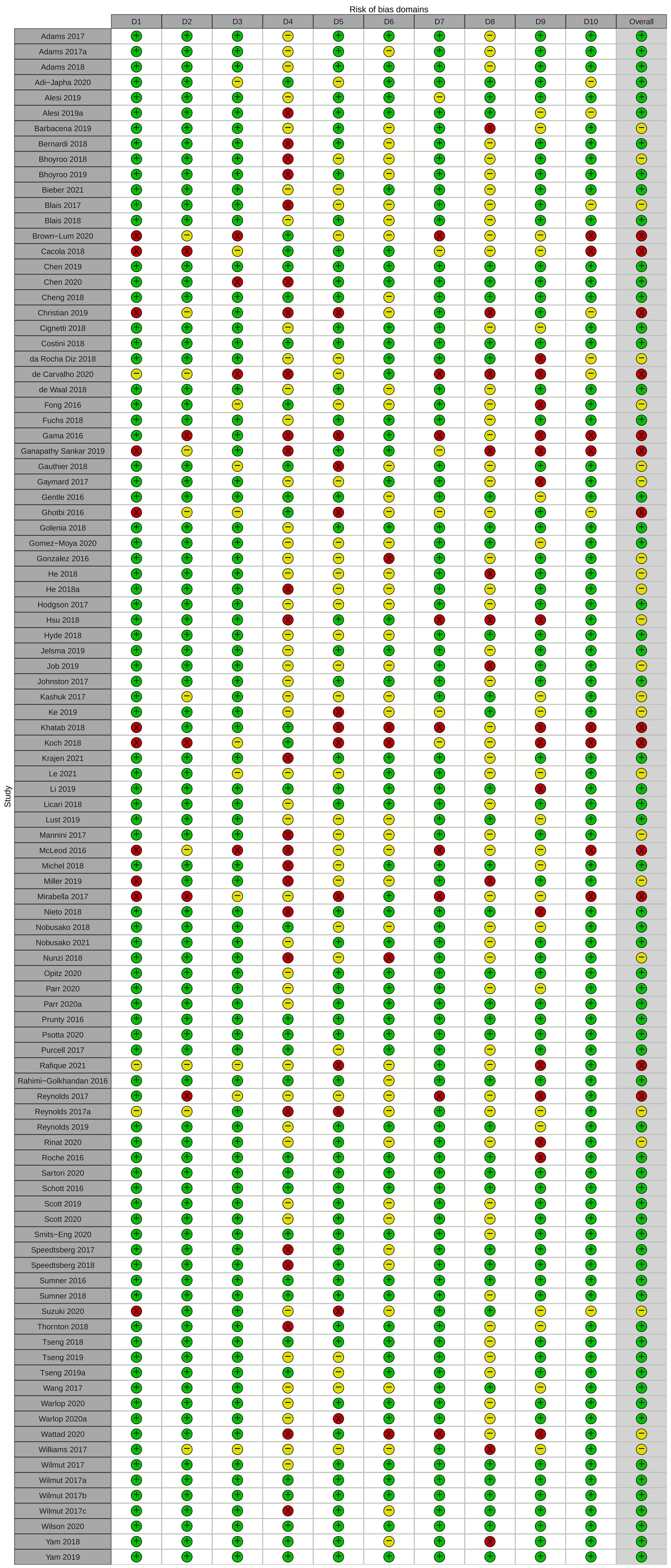

D1: In the study rationale, is there sufficient acknowledgement of essential aspects of theory and pivotal studies?
D2: Did the study address a clearly focused (theory−driven) question?
D3: Was the task paradigm well chosen to address the research question(s)?
D4: Was sample size sufficient or justified using power calculation?
D5: Were children with DCD identified/screened appropriately and thus (sufficiently) representative of the population?
D6: Were control children/adults representative of the population?
D7: Were the constructs of interest clearly operationalised and measured?
D8: Were major confounds adequately controlled?
D9: Were the statistical methods appropriate and adequately presented?
D10: Are the major implications of the results clearly discussed?

Judgement
Low
Unclear
High
Critical
