# Supplementary material for: Behavioral and Neuroimaging Research on Developmental Coordination Disorder (DCD): A Combined Systematic Review and Meta-Analysis of Recent Findings
Source: Front Psychol. 2022 Jan 27;13:809455. doi: 10.3389/fpsyg.2022.809455 (PMC8829815; doi:10.3389/fpsyg.2022.809455)
Supplement: Supplementary file 1 [file Data_Sheet_1.zip › Supplementary Material - Data Sheet 1/Supplementary Material 6.docx]

**Supplementary Material 6**

Characteristics of children with DCD and typically developing children in comparison studies.

|  | Mean | Median | SD | Min | Max | N |
| --- | --- | --- | --- | --- | --- | --- |
| **Sample size** |  |  |  |  |  |  |
| Children with DCD | 23.2 | 18 | 19.2 | 6 | 120 | 100 |
| Typically developing children | 32.7 | 19.5 | 49.4 | 6 | 367 | 100 |
| **Males** |  |  |  |  |  |  |
| Children with DCD (*n*) | 14.9 | 11 | 14 | 2 | 99 | 92 |
| Typically developing children (*n*) | 18 | 11 | 21.7 | 2 | 151 | 91 |
| **Females** |  |  |  |  |  |  |
| Children with DCD (*n*) | 6.9 | 6 | 5.8 | 0 | 24 | 92 |
| Typically developing children (*n*) | 11.7 | 7.0 | 23.8 | 0 | 216 | 91 |
| **Age (years)** |  |  |  |  |  |  |
| Children with DCD | 11.6 | 9.8 | 5.4 | 4.6 | 26.3 | 94 |
| Typically developing children | 11.8 | 10.0 | 5.5 | 4.6 | 27.9 | 93 |

DCD, developmental coordination disorder.
*Note*: For the studies that did not report gender and age splits the values were unable to be calculated, therefore N is not always 100.

*k* = number of studies.
